# UV-vis-NIR magnetic linear dichroism: a powerful complement to MCD for f-block electronic structure

**DOI:** 10.1039/d5sc05890b

**Published:** 2025-11-27

**Authors:** Sydney M. Giles, Kevin O'Neil, Ian E. Ramsier, Gina Angelo, Xin Gui, Wesley J. Transue

**Affiliations:** a Department of Chemistry, University of Pittsburgh Pittsburgh Pennsylvania USA wtransue@pitt.edu

## Abstract

The ability to synthesize next-generation lanthanide and actinide molecular materials with designer photophysical properties rests squarely on our ability to predict, control, and measure their electronic structure. This is especially true of the crystal field (CF) interactions of the metal, which are the only interactions that can be appreciably tuned by ligand design. Herein we present ultraviolet-visible-near infrared magnetic linear dichroism (MLD) spectroscopy as an underutilized magneto-optical technique that holds immense promise in the elucidation of f-block electronic structure. We use a Pr^III^ polyoxometalate complex with pseudo-*D*_4d_ symmetry, [*n*-Bu_4_N]_3_[Pr{Mo_5_O_13_(OMe)_4_(NO)}_2_] (1·Pr), to demonstrate that acquisition of both magnetic circular dichroism (MCD) and MLD spectra allows definitive assignment of the observed CF levels through the complementary selection rules of these techniques. We provide general MCD and MLD sign patterns that can be applied to any (pseudo)-*D*_4d_ Pr^III^ complex to facilitate the assignment of fine structure. Our assignments for 1·Pr allow us to fit its transitions with a phenomenological Hamiltonian, providing insight into its CF splitting and solution geometry along with entirely experimentally-derived wavefunctions for its states without use of density functional theory or multireference computational techniques.

## Abstract 2

Combined magnetic circular dichroism and magnetic linear dichroism analysis of lanthanide complexes can provide precise measurements of crystal field splittings.

## Graphical abstract

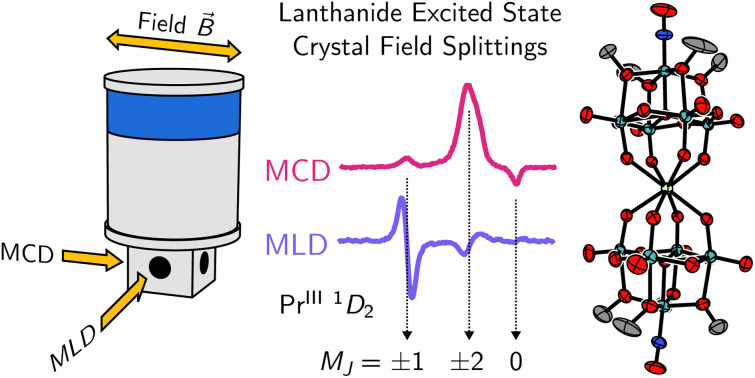

## 1 Introduction

A primary focus of contemporary f-block chemistry is the development of bespoke magnetic, optical, chiroptical, and magneto-optical properties in molecules, including ultranarrow transitions,^1^ circularly polarized luminescence,^2^ magnetochiral dichroism,^3^ spin–electric coupling,^4^ and more.^5^ Crystal field (CF) interactions control all of these properties. The design of next-generation f-block molecular materials requires that synthetic chemists can precisely tune CF interactions about the metal,^6^ and this importance has led to the development of several experimental techniques to characterize the CF. The most popular are ground state (GS) techniques such as magnetometry,^7^ electron paramagnetic resonance,^8^ far-infrared magnetospectroscopy,^9^ and inelastic neutron scattering.^10^ However, there is a complication: strong spin–orbit coupling (SOC) intrinsic to f-block elements causes interstate mixing of |*M*_*J*_〉 levels between energetically proximal states.^11^ This means correlation of GS CF measurements with excited state (ES) properties is not straightforward. It is even known that CF splitting can vary from state to state in ways that cannot be modeled with the usual one-electron CF operators.^12–15^ As f-block chemists increasingly focus on precision engineering of ES properties, it is important to develop new methods to characterize the CF through direct ES observation.

Ultraviolet-visible-near infrared (UV-vis-NIR) magneto-optical techniques like magnetic circular dichroism (MCD) spectroscopy and its sister spectroscopy, magnetic linear dichroism (MLD), are perfectly suited to this task. They offer three main advantages: (1) the signed nature of MCD/MLD features provides greater insight into overlapping absorption bands,^16^ (2) their SOC-driven intensity mechanisms preferentially highlight metal-centered transitions,^17^ and (3) their complementary selection rules to absorption spectroscopy assist in assignments.^18^ MCD in particular has been used in the evaluation of lanthanide electronic structure, both on its own^17^ and in combination with a battery of other experimental techniques.^11^ This is not the case for MLD, which has been almost completely unreported for lanthanide coordination complexes.^19–22^ In fact, MLD is rarely encountered outside of X-ray synchrotron experiments, and we are aware of only a few molecular examples of UV-vis-NIR MLD studies.^22–27^

**Fig. 1:**
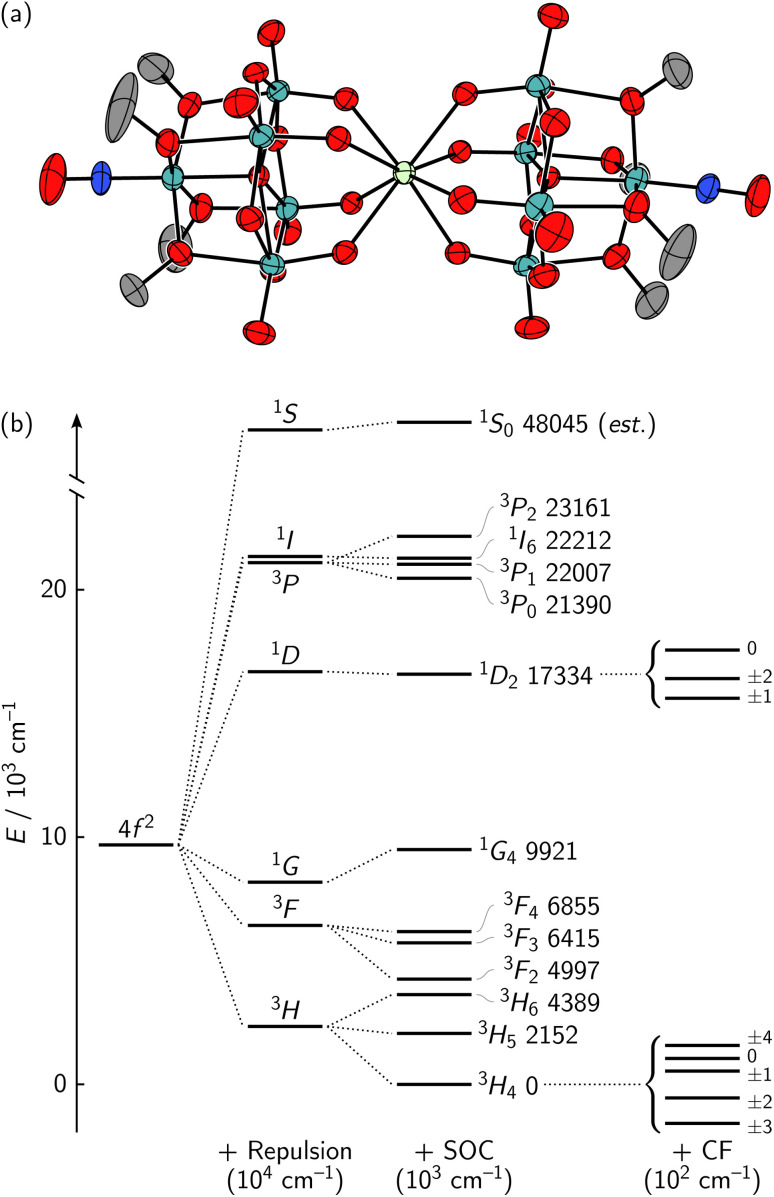
(a) A 50% thermal ellipsoid plot of 1·Pr with the cations and hydrogens omitted for clarity. (b) The energies of all f–f transitions of a free atomic Pr^III^ ion are determined primarily by interelectron repulsion and SOC.^30,31^ There are further CF splittings when Pr^III^ is in a molecule, here shown only for the ^3^H_4_ and ^1^D_2_ levels (CF splitting not to scale).

Combined acquisition of MCD and MLD spectra has the potential to provide great insight into the identities of the GS/ES levels split by the CF due to the different positive/negative intensity patterns between the two techniques. Herein, we demonstrate the power of these combined spectroscopies using an example praseodymium(iii) polyoxometalate (POM) complex [^*n*^Bu_4_N]_3_[Pr{Mo_5_O_13_(OMe)_4_(NO)}_2_] (1·Pr) (Fig. 1a). We outline MCD and MLD selection rules that we have derived for *D*_4d_-symmetric 4f^2^ complexes without the use of Judd–Ofelt theory, which cannot be used at low temperature and is also known to describe Pr^III^ poorly.^28,29^ These selection rules allow unambiguous assignment of the observed fine structure of 1·Pr, and fitting the average CF splitting among the states provides a calculated model that closely agrees with the observed experimental transition energies. This model yields experimentally-determined wavefunctions for the system without need for computational methods like density functional theory (DFT) or multireference computational techniques. Interpretation of these CF parameters using the angular overlap model (AOM) shows that 1·Pr retains a very similar pseudo-*D*_4d_ geometry in solution as in its X-ray crystal structure. The success in modeling the electronic structure of 1·Pr highlights the power of joint acquisition of MCD and MLD spectra to assist in future design of lanthanide and actinide optical materials.

## 2 Theoretical background

### 2.1 Magneto-optical spectroscopies

Due to the shielding of 4f orbitals from metal–ligand interactions and the Laporte selection rule, f–f transitions are characteristically narrow in lineshape and weak in intensity.^29^ Molecules in non-centrosymmetric point groups (like *D*_4d_ and *D*_4_) can display increased f–f intensities through the ‘induced electric dipole’ mechanism arising from CF-driven mixing with Laporte-allowed f–d, f–g, and charge transfer transitions.^32,33^ Through this mechanism, f–f transitions that satisfy the electric dipole selection rule *Γ*_i_ ⊗ *Γ*_m_ ⊗ *Γ*_f_ ∋ *A*_1_ are more likely to be observed in the absorption spectrum, where *Γ*_i_/*Γ*_f_ are the irreducible representations (irreps) of the initial/final states and *Γ*_m_ is the (possibly reducible) representation for the transition dipole moment operator. Unfortunately, the absorption spectrum does not offer many other ways to discriminate between the identities of f–f transitions beyond this selection rule.

Here, dichroic spectroscopies like MCD and MLD offer a distinct advantage. MCD and MLD spectroscopies are techniques that are nominally similar to but physically distinct from their natural circular dichroism (CD) and linear dichroism (LD) counterparts. Natural CD and LD are limited to chiral and/or anisotropic materials, but the addition of a magnetic field induces MCD and MLD signals in all materials, making them more broadly useful.^16^ It is also worth mentioning that MLD is distinct from the Cotton–Mouton effect, in which an applied magnetic field causes molecular reorientation in solution and the development of LD. The great utility of MCD and MLD in f-block spectroscopy comes from the deep connection between these dichroic responses and the molecular symmetry.^34^

MCD and MLD spectroscopies are closely related but differ in the polarization of light and the orientation of the external magnetic field: MCD orients the field parallel with the direction of light and uses left/right-circular polarization (Δ*ε*_MCD_ = *ε*_LCP_ − *ε*_RCP_), whereas MLD orients the field perpendicular to the direction of light and uses linear polarization either parallel or perpendicular to the magnetic field direction (Δ*ε*_MLD_ = *ε*_‖_ − *ε*_⊥_). The intensities of the MCD/MLD signals for a transition *A* → *J* are^35^1
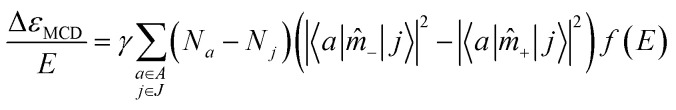
2
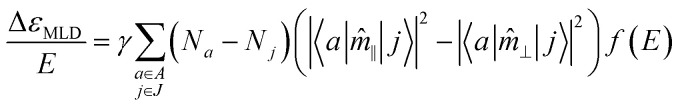
where *E* is energy, *γ* a proportionality constant, *N*_*X*_ the fractional population of level *X*, and *f* a lineshape function. The sums run over all thermally accessible sublevels *a* within GS *A* and all sublevels *j* within ES *J*. The response to left-circular, right-circular, parallel, and perpendicular polarizations of light are calculated from transition dipole moment operators *m̂*_−_, *m̂*_+_, *m̂*_‖_, and *m̂*_⊥_, respectively. Use of eqn (1) and (2) requires the ability to construct a spin Hamiltonian to describe the GS magnetic response,^36–39^ and complicated nonlinear behavior is often encountered when the system experiences magnetic saturation.

Taylor expansion can be used to greatly simplify these equations when the system is far from magnetic saturation (*µ*_B_*B*/*k*_B_*T* ≪ 1, Boltzmann constant *k*_B_, temperature *T*). At sufficiently weak fields or sufficiently high temperatures, Taylor expansion of eqn (1) predicts a linear MCD response,3
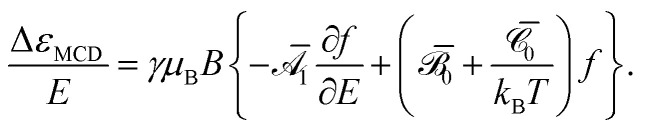


**Fig. 2:**
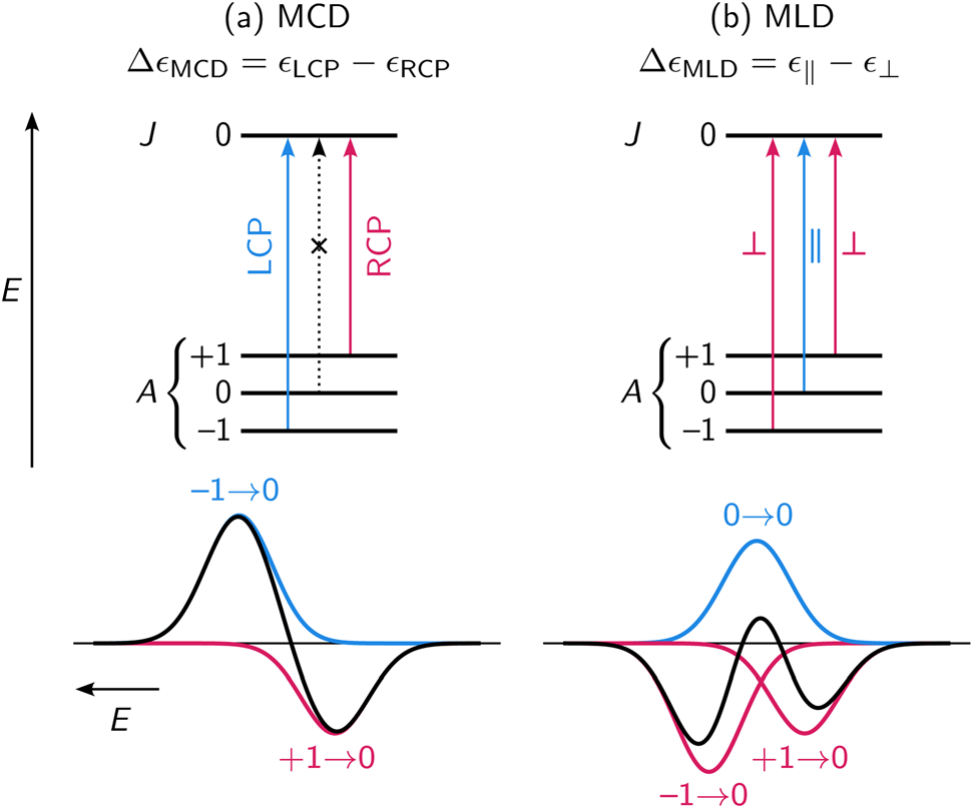
Observed (a) MCD and (b) MLD features for an *A* → *J* transition are typically modeled as sums of zeroth-, first-, and second-order derivative lineshapes.^24^ These lineshapes are caused by the close energetic spacing of Zeeman-split *M*_*J*_ levels, their uneven Boltzmann population, and their different interactions with polarized light, among other considerations (see SI Section S6.1).

The 
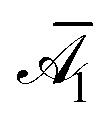
, 
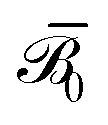
, and 
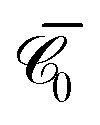
 constants are called “Faraday” parameters, and their signs can be inferred from symmetry in favorable point groups.^34^ It should be noted that the 
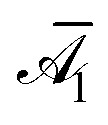
 term contributes derivative-shaped features to the spectrum, while 
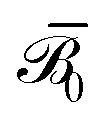
 and 
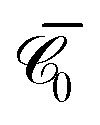
 terms contribute typical absorption-shaped features (Fig. 2a). The relative orders of magnitude of these parameters for an f–f transition are roughly^35^4
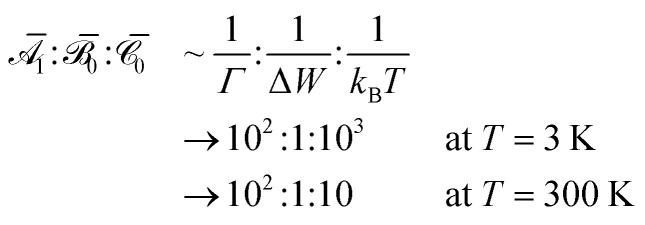
where we have used values typical of 1·Pr: bandwidth at half-maximum *Γ* = 50 cm^−1^ and energy differences between states Δ*W* = 2500 cm^−1^. It is always possible to use a weaker magnetic field in order to remain in a linear MCD response region, even at cryogenic temperatures.

Analogous Taylor expansion of eqn (2) gives no MLD intensity at first order. Instead, the first nonzero term is at second order in the applied field, yielding a quadratic MLD response,5
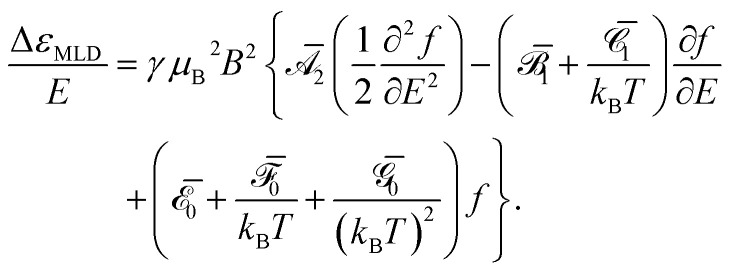


Eqn (5) shows that six Faraday parameters (
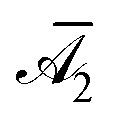
, 
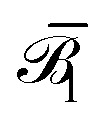
, 
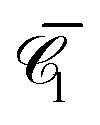
, 
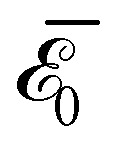
, 
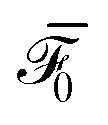
, and 
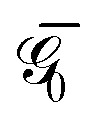
) are required to model non-saturating MLD intensity, and features can appear as zeroth-, first-, or second-order derivatives of the absorption lineshape (Fig. 2b).^35^ The relative orders of magnitude of these parameters for lanthanides generally vary as^40^6
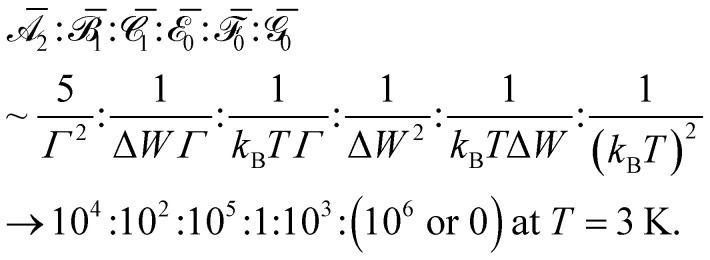


It has been shown that isolated doublets (or effective doublets) are unable to produce any 
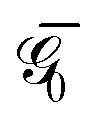
 MLD intensity,^25,41^ and this is consistent with our observations of 
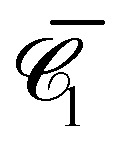
 as the dominant intensity pattern for 1·Pr (*vide infra*). As temperatures rise, any 
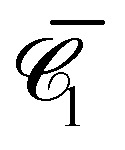
 and 
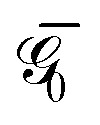
 MLD intensity should decrease as *T*^−1^ and *T*^−2^, respectively, meaning that 
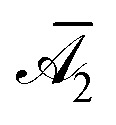
 features may dominate the MLD spectra of lanthanides under warmer conditions than those explored here.

Every transition within an MCD or MLD spectrum has its own unique set of Faraday parameters associated with it. These MCD and MLD Faraday parameters can be positive, zero, or negative, and their signs vary depending on the identities and symmetries (irreps) of the initial and final states involved in the transition. We have predicted the signs of MCD 
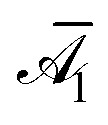
 and 
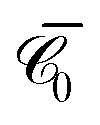
 signals and MLD 
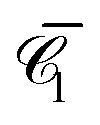
 signals for a generic 4f^2^ ion using the Wigner–Eckart theorem in the *D*_4d_ double group,^34,42^ and these signs are summarized in Table 1 (see SI Section S3 for derivation). Our prediction of Faraday parameter signs extends previous analyses of MCD 
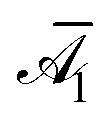
 intensity patterns that were developed using Judd–Ofelt theory.^17,43^ Judd–Ofelt theory is a common model of f–f intensity based on mixing of 4f^*N*^ states with 4f^*N*−1^5d^1^ and 4f^*N*−1^5g^1^ states, and its most well-known form fits absorptivities using only three parameters (*Ω*_2_, *Ω*_4_, *Ω*_6_).^28^ Several of its core assumptions, however, break down at cryogenic temperatures and for lanthanides with low-lying 4f^*N*−1^5d^1^ states (such as Pr^III^).^29^ Our predictions in Table 1 are thus more broadly applicable since they rely only on symmetry. Nonetheless, it is worth pointing out that deviations from these expectations can still occur if mixing between *M*_*J*_ levels appreciably alters the effective *g* values of the GS or ES away from the Landé *g* values, or if the geometry strays from ideal *D*_4d_ symmetry. Additionally, 
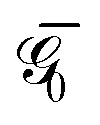
 intensity can begin to grow as the second-lowest CF level approaches the energy of the lowest level.

**Table 1:** Signs of MCD (
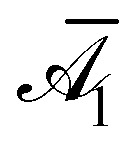
 and 
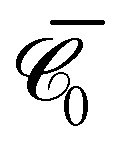
) and MLD (
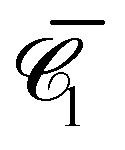
) Faraday parameters from the ^3^H_4_ ground state of Pr^III^ assuming perfect *D*_4d_ symmetry and ideal Landé *g* factors^a^

^2*S*+1^L_*J*_ ES	^3^H_4_ GS *M*_*J*_ and irrep *Γ*														
*M*_*J*_ *Γ*	0 A_1_^a^			±1 E_3_			±2 E_2_			±3 E_1_			±4^b^ B_1_ + B_2_		
	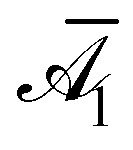	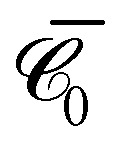	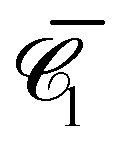	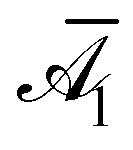	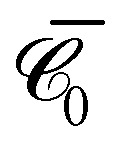	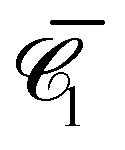	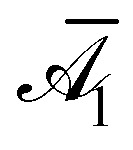	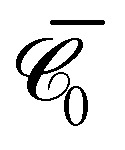	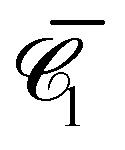	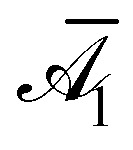	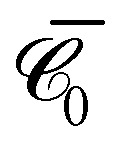	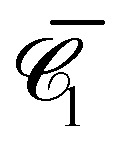	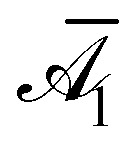	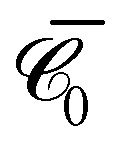	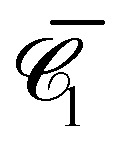
0 A_1,2_^a^	0	0	0	0	0	0	0	0	0	−	−	−	0	0	0
±1 E_3_	0	0	0	0	0	0	−	−	−	0	0	+	+	0	0
±2 E_2_	0	0	0	−	−	−	0	0	+	+	+	−	0	0	0
±3 E_1_	−	0	0	0	0	+	+	+	−	0	0	0	0	0	0
±4^c^ B_1_ + B_2_	0	0	0	+	+	−	0	0	0	0	0	0	0	0	0
±5 E_1_	+	0	0	0	0	−	−	+	+	0	0	0	0	0	0
±6 E_2_	0	0	0	+	−	+	0	0	−	−	+	+	0	0	0

a This table can be used to predict the signs of MCD or MLD Faraday coefficients for a given transition originating from the ^3^H_4_ ground state. For a given initial *M*_*J*_ or irrep (columns), the expected sign of Faraday coefficients for a transition to the final *M*_*J*_ or irrep (rows) is given. ^*a*^ The *M*_*J*_ = 0 levels transform as A_1_ for even *J* and A_2_ for odd *J*. ^*b*^ Treatment of GS *M*_*J*_ = ±4 levels was performed by assuming the B_1_ combination was lowest; the same results are obtained if B_2_ is lowest. ^*c*^ Treatment of ES *M*_*J*_ = ±4 assumed the splitting between B_1_ and B_2_ levels was less than the linewidth.

### 2.2 Electronic structure and molecular symmetry

Lanthanide electronic structure is usually modeled as a sum of atomic contributions and crystal field contributions: *Ĥ* = *Ĥ*_atom_ + *Ĥ*_CF_. We will describe each in turn.

The atomic Hamiltonian *Ĥ*_atom_ characterizes the energy levels of a 4f^*N*^ ion in the absence of any ligands, and it takes the form7
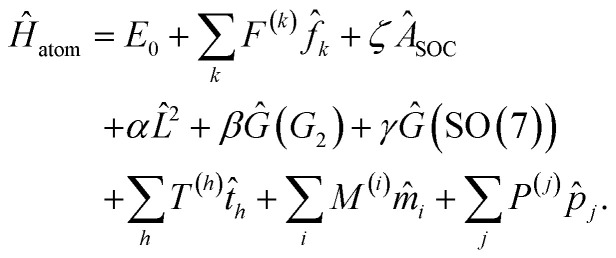


This equation parametrizes the effects of interelectronic repulsion (*F*^(*k*)^), SOC (*ζ*), configuration interaction (*α*, *β*, *γ*, *T*^(*h*)^), and more (see SI for more detailed definitions of the terms). The strongest of these effects is interelectronic repulsion. This interaction breaks the 4f^*N*^ levels into multiple states characterized by spin S and orbital L quantum numbers, also known as Russell–Saunders or LS coupling. Within the f block, SOC is the next strongest effect and it couples the spin and orbital angular momenta into a total angular momentum *J*. This splits each ^2*S*+1^L state into a series of ^2*S*+1^L_*J*_ states, which are shown for a free atomic Pr^III^ ion in Fig. 1b.

The introduction of a crystal field (CF) disrupts the spherical symmetry of an atom or atomic ion, lowering the system into one of the molecular point groups. This descent in symmetry lifts the degeneracy of the *M*_*J*_ levels within each ^2*S*+1^L_*J*_ state, and the resulting splitting is typically modeled using one-electron operators as8
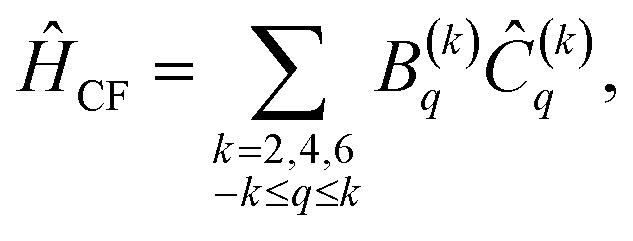
where *B*^(*k*)^_*q*_ are the CF parameters and *Ĉ*_*q*_(*k*) are the spherical tensor operators of rank *k* and component *q* (see SI Section S2.2). Here, we have used the Wybourne convention,^44^ but the reader should be aware that there are multiple equivalent conventions to describe f-block CF interactions.^45^ Even within a single convention, different choices of *xyz* axis orientations can lead to multiple equivalent sets of *B*^(*k*)^_*q*_ values, so care must be taken when making comparisons.

Eqn (8) appears to require 27 *B*^(*k*)^_*q*_ parameters, but many *B*^(*k*)^_*q*_ are necessarily zero due to molecular symmetry. Only *Ĉ*_*q*_(*k*) (or linear combinations thereof) that transform as the totally symmetric irrep of the point group may have nonzero *B*^(*k*)^_*q*_ parameters.^46^ There are only three valid (*k*, *q*) pairs in a *D*_4d_-symmetric system,^42^

9 *Ĥ*_CF_ = *B*^(2)^_0_*Ĉ*^(2)^_0_ + *B*^(4)^_0_*Ĉ*^(4)^_0_ + *B*^(6)^_0_*Ĉ*^(6)^_0_.

The pattern of *M*_*J*_ splitting caused by this *Ĥ*_CF_ perturbation can be straightforwardly predicted using the *D*_4d_ double group. For a 4f^2^ Pr^III^ ion in *D*_4d_ symmetry, its *M*_*J*_ levels are expected to split as shown in the leftmost column of Table 1; for example, the ^3^H_4_ GS should split into an A_1_ level (*M*_*J*_ = 0), an E_3_ level (degenerate *M*_*J*_ = ±1 pair), an E_2_ level (*M*_*J*_ = ±2), an E_1_ level (*M*_*J*_ = ±3), and a B_1_ + B_2_ level (quasi-degenerate *M*_*J*_ = ±4 pair). This process of mixing into levels that correspond to irreps of the double group generally causes *M*_*J*_ to cease to be a good quantum number; however, we have tried whenever possible to continue to associate *M*_*J*_ values to levels according to the largest component of the wavefunction.

**Fig. 3:**
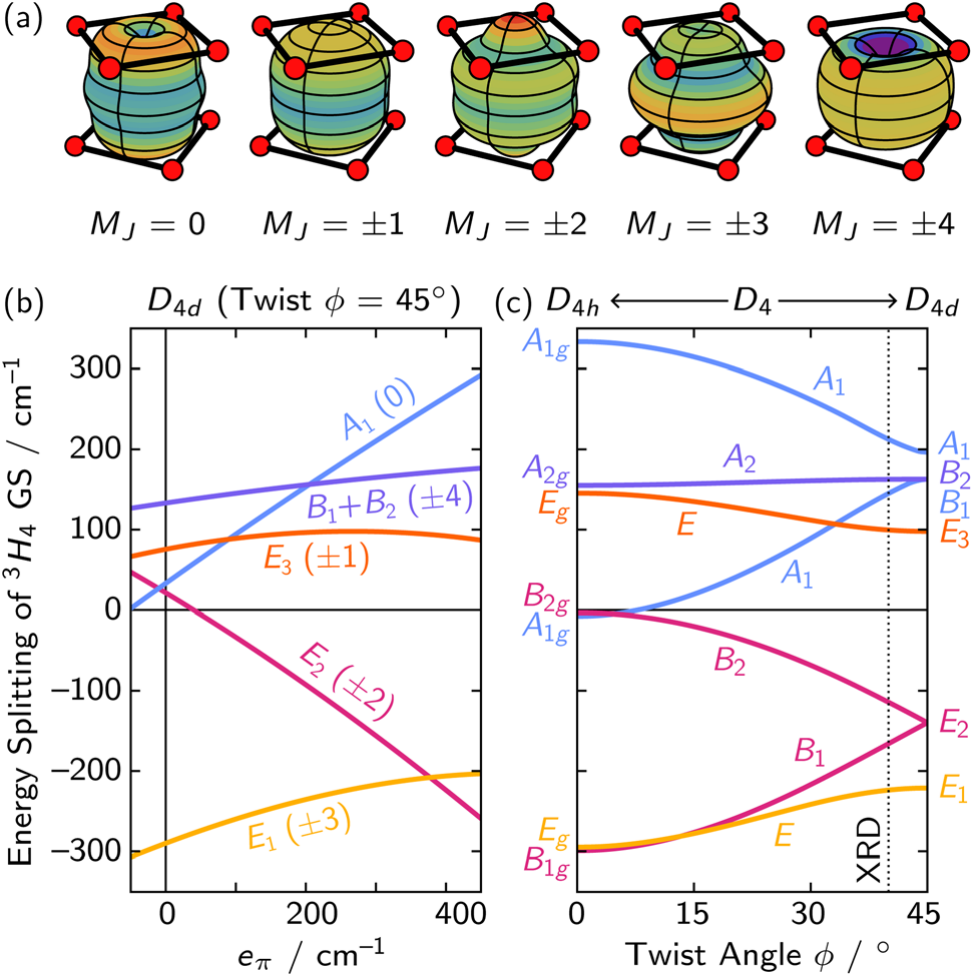
(a) Charge density distributions for the *M*_*J*_ levels within the ^3^H_4_ GS of Pr^III^ show the ±3 levels should be lowest in energy. (b) The predicted splitting from an AOM treatment of 1·Pr in perfect *D*_4d_ symmetry using *e*_σ_ = 400 cm^−1^. (c) Deviations from ideal *D*_4d_ symmetry (45° twist) cause descent into *D*_4_ symmetry. Notably, this splits the E_2_ level into a B_1_ and a B_2_ level (*e*_σ_ = 400 cm^−1^, *e*_π_ = 265 cm^−1^).

While the double group reveals the pattern of CF splitting, prediction of their energetic ordering requires a model. A popular heuristic uses charge density distributions of the *M*_*J*_ levels to predict how electrostatic repulsion of point-like ligands around the f ion will influence the energies.^47,48^ These distributions (Fig. 3a) suggest the ±3 levels of the ^3^H_4_ GS experience the least destabilization by the pseudo-*D*_4d_ CF of 1·Pr. More quantitative predictions were found using the angular overlap model (AOM) to estimate the CF splitting of the GS (Fig. 3b), which agreed that the E_1_ (*M*_*J*_ = ±3) levels are expected to lie lowest in energy for ligands with typical π interactions (*e*_π_ < *e*_σ_). Twisting of the two polyoxometallate ligands away from the ideal 45° angle causes a *D*_4d_ ⊃ *D*_4_ descent in symmetry and further splits the levels (Fig. 3c), so accurate assignment of MCD and MLD spectra can help to determine the average symmetry of a species in solution.

## 3 Results and discussion

### 3.1 Synthesis and room temperature spectroscopy

We began our exploration of joint lanthanide MCD/MLD spectroscopy through the selection of 1·Pr as a useful target compound. Polyoxometalates have been extensively used as ligands in lanthanide coordination chemistry for a variety of applications,^49^ and precise control over their CF interactions have led to magnetic hysteresis, spin–electric coupling, molecular clock transitions, and more.^4,50,51^ The 1·Ln family of compounds is known for most of the lanthanide series (Ln = La, Ce, Nd, Sm–Er), and crystallographic studies have shown the anions to adopt roughly *D*_4d_-symmetric geometries with twist angles ranging from 38.2(2)–40.4(2)°.^52^ We have prepared 1·Pr for the first time, and its structure revealed a similar twist angle of 40.7(2)° between the two POM ligands (Fig. 1a).

**Fig. 4:**
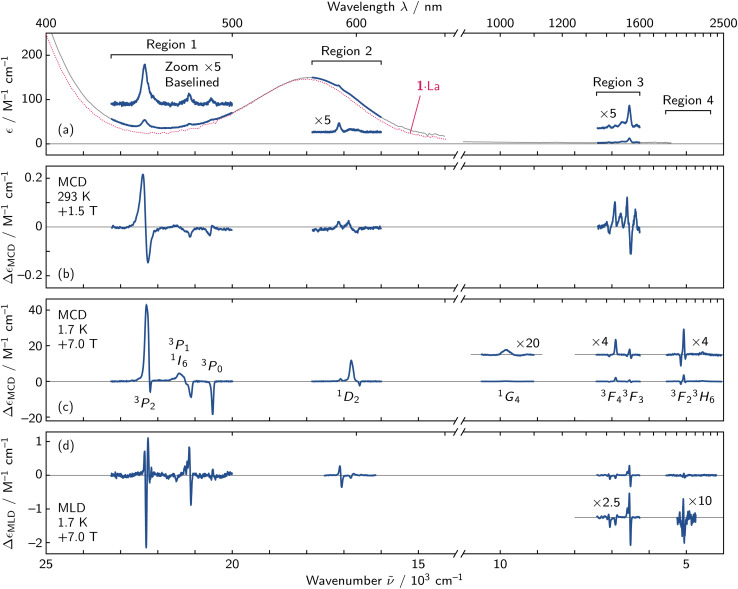
(a) Absorption, (b) and (c) MCD, and (d) MLD spectra of 1·Pr are shown at various temperatures. Regions of interest are shown in blue and labeled, and an absorption spectrum of 1·La is included for comparison. There are no data within region 4 in the room temperature spectra due to the strong absorption of the solvent.

Our studies of the electronic structure of 1·Pr began by collecting the room temperature UV-vis-NIR absorption spectrum over the 400–1850 nm wavelength region (Fig. 4a). Solutions of 1·Pr in 9 : 1 methanol-*d*_4_/ethanol-*d*_6_ were bright purple in color due to the presence of a broad feature at 550 nm (*ε* = 150 M^−1^ cm^−1^) that dominated the visible absorption spectrum.^52^ This transition is generally understood to arise from a d_*xz*,*yz*_ → d_*xy*_ excitation within the {MoNO}^4^ moiety of the polyoxometalate ligand, and it can be seen across the entire family of previously reported 1·Ln compounds (*cf.*1·La in Fig. 4a).^52–54^

Several weaker f–f transitions were anticipated on top of the strong ligand-centered transition based on comparison with the atomic ion (Fig. 1b) and with a Dieke diagram.^55^ Zooming into the spectrum revealed clusters of transitions that appear in three regions (Fig. 4a), and these clusters can be coarsely assigned as ^3^H_4_ → ^3^P_0,1,2_ + ^1^I_6_ in region 1, ^3^H_4_ → ^1^D_2_ in region 2, and ^3^H_4_ → ^3^F_3,4_ in region 3. The MCD spectrum over the same regions (Fig. 4b) offered a distinct advantage over the absorption spectrum in locating the f–f features because MCD intensity is largely driven through SOC.^17,35,36^ This means that the weaker f–f transitions of the paramagnetic Pr^III^ ion show enhanced MCD over those localized within the diamagnetic polyoxometalate ligand.

Together, our room temperature studies gave evidence for observation of seven of the eight expected ESs of Pr^III^ in the 400–1850 nm wavelength range. The limited solubility of 1·Pr in 9 : 1 methanol/ethanol prevented location of the ^1^G_4_ transition expected near 1000 nm, even using a saturated solution in a 4 cm path length cuvette. The search for further f–f transitions at energies higher than 25 000 cm^−1^ (*λ* < 400 nm) and lower than 5400 cm^−1^ (*λ* > 1850 nm) was prevented by the strong absorption from the polyoxometalate ligand and the solvent, respectively.

### 3.2 Cryogenic magneto-optical spectroscopies

While approximate energies of transitions could be identified from room temperature spectra, detailed insight required collection of cryogenic magneto-optical data. Additionally, the 0.12 cm path length of our cryogenic sample holder allowed collection of data out to 2400 nm (4200 cm^−1^), revealing another cluster of transitions in a fourth region (region 4), corresponding to the ^3^F_2_ and ^3^H_6_ states (Fig. 4c).

Inspection of the cryogenic MCD and MLD spectra showed several obvious differences from the room temperature data (Fig. 4c and d). The MCD spectrum at 1.7 K appeared sharpened, strengthened, and simplified due to the 1/*k*_B_*T* variation of 
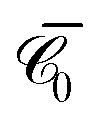
 MCD intensity (Eq. (3)); thus, 
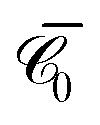
 intensity alone dominated over any temperature-independent 
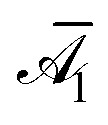
 term intensity. No vibronic progressions were observed in the transitions, and the low temperature ensured that there were no hot bands in the spectrum from population of low-lying vibrational or electronic excited states. It is fascinating to compare the MCD spectrum with the MLD spectrum, which is dominated by derivative-shaped 
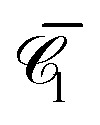
 term features. The sharp MLD features give increased precision in peak position over the MCD spectrum, and the simultaneous fitting of MCD and MLD data greatly assisted in deconvoluting overlapped transitions.

Analysis of the MCD and MLD spectra of 1·Pr required determination of *Γ*_i_, the irrep of the lowest CF level within the ^3^H_4_ GS, and we approached this through inspection of the ^3^H_4_ → ^3^P_0_ transition located in region 1 (20 527.6 cm^−1^). Selection of a transition to a *J* = 0 state like ^3^P_0_ was convenient because there cannot be any complications from CF splitting of the ES. The MCD associated with this transition had strongly negative 
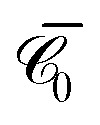
 intensity at low temperatures and revealed a negative 
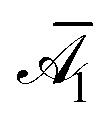
 intensity after warming to 80 K (SI Fig. S6a). Additionally, the MLD intensity was best modeled with both negative 
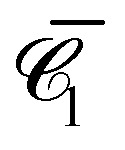
 and 
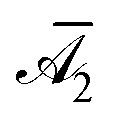
 Faraday parameters (SI Fig. S6c). The strong intensity of the MCD feature and the uniformly negative MCD and MLD Faraday parameters (Table 1) are consistent with this feature arising from an E_1_ → A_1_ transition. This interpretation agrees with the AOM prediction of an E_1_ (*M*_*J*_ = ±3) level lowest (Fig. 3b).

**Fig. 5:**
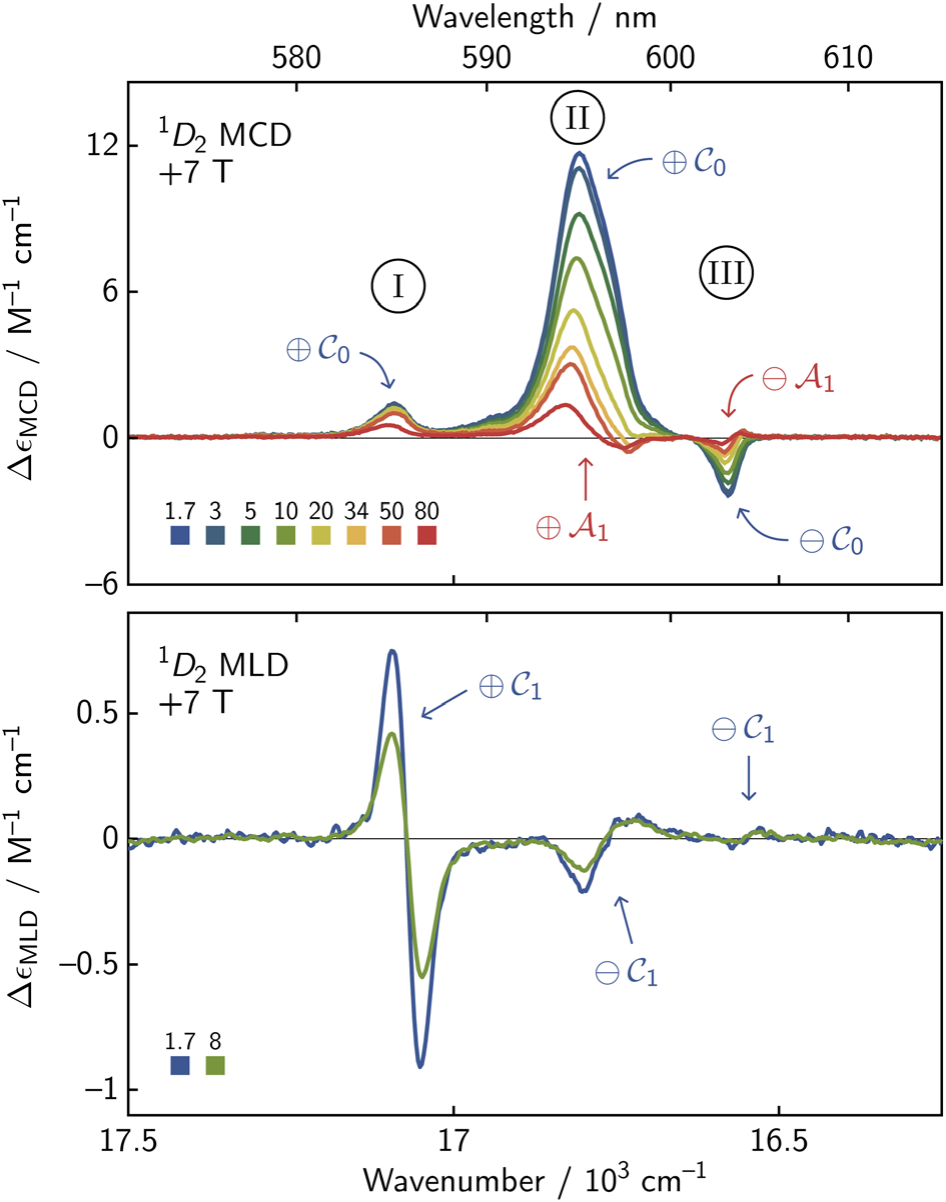
The MCD and MLD spectra of the three ^3^H_4_ →^1^D_2_ features in region 2 are labeled with Roman numerals I, II, and III. These features together allow for confident assignment of each transition through comparison of the 
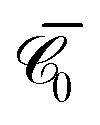
, 
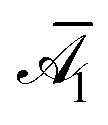
, and 
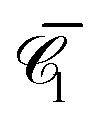
 intensities.

Identification of an E_1_ (*M*_*J*_ = ±3) ^3^H_4_ GS enabled the assignment of the remainder of the features. We will demonstrate by focusing on region 2, which shows ^3^H_4_ →^1^D_2_ transitions that we have labeled with Roman numerals in Fig. 5. A *D*_4d_ CF will split the ^1^D_2_ state into an A_1_ (*M*_*J*_ = 0) level, an E_3_ (*M*_*J*_ = ±1) level, and an E_2_ (*M*_*J*_ = ±2) level according to Table 1, and this trifurcation is seemingly confirmed by the observation of three transitions: I (17 085 cm^−1^), II (16 975 cm^−1^), and III (16 576 cm^−1^). These transitions display two positive (I, II) and one negative (III) MCD 
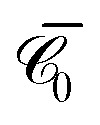
 term features at 1.7 K. Warming the sample to 80 K revealed negative and positive 
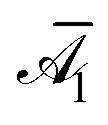
 intensities associated with transitions I and II, respectively. The negative 
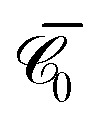
 and 
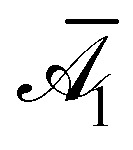
 MCD intensities associated with transition III clearly implicate an A_1_ level; however, the Table 1 suggests that only one positive 
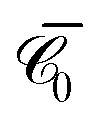
 MCD feature should be observed. It may thus be suspected that the small feature for transition I could be due to the MCD-forbidden E_1_ → E_3_ transition, its intensity coming from some slight deviation from *D*_4d_ symmetry. MCD sign and lineshape unfortunately give no further insight. Here, the utility of simultaneous MLD analysis comes into relief. Transition II with positive MCD 
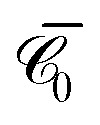
 and 
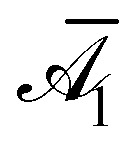
 intensity has negative MLD 
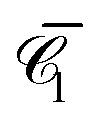
 intensity; whereas, the weaker MCD 
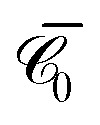
 feature of transition I has strong positive MLD 
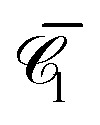
 intensity. These patterns of MCD and MLD intensities demand that transition II is assigned to the ^1^D_2_ E_2_ (*M*_*J*_ = ±2) level, and transition I is indeed due to the ^1^D_2_ E_3_ (*M*_*J*_ = ±1) level. Explanations of the assignments in other regions (Table 2) follow similar logic and can be found in the SI (Section S4.1). Satisfyingly, our assignments were found to match in MCD 
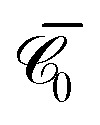
 sign to those from CASSCF(2,7)/RI-NEVPT2 calculations using ORCA 6.0.1 (ref. 56–62) (SI Section S5).

**Table 2:** Comparison between experimental energies (cm^−1^) of 1·Pr 4f^2^ levels and calculated values derived from the *D*_4d_ fitted parameters

Level *Γ* (*M*_*J*_)		*E*_exp_	*E*_calc_	Δ*E*
^1^S_0_	A_1_ (0)	—	47 301.2	—
^3^P_2_	E_3_ (±1)	22 328.0	22 332.7	+4.7
	E_2_ (±2)	22 285.0	22 255.9	−29.1
	A_1_ (0)	22 222.9	22 261.1	+38.2
^1^I_6_	B_1_ + B_2_ (±4)	—	22 052.7	—
	E_1_ (±5)	—	21 978.1	—
	E_1_ (±3)	—	21 845.1	—
	E_2_ (±2)	—	21 604.8	—
	E_3_ (±1)	—	21 431.6	—
	E_2_ (±6)	—	21 399.0	—
	A_1_ (0)	—	21 369.2	—
^3^P_1_	E_3_ (±1)	21 140.7	21 121.2	−19.5
	A_2_ (0)	—	21 084.3	—
^3^P_0_	A_1_ (0)	20 527.6	20 534.1	+6.5
^1^D_2_	E_3_ (±1)	17 084.9	17 074.9	−10.0
	E_2_ (±2)	16 794.8	16 788.3	−6.5
	A_1_ (0)	16 575.6	16 590.0	+14.4
^1^G_4_	A_1_ (0)	—	10 221.1	—
	B_1_ + B_2_ (±4)	—	10 097.1	—
	E_3_ (±1)	—	10 047.7	—
	E_2_ (±2)	—	9731.9	—
	E_1_ (±3)	—	9703.4	—
^3^F_4_	A_1_ (0)	7136.3	7140.8	+4.5
	B_1_ + B_2_ (±4)	—	7086.5	—
	E_3_ (±1)	7073.2	7067.9	−5.3
	E_2_ (±2)	6894.6	6879.3	−15.3
	E_1_ (±3)	—	6849.9	—
^3^F_3_	A_2_ (0)	6581.3	6552.2	−29.1
	E_3_ (±1)	6520.2	6501.9	−18.3
	E_2_ (±2)	6480.7	6497.3	+16.6
	E_1_ (±3)	—	6486.9	—
^3^F_2_	A_1_ (0)	5171.2	5178.6	+7.4
	E_2_ (±2)	5092.4	5119.0	+26.6
	E_3_ (±1)	5090.5	5088.2	−2.3
^3^H_6_	E_2_ (±6)	4894.1	4886.0	−8.1
	A_1_ (0)	—	4662.8	—
	E_3_ (±1)	—	4652.4	—
	E_2_ (±2)	4587.0	4599.8	+12.8
	E_1_ (±3)	—	4486.6	—
	E_1_ (±5)	—	4421.4	—
	B_1_ + B_2_ (±4)	—	4358.5	—
^3^H_5_	E_1_ (±5)	—	2544.9	—
	A_2_ (0)	—	2475.9	—
	E_3_ (±1)	—	2417.8	—
	E_2_ (±2)	—	2336.7	—
	B_1_ + B_2_ (±4)	—	2225.2	—
	E_1_ (±3)	—	2216.5	—
^3^H_4_	B_1_ + B_2_ (±4)	—	422.7	—
	A_1_ (0)	—	371.9	—
	E_3_ (±1)	—	323.9	—
	E_2_ (±2)	—	142.8	—
	E_1_ (±3)	0	−5.2	−5.2

When performing this analysis, we want to emphasize caution in interpretation of MCD/MLD signs in the presence of saturation because MCD and MLD features are able to vary in both strength and sign as a function of field and temperature. If analyzing a cryogenic MCD spectrum collected at strong field, it is crucial to ensure that the sign of the MCD feature is the same at weak fields. For our MCD analysis, we have generally done so by estimating the derivative of MCD intensity with respect to field at zero field, (∂Δ*ε*_MCD_/∂*B*)|_*B*=0_. For our MLD analysis, it was not as easy to estimate second derivatives at weak field, so we have collected spectra at a large number of weak and intermediate field strengths to ensure no flips in sign were apparent.

### 3.3 Crystal field splittings

The determination of the identities of these transitions allowed us to fit the electronic structure of 1·Pr to a *D*_4d_ Hamiltonian *Ĥ* = *Ĥ*_atom_ + *Ĥ*_CF_ using eqn (7) and (9). Fitting was done in a stepwise manner, gradually adding increasing numbers of off-diagonal matrix elements to the Hamiltonian (see SI Section S4.3) until we arrived at the parameters listed in Table 3. Comparison between the experimental and calculated values in Table 2 shows close agreement, generally falling within a few dozen wavenumbers of the experimental value. Most notably, our fitting procedure yields entirely experimentally-determined wavefunctions for the states of 1·Pr. This is a significant achievement, as the wavefunctions are rich with information about the system that is valuable in the understanding and optimization of optical and magnetic properties. It is also worth emphasizing that these wavefunctions were obtained without need for computational methods like DFT or multireference calculations.

**Table 3:** Fitted parameters from *D*_4d_ model^a^

Parameter	Atomic^b^	1·Pr
*E*_avg_	10 201(40)	10 109(13)
*F* ^(2)^	71 761(211)	68 819(180)
*F* ^(4)^	51 721(558)	50 926(503)
*F* ^(6)^	33 675(629)	33 930(289)
*α*	24.04(5)	27.4(1.3)
*β*	−626(5)	−873(65)
*γ*	1476(238)	1343^c^
*ζ*	763.025(266)	744(9)
*M* ^(0)^	1.663(61)	0.0(1.6)^d^
*P* ^(2)^	235(6)	409(149)^d^
*B* ^(2)^_0_		−109(38)
*B* ^(4)^_0_		−1927(111)
*B* ^(6)^_0_		424(287)

a All values in cm^−1^ and parentheses show one standard uncertainty in the final digits.

b Values were converted from the orthogonal convention in ref. 31 using definitions in ref. 63.

c Fixed to the indicated value taken from aqueous Pr(iii).^64^

d Ratios were fixed using the values from Carnall, which were informed from Hartree–Fock calculations: *M*^(0)^ : *M*^(2)^ : *M*^(4)^ was taken as 1 : 0.56 : 0.31 and *P*^(2)^ : *P*^(4)^ : *P*^(6)^ was taken as 1 : 0.5 : 0.1.^65^

Our best-fit parameters also provided useful information about the molecular geometry in solution. The AOM was used to correlate the *B*^(*k*)^_*q*_ CF parameters with metal–ligand interaction strengths and the geometry of the ligands about the metal.^66–68^ If we assume cylindrically symmetric π-type interactions for each Pr–O bond, a *D*_4d_ arrangement gives the following equations for *B*^(*k*)^_*q*_ parameters:10
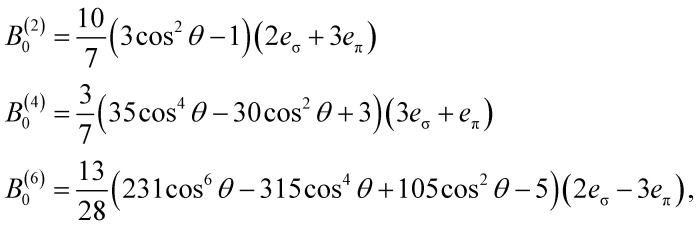
where *θ* is the angle from the fourfold (*z*) axis to the O atoms of the POM ligands, *e*_σ_ is the strength of the *σ*-type interaction, and *e*_π_ is the individual strength of each π-type interaction. Using an average angle of *θ* = 55° from the crystal structure allows fitting of *e*_σ_ = 421(28) cm^−1^ and *e*_π_ = 198(48) cm^−1^. When the angle *θ* is allowed to vary, it is found that the best-fit *B*^(*k*)^_*q*_ values correspond to an angle of *θ* = 55.8(4)° and strengths of *e*_σ_ = 431(28) cm^−1^ and *e*_π_ = 211(44) cm^−1^. The closeness between this fitted *θ* value and the crystallographic one shows that 1·Pr retains its pseudo-*D*_4d_ geometry in solution.

### 3.4 Magnetic saturation behavior

A natural question arises whether 1·Pr should truly be treated as *D*_4d_ in symmetry, or whether its twisting angle causes appreciable changes in electronic structure. Variable temperature variable field (VTVH) MCD analysis gives us insight. A truly *D*_4d_ system with its E_1_ (*M*_*J*_ = ±3) levels lowest should experience magnetic saturation with an effective *g* value of *g*_eff_ = 3× (4/5) = 12/5 (or 2.4), where 4/5 is the Landé *g* value for the ^3^H_4_ GS. Structural distortions away from ideal symmetry will cause deviations in the observed *g* value, and fitting a saturation curve to the 
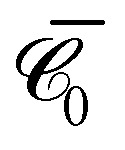
 intensity of the ^3^P_0_ at 1.7 K shows a decreased value of *g*_eff_ = 1.79(5). Clearly, the average molecule of 1·Pr in solution has some degree of distortion. There are many ways that the molecule could distort in solution, but the most obvious distortion from the crystal structure is a change in the twist angle *ϕ* between POM ligands. Twisting to the angle in the structure (*ϕ* = 40°) causes a *D*_4d_ ⊃ *D*_4_ descent in symmetry, mixing the *M*_*J*_ = ±3 levels with the *M*_*J*_ = ∓1 levels and lowering the *g*_eff_ value. This twisting also splits the low-lying E_2_ state into 
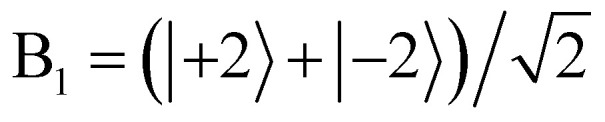
 and 
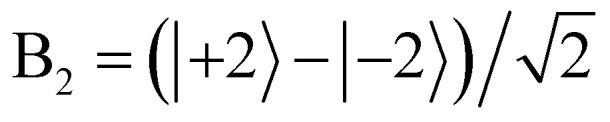
 states (Fig. 3c).

**Fig. 6:**
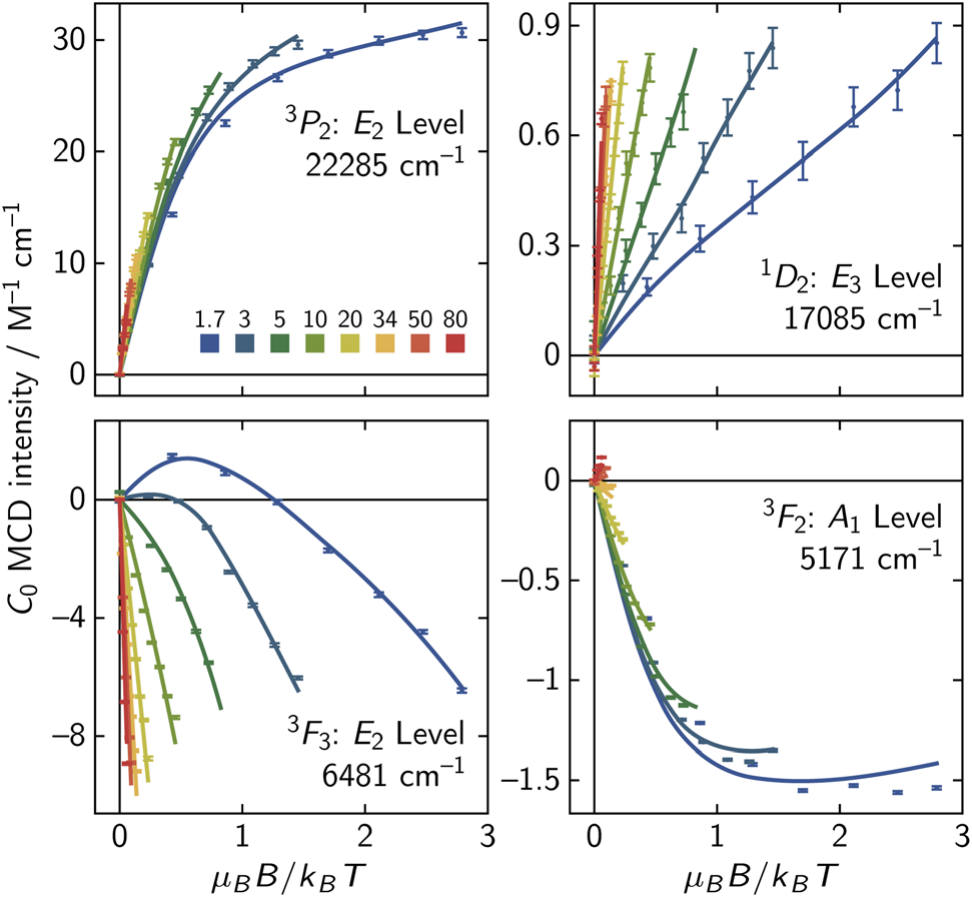
Nesting of the VTVH MCD saturation curves indicate the presence of a low-lying ES. The best-fit curves from an effective spin Hamiltonian fitting procedure are indicated, and they correspond to a low-lying ES at 92(4) K.

The VTVH MCD data show nesting, symptomatic of a low-lying ES (Fig. 6). We were able to model the VTVH MCD saturation curves through construction of an effective spin Hamiltonian,^36–39^ giving *g*_eff_ = 2.34(3) and the presence of the low-lying |B_1_〉 ES at *E* = 92(4) cm^−1^. This *g*_eff_ value could be obtained through introduction of *B*^(4)^_±4_ and *B*^(6)^_±4_ terms in eqn (9) (see SI Section S2.2) and would correspond to a twist angle of 37(2)° according to the AOM (Fig. 3c). At this twisting angle, the GS would be 97% |*M*_*J*_ = ±3〉 in composition, suggesting the *D*_4d_ CF model is adequate for modeling the ES CF splitting of 1·Pr. This angle is also remarkably similar to the one in the crystal structure, again underscoring the power of the AOM in providing geometric insight into f-block elements in solution.

**Fig. 7:**
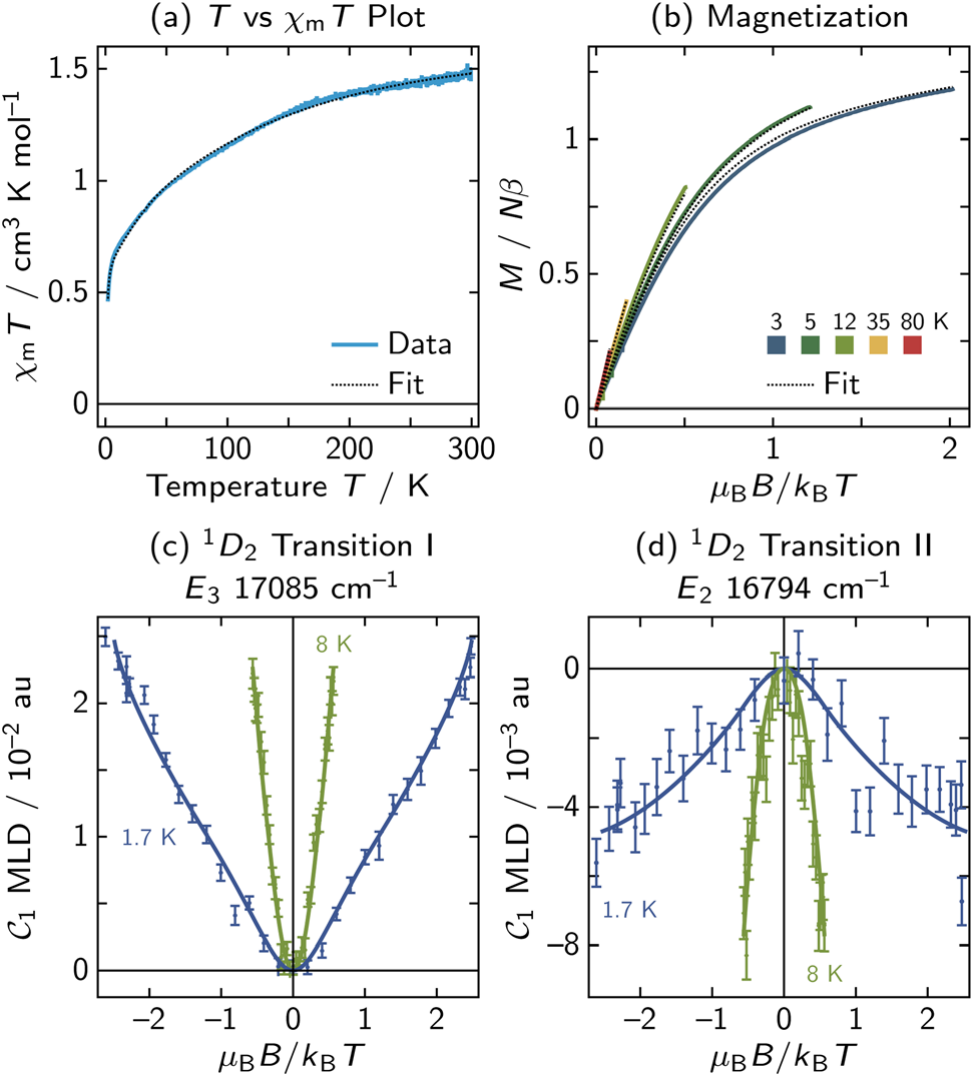
Vibrating sample magnetometry was used to measure the (a) *T vs. χ*_m_*T* and (b) *B vs. M* response of 1·Pr. Fitting these curves gave slightly different CF parameters than fitting the ES CF splittings. Preliminary VTVH saturation curves of 
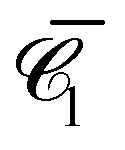
 MLD intensity for the ^3^H_4_ →^1^D_2_ feature are also shown for two of its transitions (c) and (d).

It is interesting to note that a different set of CF parameters is obtained by fitting *T vs. χ*_m_*T* and *B vs. M* data from vibrating sample magnetometry (VSM) of 1·Pr (Fig. 7a and b) than those obtained through fitting of ES splittings from MCD/MLD spectra. Fitting the VSM data using EasySpin^69^ gave *B*^(2)^_0_ = −393, *B*^(4)^_0_ = −1373, *B*^(6)^_0_ = −1154, *B*^(4)^_±4_ = −344, and *B*^(6)^_±4_ = −1054 cm^−1^. This set of *B*^(*k*)^_0_ CF parameters is similar in sign and magnitude to those in Table 3, but it deviates more than three standard uncertainties from the ES MCD/MLD fit. The differences between GS and ES fits highlight an important albeit inconvenient fact of f-block CF splittings: CF parameters are known to vary from state to state.^12–15^ If one is interested in the ES photophysical properties of a molecule, joint MCD/MLD analysis of ES splittings can be expected to provide more useful insight into CF interactions. If instead one is interested in the magnetic response of a molecule, GS magnetometry through VTVH MCD saturation curves, VSM, or SQuID (superconducting quantum interference device) measurements are likely to be more relevant.

Despite the differences between ES and GS fits, the two GS magnetometry techniques agree closely: the VSM CF parameters predict the lowest-lying excited state to be at *E* = 89 cm^−1^, which is within one standard uncertainty of our effective Hamiltonian treatment of VTVH MCD saturation curves. More accurate insight would have to come from higher resolution techniques than magnetometry, such as far-infrared magnetospectroscopy or high-field electron paramagnetic resonance spectroscopy. For any interested readers, we also include two plots of VTVH MLD data for the ^3^H_4_ →^1^D_2_ transition (Fig. 7c and d). We have not incorporated these data in our fitting routines, but VTVH MLD measurements clearly hold promise for future studies of MLD magnetometry.

## 4 Conclusions

This work has demonstrated the power of UV-vis-NIR MLD spectroscopy as a complement to MCD spectroscopy for studying f-block electronic structure. Together, these two magneto-optical techniques have provided conclusive assignments of much of the fine structure observed for 1·Pr and allowed fitting of the transition energies with the Hamiltonian *Ĥ* = *Ĥ*_atom_ + *Ĥ*_CF_. Our fit gave detailed structural information about the geometry of 1·Pr in solution and, perhaps most excitingly, yielded wavefunctions derived strictly from experimental observables. Because such 4f^*N*^ wavefunctions determine the molecular optical and magnetic properties, experimentally derived wavefunctions are rich with information that is valuable in future tuning of f-block molecular materials and nanomaterials.^70^

We have also highlighted the deep relation between molecular symmetry and these magneto-optical spectroscopies. Symmetry is known to control many desirable properties like circularly polarized luminescence (CPL),^71^ magnetic CPL,^34,72^ magnetochiral dichroism,^73^ spin–electric coupling,^74^ and ultranarrow optical transitions;^75^ thus, the utility of symmetry-based insight into electronic structure from joint MCD–MLD analysis is difficult to overstate. Our lab is continuing to explore the implementation of MLD spectroscopy in the understanding of f-block complexes, both moving downwards into the actinides and rightwards into ions with higher f^*N*^ counts, especially Kramers systems. Many research groups with MCD spectroscopy instrumentation may already be equipped to acquire MLD spectra, and we hope this work encourages broader adoption of this information-dense technique.

## Author contributions

WJT conceived the project. IER and KO synthesized 1·Pr, and KO developed its final preparative route. GA and XG collected vibrating sample magnetometry data. SMG collected most of the MCD, MLD, and absorption spectra. WJT and SMG performed all analysis and wrote the manuscript.

## Conflicts of interest

There are no conflicts of interest to declare.

## Supplementary Material

SC-OLF-D5SC05890B-s001

SC-OLF-D5SC05890B-s002

SC-OLF-D5SC05890B-s003

## Data Availability

The absorption, MCD, and MLD data supporting this article have been included as part of the supplementary information (SI). Supplementary information is available. See DOI: https://doi.org/10.1039/d5sc05890b. CCDC 2457433 contains the supplementary crystallographic data for this paper.^76^
