# Supplementary material for: UV-vis-NIR magnetic linear dichroism: a powerful complement to MCD for f-block electronic structure
Source: Chem Sci. 2025 Nov 27;17(4):2199–209. doi: 10.1039/d5sc05890b (PMC12679488; doi:10.1039/d5sc05890b)
Supplement: SC-017-D5SC05890B-s002 [file SC-017-D5SC05890B-s002.pdf]

Supplementary Information (SI) for Chemical Science.
This journal is © The Royal Society of Chemistry 2025

UV–Vis–NIR Magnetic Linear Dichroism: A Powerful Complement to MCD for f Block Electronic Structure

Sydney M. Giles, Kevin O'Neil, Ian E. Ramsier, Wesley J. Transue

Supporting Information

Contents

S1 General Information **2**
S1.1 Synthesis of $[n\text{-Bu}_4\text{N}]_3[\text{Pr}\{\text{Mo}_5\text{O}_{13}(\text{OMe})_4(\text{NO})\}_2]$ (**1**·Pr) . . . 2
S1.2 Crystal Structure of **1**·Pr . . . 2
S1.3 Acquisition of UV–Vis–NIR Absorption, MCD, and MLD Data . . . 5

S2 Electronic Structure of a Lanthanide Ion **7**
S2.1 Atomic Hamiltonian . . . 7
S2.2 Crystal Field (CF) Hamiltonian and the Angular Overlap Model . . . 8
S2.3 Construction of Symmetry-Adapted Wavefunctions . . . 9

S3 Theoretical Treatment of MCD/MLD Signs and Saturation Curves **12**
S3.1 Transition Dipole Moments and the Wigner–Eckart Theorem . . . 12
S3.2 MCD/MLD Intensity Expressions and Orientational Averaging . . . 13
S3.3 MCD Saturation Curve of an Effective Doublet with $g_\perp = 0$ . . . 18

S4 MCD and MLD Spectra and Analysis **19**
S4.1 Full Data Set and Assignments . . . 19
S4.1.1 Region 1 . . . 21
S4.1.2 Region 2 . . . 22
S4.1.3 Region 3 . . . 22
S4.1.4 Region 4 . . . 23
S4.2 Calculation of Saturation Curves . . . 24
S4.3 Crystal Field Fitting . . . 26
S4.4 Comparison with Vibrating Sample Magnetometry . . . 26

S5 CASSCF/NEVPT2 Multireference Calculations **27**
S5.1 Calculated Energy Levels and MCD Signs . . . 27
S5.2 XYZ Coordinates . . . 30

S6 Appendices **34**
S6.1 First/Second Derivative Lineshapes from the BO–FC–RS Approximation . . . 34
S6.2 Taylor Series Expansion of Intensity Equations . . . 34

S1 General Information

Except where otherwise noted, all manipulations were performed on the benchtop or in the fume hood open to air. Hydroxylamine hydrochloride (Fisher Chemical), N,N'-dicyclohexylcarbodiimide (DCC, Thermo Scientific), 1-ethyl-3-(3-dimethylaminopropyl)carbodiimide hydrochloride (EDC·HCl, Thermo Scientific), praseodymium nitrate pentahydrate (Thermo Scientific), anhydrous methanol (Sure Seal), methanol-d_4 (Thermo Scientific), and ethanol-d_6 (Thermo Scientific) were purchased and used as received. Literature compounds $[n\text{-Bu}_4\text{N}]_4[\alpha\text{-Mo}_8\text{O}_{26}]$ [1] and $[n\text{-Bu}_4\text{N}]_3[\text{La}\{\text{Mo}_5\text{O}_{13}(\text{OMe})_4(\text{NO})\}_2]$ (**1**·La) [2] were prepared according to the reported procedures. High resolution mass spectrometry (HRMS) was obtained on a Thermo Scientific Q-Exactive mass spectrometer through electrospray ionization (ESI); the mobile phase was acetonitrile spiked with formic acid.

S1.1 Synthesis of $[\text{n-Bu}_4\text{N}]_3[\text{Pr}\{\text{Mo}_5\text{O}_{13}(\text{OMe})_4(\text{NO})\}_2]$ (1·Pr)

We found that **1**·Pr could be prepared following the literature method from She et al.; [2] however, we found the resulting material to be contaminated with impurities and N,N'-dicyclohexylurea (DCU). Related compound with mixed POM–DCU ligands are known, [3] and this may have contributed to our separation challenges. Fractional recrystallization provided cleaner **1**·Pr but spectroscopically pure material ultimately required manual sorting of crystals. We have found that substitution of N,N'-dicyclohexylcarbodiimide (DCC) for 1-ethyl-3-(3-dimethylaminopropyl)carbodiimide hydrochloride (EDC·HCl) provided **1**·Pr with fewer contaminants. Our yield of **1**·Pr (17%) was somewhat reduced from those reported by She et al. (22–61% varying by lanthanide), [2] but we found purification and isolation in our preparation below to be easier.

A 10 mL round bottom flask was charged with $[n\text{-Bu}_4\text{N}]_4[\alpha\text{-Mo}_8\text{O}_{26}]$ (828 mg, 0.385 mmol, 1.25 equiv), hydroxylamine hydrochloride (25.6 mg, 0.369 mmol, 1.2 equiv), EDC·HCl (140 mg, 0.677 mmol, 2.2 equiv), and praseodymium(III) nitrate pentahydrate (134 mg, 0.308 mmol, 1 equiv). A reflux condenser was attached and the assembly was flushed with argon. Anhydrous methanol (5 mL) was added and the resulting yellow slurry was refluxed (70 °C) for 3 h with stirring. In this time the mixture first darkened to a brown color, then lightened to a final purple hue with undissolved yellow solids. The mixture was cooled to room temperature and the condenser was removed. The workup of the reaction mixture was performed open to air. The flask was cooled in an ice bath, precipitating yellow solids that were filtered from the purple supernatant and discarded. This process of cooling in an ice bath and discarding yellow solids was repeated until no further precipitation was observed. The purple solution was dried under vacuum, and the resulting purple residue was washed with water (5 × 5 mL) to remove EDC and its byproducts. Recrystallizing by slow evaporation of a methanol solution yielded 134.4 mg (17%) bright purple crystals. HRMS (ESI, MeCN) m/z: Calc'd for $\{[n\text{-BuN}_4]_2[\text{Pr}\{\text{Mo}_5\text{O}_{13}(\text{OMe})_4(\text{NO})\}_2]\}^-$ ($\text{C}_{40}\text{H}_{96}\text{Mo}_{10}\text{N}_4\text{O}_{36}\text{Pr}^-$) 2308.5472; Found 2308.5395. ATR-IR (major peaks, cm^{-1}): 686, 839, 936, 1033, 1056, 1627, 2815, 2959. UV–Vis–NIR (λ nm (ϵ M^{-1} cm^{-1})): 550 (150).

S1.2 Crystal Structure of 1·Pr

The crystals were mounted in hydrocarbon oil on a nylon loop or a glass fiber. Low-temperature (200 K) data were collected on a Bruker D8 Venture Duo diffractometer coupled to a Photon III CPAD with Mo Kα radiaton ($\lambda = 0.71073$ Å) with ϕ- and ω-scans. Data were indexed with Apex II (Difference Vectors method), integrated with Bruker SAINT, and a semi-empirical absorption correction was applied using SADABS. The space group was established using XPREP, and the structure was solved by intrinsic phasing methods using SHELXT-2018/2 and refined against F^2 on all data by full-matrix least squares with SHELXL-2019/2 using established methods. All non-hydrogen atoms were refined anisotropically. All hydrogen atoms were included in the model at geometrically calculated positions and refined using a riding model unless otherwise noted. The isotropic displacement parameters of all hydrogen atoms were fixed to 1.2 times the U_{eq} value of the atoms they are linked to (1.5 times for methyl groups).

Compound **1**·Pr crystallized in the triclinic space group $P\bar{1}$ with one entire molecule and one molecule of methanol in the asymmetric unit. The structure showed disorder in several of the methoxide groups, the tetrabutylammonium cations, and the methanol solvent of crystallization. These disorders were refined with the help of similarity restraints on 1–2 and 1–3 distances and displacement parameters as well as rigid bond restraints for anisotropic displacement parameters.

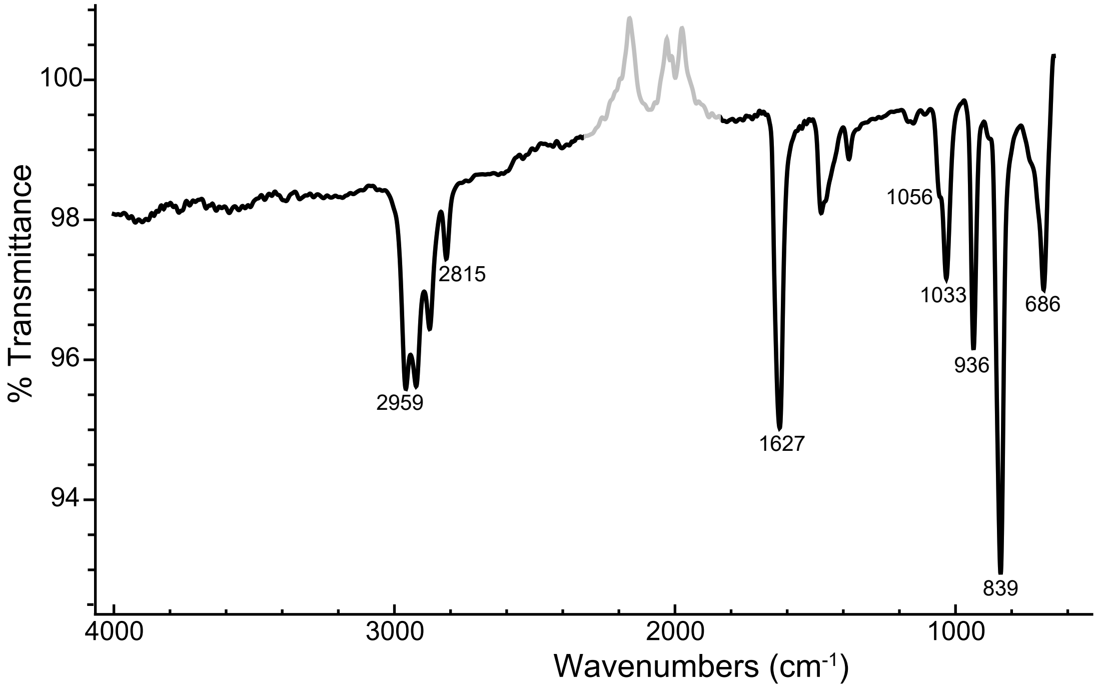

Figure S1: ATR-IR spectrum of **1**·Pr is in close agreement with the IR spectra measured by She et al.[2] of multiple other **1**·Ln and is characteristic of the POM ligand. The peak at 686 cm^{-1} is assigned to vibrations in the Mo–O–Mo bonds. The peaks at 936 and 839 cm^{-1} are assigned to the vibration of the terminal Mo=O bonds. The peak at 1033 cm^{-1} is assigned to the C—O stretch within the OMe groups. The peak at 1627 is assigned to the NO vibration. The vibrations in the 2800–3000 cm^{-1} range are from C–H vibrations in the OMe units. The grayed portion of the spectrum is from baseline correction errors from atmospheric gases. Despite the appearance of methanol in the crystal structure of **1**·Pr, no O–H stretch was observed. This is consistent with the spectra reported by She et al..

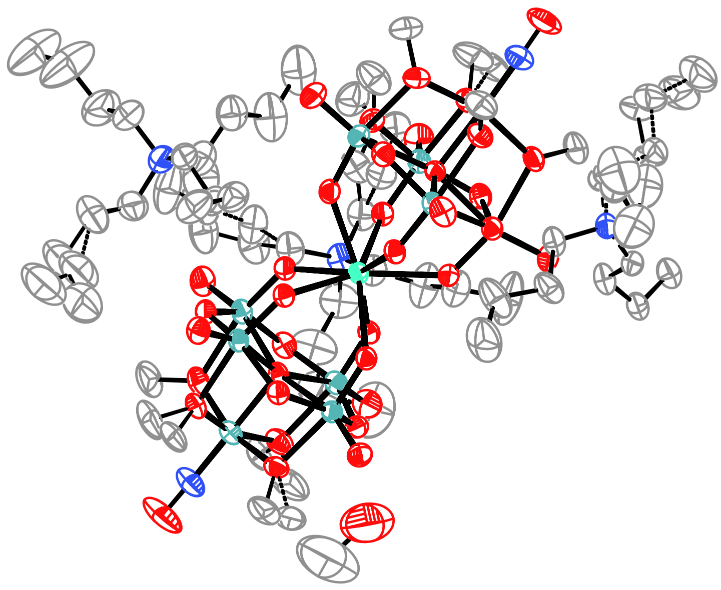

Figure S2: Thermal ellipsoid (50%) plot for the asymmetric unit of the crystal structure of [n-$Bu_4N]_3[Pr\{Mo_5O_{13}(OMe)_4(NO)\}_2]$·MeOH. Hydrogen atoms were omitted for clarity.

Table S1: Summary of crystallographic data

	$[n\text{-Bu}_4N]_3[Pr\{Mo_5O_{13}(OMe)_4(NO)\}_2]\cdot MeOH$
CCDC	CSD 2457433
Empirical formula	$C_{28.5}H_{68}Mo_5N_{2.5}O_{18.5}Pr_{0.5}$
Formula weight (g/mol)	1292.00
Color / Morphology	purple / needle
Crystal size (mm^3)	0.26 × 0.06 × 0.04
Temperature (K)	200(2)
Wavelength (Å)	0.71073
Crystal system, Space group	Triclinic, $P\bar{1}$
Unit cell dimensions (Å, °)	$a = 12.7347(5)$, $\alpha = 97.665(2)$<br>$b = 17.5024(9)$, $\beta = 95.943(2)$<br>$c = 22.5850(11)$, $\gamma = 110.063(2)$
Volume ($Å^3$)	4625.5(4)
Z	4
Density (calc., g/cm^3)	1.855
Absorption coefficient (mm^{-1})	1.900
$F(000)$	2576
Theta range for data collection (°)	2.082 to 29.154
Index ranges	$-17 \leq h \leq 17$, $-23 \leq k \leq 23$,<br>$-30 \leq l \leq 30$
Reflections collected	289707
Independent reflections, R_{int}	24880, 0.0622
Completeness to $\theta_{\max}$ (%)	99.9
Absorption correction	Multi-Scan
Refinement method	Full-matrix least squares on F^2
Data / Restraints / Parameters	24880 / 2298 / 1183
Goodness-of-fit[a]	1.134
Final R indices[b] $[I > 2\sigma(I)]$	$R_1 = 0.0369$, $wR_2 = 0.0888$
R indices[b] (all data)	$R_1 = 0.0500$, $wR_2 = 0.0989$
Largest diff. peak and hole ($e\cdot Å^{-3}$)	1.793 and −0.942

[a] Goodness-of-fit $= \sqrt{\frac{\sum[w(F_o^2-F_c^2)^2]}{(n-p)}}$ [b] $R_1 = \frac{\sum||F_o|-|F_c||}{\sum|F_o|}$; $wR_2 = \sqrt{\frac{\sum[w(F_o^2-F_c^2)^2]}{\sum[w(F_o^2)^2]}}$; $w = \frac{1}{\sigma^2(F_o^2)+(aP)^2+bP}$; $P = \frac{2F_c^2+\max(F_o^2,0)}{3}$

S1.3 Acquisition of UV–Vis–NIR Absorption, MCD, and MLD Data

Absorption, magnetic circular dichroism (MCD), and magnetic linear dichroism (MLD) spectroscopies were performed using a JASCO J-1700 spectropolarimeter equipped with a S-20 photocathode-equipped photomultiplier tube (PMT) detector for the UV–visible region ($\lambda = 163-950$ nm, JASCO Model PM-539), or one of two InGaAs detectors for the visible–near infrared (NIR) region ($\lambda = 800-1600$ nm EXIG-542 or $\lambda = 1600-2500$ nm EXIG-543). All samples were solutions of the analyte in 9:1 methanol-d_4/ethanol-d_6. The solvent system was selected based on a few considerations: solubility of the analyte, quality of glass to avoid scattering, and minimization of solvent peaks in spectral windows of interest.

Room temperature (293 K) MCD spectra were acquired using a JASCO MCD-581 electromagnet (≤1.5 T) at a series of field strengths (e.g. 0, ±1, ±1.5 T). These experimental conditions are in the linear limit ($\mu_B B/k_B T \ll 1$), giving MCD intensity proportional to the applied field. Under these linear conditions, the field-independent and field-dependent components of the measured ellipticity data were separated using a Moore–Penrose pseudoinverse. For example, collection of five spectra over the 300–800 nm region at $B = 0, \pm1, \pm1.5$ T field strengths allows extraction of the baseline (field-independent $I^{(0)}$) and MCD (field-dependent $I^{(1)}$) components through

$$\begin{pmatrix} 1 & 1.5 \\ 1 & 1 \\ 1 & 0 \\ 1 & -1 \\ 1 & -1.5 \end{pmatrix}^{\ominus} \begin{pmatrix} I^{+1.5\text{ T}}_{300\text{ nm}} & I^{+1.5\text{ T}}_{301\text{ nm}} & I^{+1.5\text{ T}}_{302\text{ nm}} & \cdots & I^{+1.5\text{ T}}_{800\text{ nm}} \\ I^{+1.0\text{ T}}_{300\text{ nm}} & I^{+1.0\text{ T}}_{301\text{ nm}} & I^{+1.0\text{ T}}_{302\text{ nm}} & \cdots & I^{+1.0\text{ T}}_{800\text{ nm}} \\ I^{0.0\text{ T}}_{300\text{ nm}} & I^{0.0\text{ T}}_{301\text{ nm}} & I^{0.0\text{ T}}_{302\text{ nm}} & \cdots & I^{0.0\text{ T}}_{800\text{ nm}} \\ I^{-1.0\text{ T}}_{300\text{ nm}} & I^{-1.0\text{ T}}_{301\text{ nm}} & I^{-1.0\text{ T}}_{302\text{ nm}} & \cdots & I^{-1.0\text{ T}}_{800\text{ nm}} \\ I^{-1.5\text{ T}}_{300\text{ nm}} & I^{-1.5\text{ T}}_{301\text{ nm}} & I^{-1.5\text{ T}}_{302\text{ nm}} & \cdots & I^{-1.5\text{ T}}_{800\text{ nm}} \end{pmatrix} = \begin{pmatrix} I^{(0)}_{300\text{ nm}} & I^{(0)}_{301\text{ nm}} & I^{(0)}_{302\text{ nm}} & \cdots & I^{(0)}_{800\text{ nm}} \\ I^{(1)}_{300\text{ nm}} & I^{(1)}_{301\text{ nm}} & I^{(1)}_{302\text{ nm}} & \cdots & I^{(1)}_{800\text{ nm}} \end{pmatrix},$$

where "$\ominus$" indicates the pseudoinverse. The first column of ones in the leftmost matrix indicates that the baseline is constant among the data sets, and the second column of the leftmost matrix contains the strengths of the applied fields. The output gives the field-independent ellipticity in its first row (i.e. the baseline), and the field-dependent ellipticity in its second row (i.e. MCD expressed in millidegrees per tesla). The field-dependent ellipticity $[\theta]$ was converted to $\Delta\epsilon$ MCD intensity by $\Delta\epsilon = [\theta\,(\text{mdeg})]/(32982Cl)$ using concentration C and path length l.

Cryogenic MCD spectra were acquired using an Oxford SpectromagPT superconducting magnet ($T = 1.7-300$ K, $|B| \leq 7$ T) with four Suprasil windows. Samples were contained between two quartz windows (Spectrocell NIR quartz, 1 cm diameter) and a fluoropolymer O-ring, then flash-frozen in a liquid nitrogen bath to obtain an optically transparent glass, and loaded into the magnet sample compartment cold. Depolarization was checked using a nickel(II) tartrate solution before/after the sample to ensure less than 5% loss in CD intensity. The JASCO J-1700 spectropolarimeter was used by separating the detector and positioning it on the far side of the magnet, taking care to ensure the detector was positioned outside the 50 G line. A series of three planoconvex CaF_2 lenses were used to focus the light from the spectrometer through the sample and to the detector. The spectrometer was positioned on the south side of the magnet and the detector on the north side so that the direction of propagation of light was collinear with the direction of the applied magnetic field.

Cryogenic MCD data were collected over five regions of the UV–vis–NIR range. All MCD spectra were acquired at negative fields, and the resulting spectra have been appropriately inverted to correspond to positive-field spectra. Data were acquired using parameters (scanning speed, detector integration time, number of accumulations, and bandwidth) chosen to maximize the signal-to-noise ratio while preventing distortion of features. As discussed in the J-1700 manual, the product of the detector integration time and the scanning speed limits the resolution of peaks that may be discerned and should be chosen to be less than 1/10 the peak width. Spectra in Regions 1 (430–500 nm) and 2 (560–625 nm) used a 0.1 nm data pitch, 0.125 s detector integration time, 0.10 nm bandwidth, and a 10 nm/min scanning speed. Spectra in Region 3 (1350–1600 nm) used a 0.2 nm data pitch, 0.5 s detector integration time, 10 nm bandwidth, and a 200 nm/min scanning speed. Spectra in Region 4 (1800–2400 nm) used a 0.2 nm data pitch, 0.5 s detector integration time, 10 nm bandwidth, and a 500 nm/min scanning speed. Spectra in Regions 1–4 were collected at each point shown in the grid in Fig. S3, giving 58 spectra per region. Another sample with a thicker path length (0.2 vs 0.12 cm) was used to search for the weaker $^1G_4$ transition in a fifth region (950–1100 nm) using a 0.1 nm data pitch, 1 s detector integration time, 30 nm bandwidth, and a 100 nm/min scanning speed. This wider bandwidth let more light through the sample, but also broadened the appearance of the

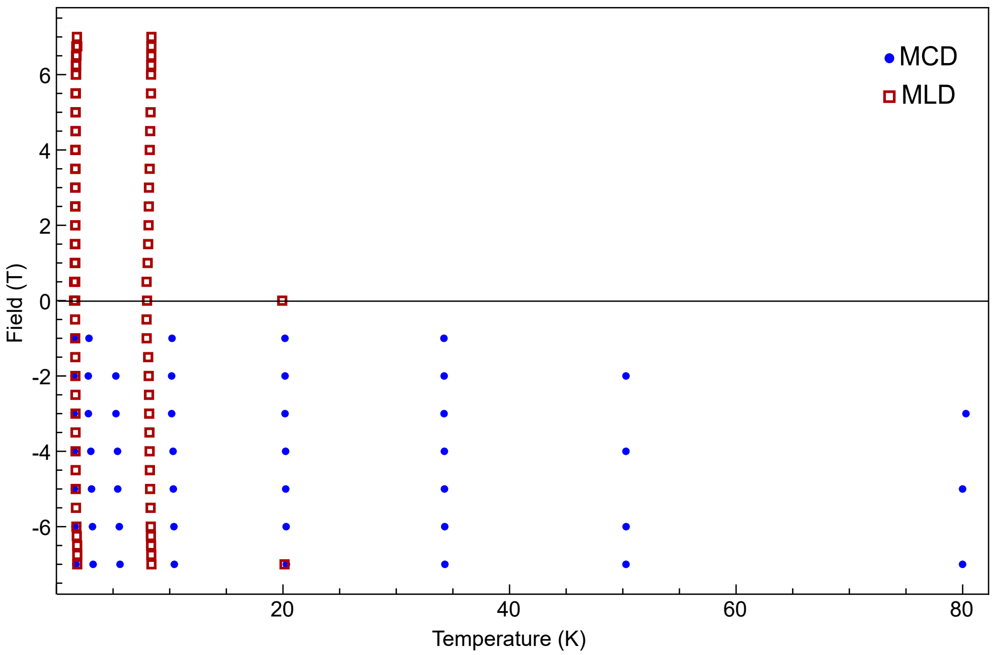

Figure S3: Fields and temperatures at which MCD (blue circle) and MLD (dark red square) spectra were collected.

feature that was located.

The JASCO J-1700 has an uncertainty associated with the wavelength that the manual reports to be ±0.1 nm over 163–250 nm, ±0.2 nm over 250–500 nm, ±0.5 nm over 500–800 nm, ±1.5 nm over 800–1200 nm, ±2.0 nm over 1200–1600 nm, and ±5.0 nm over 1600–2500 nm ranges. This is not typically a problem for broad charge transfer or d–d transitions, but efforts had to be taken to align the x axes of spectra for the sharp f–f transitions observed here. Within each region, one transition was chosen for the purposes of alignment: the $^3P_0$ transition around 20 530 cm^{-1} in Region 1, the $^1D_2$ transition around 16 570 cm^{-1} in Region 2, the $^3F_4$ transition around 6890 cm^{-1} in Region 3, and the $^3F_2$ transition around 5090 cm^{-1} in Region 4. For each of these transitions, lineshape fitting was used to estimate each the offset of each spectrum from the average. The maximum deviation from the average was found to be 0.13 nm in Region 1, 0.35 nm in Region 2, 1.24 nm in Region 3, and 1.75 nm in Region 4 — all within the uncertainties inherent to the instrument. After aligning the x axes, spectra were denoised using a singular value decomposition treatment: only singular values that did not correspond to noise were retained (three in Region 1, two in Region 2, three in Region 3, two in Region 4, and two in the $^1G_4$ region).

Cryogenic MLD data were collected over Regions 1–4 using identical parameters to the MCD data acquisitions. The experimental setup differed in the orientation of the spectrometer and detector, which were arranged so that light propagated perpendicular to the applied magnetic field. Data in Regions 1 and 2 were collected using the PMT detector and the PEM operating at 100 kHz. Data in Regions 3 and 4 were collected using the InGaAs detectors, but these detectors do not respond quickly enough to run the PEM at 100 kHz. Instead, an additional quarter wave plate was introduced to convert the circularly polarized NIR light into linearly polarized light. Depolarization was checked using the JASCO LD standard (45° quartz plate) positioned before/after the sample to ensure less than 5% loss in signal. MLD data were collected at a series of positive and negative fields (Fig. S3) to ensure that any spurious MCD response could be subtracted.[4]

S2 Electronic Structure of a Lanthanide Ion

S2.1 Atomic Hamiltonian

The atomic Hamiltonian most commonly used for an f^N ion takes the form

$$\hat{H}_{\text{atom}} = E_0 + \sum_{k=2,4,6} F^{(k)}\hat{f}_k + \zeta\hat{A}_{\text{SOC}} + \alpha\hat{L}^2 + \beta\hat{G}(G_2) + \gamma\hat{G}(SO(7)) + \sum_{\substack{h=2,3,4,\\6,7,8}} T^{(h)}\hat{t}_h + \sum_{i=0,2,4} M^{(i)}\hat{m}_i + \sum_{j=2,4,6} P^{(j)}\hat{p}_j. \tag{S1}$$

This expression allows for parametrization of several influences on the f^N manifold.

The influence of interelectron repulsion is modeled by the $\sum_{k=2,4,6} F^{(k)}\hat{f}_k$ term, where $F^{(k)}$ are scalar Slater–Condon integrals and $\hat{f}_k$ are two-electron operators,[5]

$$\hat{f}_k = \sum_{i>j}\sum_{q=-k}^{k} (-1)^q \hat{C}^{(k)}_{-q}(i)\hat{C}^{(k)}_{q}(j), \tag{S2}$$

where the first sum is over all unique pairs of electrons (i, j) in the $4f^2$ manifold. Here, the $\hat{C}$ operators are one-electron spherical tensor operators that can be evaluated using the usual $SO(3)$ Wigner–Eckart theorem in the Racah convention,

$$\langle jm|\hat{T}^{(k)}_q|j'm'\rangle = (-1)^{j-m}\begin{pmatrix} j & k & j' \\ -m & q & m' \end{pmatrix}\langle j\|\hat{T}^{(k)}\|j'\rangle, \tag{S3}$$

where $\left(\begin{smallmatrix} \cdot & \cdot & \cdot \\ \cdot & \cdot & \cdot \end{smallmatrix}\right)$ is a $3j$ symbol and $\langle j\|\hat{T}^{(k)}\|j'\rangle$ is a proportionality constant called a 'reduced matrix element.' This allows evaluation as[6]

$$\langle lm|\hat{C}^{(k)}_q|l'm'\rangle = (-1)^{l-m}\begin{pmatrix} l & k & l' \\ -m & q & m' \end{pmatrix}\langle l\|\hat{C}^{(k)}\|l'\rangle \tag{S4}$$

$$\langle l\|\hat{C}^{(k)}\|l'\rangle = (-1)^{l}\sqrt{(2l+1)(2l'+1)}\begin{pmatrix} l & k & l' \\ 0 & 0 & 0 \end{pmatrix} \tag{S5}$$

Because both $4f^2$ electrons are in f orbitals, we can use $l = l' = 3$.

Spin–orbit coupling (SOC) is introduced through the $\zeta\hat{A}_{\text{SOC}}$ operator. Here, ζ is the SOC constant and $\hat{A}_{\text{SOC}}$ is an effective one-electron central-field operator,

$$\hat{A}_{\text{SOC}} = \sum_i \hat{\vec{l}}(i)\cdot\hat{\vec{s}}(i). \tag{S6}$$

Note that this operator is different from the $\hat{\vec{L}}\cdot\hat{\vec{S}}$ SOC operator, which only provides intrastate SOC splitting.

Configuration interaction appears in two places in the atomic Hamiltonian. The three parameters α, β, and γ capture the influence of configuration interaction that comes at second order, and they use the traditional Racah[7] chain-of-groups description of states using non-invariance (non-symmetry)[8] Lie groups: $U(14) \supset Sp(14) \supset SU(2)^S \otimes (SO(7) \supset G_2 \supset SO(3))^L$.[7,9] The $\hat{L}^2$, $\hat{G}(G_2)$, and $\hat{G}(SO(7))$ are the Casimir operators* of $SO(3)$, G_2, and $SO(7)$. The meaning and consequence of these Lie groups and their Casimir operators is not important for the present discussion; we can simply express the operators as

$$\hat{G}(G_2) = \frac{1}{4}\sum_{k=1,5}\sum_{q=-k}^{k}(2k+1)(-1)^q\hat{U}^{(k)}_q\hat{U}^{(k)}_{-q} \tag{S7}$$

$$\hat{G}(SO(7)) = \frac{1}{5}\sum_{k=1,3,5}\sum_{q=-k}^{k}(2k+1)(-1)^q\hat{U}^{(k)}_q\hat{U}^{(k)}_{-q} \tag{S8}$$

*Or proportional to the usual Casimir operator in the case of $\hat{L}^2$

where $\hat{U}_q^{(k)}$ are the unit tensor operators ($\hat{U}_q^{(k)} = \sum_i \hat{u}_q^{(k)}(i)$, $\langle nl\|u^{(k)}\|n'l'\rangle = \delta_{nn'}\delta_{ll'}$). Configuration interaction also appears through the $\sum_{h=2,3,4,6,7,8} T^{(h)}\hat{t}_h$ term, which captures further shifts in state energy levels using three-electron operators. Because the $\hat{t}_h$ operators are effective three-electron operators, they cannot contribute to a $4f^2$ ion like $\mathrm{Pr^{III}}$, so we can set $T^{(h)} = 0$ for all h.

The influence of spin–spin (SS), spin–other-orbit (SOO), and electrostatically correlated spin–orbit coupling are introduced through the $\sum_i M^{(i)}\hat{m}_i$ and $\sum_j P^{(j)}\hat{p}_j$ terms.[10] The operators $\hat{m}_i$ can be calculated by collecting coefficients of $M^{(i)}$ in $\hat{H}_{\mathrm{SS}} + \hat{H}_{\mathrm{SOO}}$, where[10]

$$\hat{H}_{\mathrm{SS}} = -2\sum_{i\neq j}\sum_k \sqrt{(k+1)(k+2)(2k+3)}M^{(k)}\langle l\|\hat{C}^{(k)}\|l\rangle\langle l\|\hat{C}^{(k+2)}\|l\rangle\{\hat{w}^{(1,k)}(i)\hat{w}^{(1,k+2)}(j)\}^{(22)0} \tag{S9}$$

$$\begin{aligned}\hat{H}_{\mathrm{SOO}} = \sum_{i\neq j}\sum_k \sqrt{(k+1)(2l+k+2)(2l-k)}\times \\ \Big[\Big(M^{(k-1)}\langle l\|\hat{C}^{(k+1)}\|l\rangle^2 + 2M^{(k)}\langle l\|\hat{C}^{(k)}\|l\rangle^2\Big)\{\hat{w}^{(0,k+1)}(i)\hat{w}^{(1,k)}(j)\}^{(11)0} \\ + \Big(M^{(k)}\langle l\|\hat{C}^{(k)}\|l\rangle^2 + 2M^{(k-1)}\langle l\|\hat{C}^{(k+1)}\|l\rangle^2\Big)\{\hat{w}^{(0,k)}(i)\hat{w}^{(1,k+1)}(j)\}^{(11)0}\Big].\end{aligned} \tag{S10}$$

Here, $\hat{w}^{(\kappa,k)}(i)$ are double tensors for electron i of rank κ in spin space and k in orbital space, and they have reduced matrix elements $\langle l\|w^{(\kappa,k)}\|l\rangle = \sqrt{(2\kappa+1)(2k+1)}$. The electrostatically correlated operators can be calculated from the reduced matrix elements listed in Reference 10.

E_0 is an arbitrary value that shifts all energies up and down. For a $4f^2$ ion like $\mathrm{Pr^{III}}$, it is related to the average energy of the $4f^2$ manifold as $E_{\mathrm{avg}} = E_0 + \frac{288}{13}\alpha + \frac{12}{13}\beta + \frac{72}{65}\gamma$. Our fitting procedure (Sec. S4.3) allows E_{avg} to float to fit the lowest level of the $^3H_4$ GS to 0 cm^{-1}.

S2.2 Crystal Field (CF) Hamiltonian and the Angular Overlap Model

A Hamiltonian must always transform as the totally symmetric representation of the point group; thus, only $\hat{C}_q^{(k)}$ operators that transform according to this irrep may appear in the $\hat{H}_{\mathrm{CF}}$.[6] There are two point groups of interest used in the main manuscript, D_{4d} for a perfect 45° twist angle and D_4 when the twist angle deviates. The symmetrized operators that transform totally symmetrically in each of these point groups are[11]

$$D_{4d}\text{:}\quad \hat{C}_0^{(0)}, \hat{C}_0^{(2)}, \hat{C}_0^{(4)}, \hat{C}_4^{(5)} - \hat{C}_{-4}^{(5)}, \hat{C}_0^{(6)}, \hat{C}_4^{(7)} - \hat{C}_{-4}^{(7)}, \hat{C}_0^{(8)}, \hat{C}_8^{(8)} + \hat{C}_{-8}^{(8)}, \hat{C}_4^{(9)} - \hat{C}_{-4}^{(9)}, \ldots \tag{S11}$$

$$D_4\text{:}\quad \text{All from } D_{4d} \text{ plus } \hat{C}_4^{(4)} + \hat{C}_{-4}^{(4)}, \hat{C}_4^{(6)} + \hat{C}_{-4}^{(6)}, \hat{C}_4^{(8)} + \hat{C}_{-4}^{(8)}, \hat{C}_8^{(9)} - \hat{C}_{-8}^{(9)}, \ldots \tag{S12}$$

and there are infinitely many of these. The triangle rule limits the number of $\hat{C}_q^{(k)}$ operators that are relevant between two f orbitals to those with even values of k with $0 \leq k \leq 6$. For any f^N ion, the one-electron CF Hamiltonian in each of these point groups can thus be expressed as

$$\hat{H}_{\mathrm{CF}} = \sum_{k=2,4,6}\sum_{q=-k}^{k} B_q^{(k)}\hat{C}_q^{(k)} \tag{S13}$$

$$\xrightarrow{D_{4d}} B_0^{(2)}\hat{C}_0^{(2)} + B_0^{(4)}\hat{C}_0^{(4)} + B_0^{(6)}\hat{C}_0^{(6)} \tag{S14}$$

$$\xrightarrow{D_4} B_0^{(2)}\hat{C}_0^{(2)} + B_0^{(4)}\hat{C}_0^{(4)} + B_0^{(6)}\hat{C}_0^{(6)} + B_{\pm4}^{(4)}\left(\hat{C}_4^{(4)} + \hat{C}_{-4}^{(4)}\right) + B_{\pm4}^{(6)}\left(\hat{C}_4^{(6)} + \hat{C}_{-4}^{(6)}\right) \tag{S15}$$

so there are three CF parameters in D_{4d} and five in D_4 symmetry. The $B_0^{(0)}\hat{C}_0^{(0)}$ term has been omitted in both expressions because it does not influence splitting and its effect can be absorbed by E_{avg} (E_0).

The angular overlap model (AOM), closely related to the superposition model, can be used to estimate $B_q^{(k)}$ values by parametrizing the strength of σ-type and π-type interactions between the metal and ligands.[12] We will model the metal–ligand interactions of π symmetry as cylindrically symmetric (i.e. $\pi_x = \pi_y$) such that we need only two interaction strengths: e_σ for the energy of the σ interaction and e_π for the energy of each of the two π interactions. If we assume there are two POM ligands in a $D_{4(d)}$ geometry, the angular

orientation of the POM ligands can be described using an azimuthal angle θ from the z (C_4) axis and a twist (polar) angle ϕ ($\phi = 0°$ is eclipsed D_{4h} and $\phi = 45°$ is staggered D_{4d}). Expressions for the $B_q^{(k)}$ parameters are

$$B_0^{(2)} = \frac{10}{7}(3\cos^2\theta - 1)(2e_\sigma + 3e_\pi) \tag{S16}$$

$$B_0^{(4)} = \frac{3}{7}(35\cos^4\theta - 30\cos^2\theta + 3)(3e_\sigma + e_\pi) \tag{S17}$$

$$B_0^{(6)} = \frac{13}{28}(231\cos^6\theta - 315\cos^4\theta + 105\cos^2\theta - 5)(2e_\sigma - 3e_\pi) \tag{S18}$$

$$B_{\pm 4}^{(4)} = 3\sqrt{\frac{5}{14}}(\cos^2\theta - 1)^2\cos(2\phi)(3e_\sigma + e_\pi) \tag{S19}$$

$$B_{\pm 4}^{(6)} = \frac{39}{4\sqrt{14}}(\cos^2\theta - 1)^2(11\cos^2\theta - 1)\cos(2\phi)(2e_\sigma - 3e_\pi) \tag{S20}$$

Note that the $\cos(2\phi)$ factor causes the $B_{\pm 4}^{(4)}$ and $B_{\pm 4}^{(6)}$ parameters to equal zero when $\phi = 45°$ (as in a perfect D_{4d} geometry).

S2.3 Construction of Symmetry-Adapted Wavefunctions

The Russell–Saunders (LS) basis is natural for use in the free lanthanide ion, and in this basis every $4f^2$ state can be uniquely labeled with its quantum numbers as $|SLJM\rangle$.† The introduction of a crystal field will further break the degeneracies of these states depending on how they transform in the appropriate double group. It is useful to transform the Hamiltonian matrix into wavefunctions that are adapted to conform to irreps of the (double) group because the Hamiltonian matrix must be block diagonalizable into submatrices corresponding to these irreps.[14]

Irreps were assigned to each $|SLJM\rangle$ state by calculating the wavefunction's character with respect to each symmetry operation. As an example, checking the symmetry properties of the $|^1D_2, M\rangle$ levels upon a $C_4^+(z)$ operation and a $C_2'(x)$ operation involves inspecting the $^1D_2$ submatrix of the $\exp\left(-i\pi/2\hat{J}_z\right)$ and $\exp\left(-i\pi\hat{J}_x\right)$ operators, respectively:

$$\underbrace{\begin{array}{c|ccccc} & |+2\rangle & |+1\rangle & |0\rangle & |-1\rangle & |-2\rangle \\ \hline \langle +2| & -1 & 0 & 0 & 0 & 0 \\ \langle +1| & 0 & -i & 0 & 0 & 0 \\ \langle 0| & 0 & 0 & 1 & 0 & 0 \\ \langle -1| & 0 & 0 & 0 & i & 0 \\ \langle -2| & 0 & 0 & 0 & 0 & -1 \end{array}}_{C_4^+} \quad \underbrace{\begin{array}{c|ccccc} & |+2\rangle & |+1\rangle & |0\rangle & |-1\rangle & |-2\rangle \\ \hline \langle +2| & 0 & 0 & 0 & 0 & 1 \\ \langle +1| & 0 & 0 & 0 & 1 & 0 \\ \langle 0| & 0 & 0 & 1 & 0 & 0 \\ \langle -1| & 0 & 1 & 0 & 0 & 0 \\ \langle -2| & 1 & 0 & 0 & 0 & 0 \end{array}}_{C_2'(x)} \tag{S21}$$

The off-diagonal elements of the $^1D_2$ $C_2'(x)$ submatrix clearly show that $M = \pm 2$ transform together, as do $M = \pm 1$; thus, we rearrange to

$$\underbrace{\begin{array}{c|ccccc} & |0\rangle & |+1\rangle & |-1\rangle & |+2\rangle & |-2\rangle \\ \hline \langle 0| & 1 & 0 & 0 & 0 & 0 \\ \langle +1| & 0 & -i & 0 & 0 & 0 \\ \langle -1| & 0 & 0 & i & 0 & 0 \\ \langle +2| & 0 & 0 & 0 & -1 & 0 \\ \langle -2| & 0 & 0 & 0 & 0 & -1 \end{array}}_{C_4^+} \quad \underbrace{\begin{array}{c|ccccc} & |0\rangle & |+1\rangle & |-1\rangle & |+2\rangle & |-2\rangle \\ \hline \langle 0| & 1 & 0 & 0 & 0 & 0 \\ \langle +1| & 0 & 0 & 1 & 0 & 0 \\ \langle -1| & 0 & 1 & 0 & 0 & 0 \\ \langle +2| & 0 & 0 & 0 & 0 & 1 \\ \langle -2| & 0 & 0 & 0 & 1 & 0 \end{array}}_{C_2'(x)} \tag{S22}$$

†The fuller $SU(2)^S \otimes (SO(7) \supset G_2 \supset SO(3))^L$ classification is not needed for a f^2 ion because there are no repeat $SLJM$ states with only two electrons. For other ions, nomenclature based on non-symmetry Lie groups is used to distinguish between states with shared $SLJM$ quantum numbers. The states are named $|\tau WUSLJM\rangle$, where W is an irrep of $SO(7)$, U is an irrep of G_2, and τ is a disambiguation index only used in certain states of f^{5-9} ions. See Nielson and Koster for a tabulation of these irrep values.[13]

Table S2: Matrix representatives from the D_{4d} and D_4 group representations in Altmann[11]

Op	D_{4d} Irreps							D_4 Irreps				
	A_1	A_2	B_1	B_2	E_1	E_2	E_3	A_1	A_2	B_1	B_2	E
E	(1)	(1)	(1)	(1)	$\begin{pmatrix}1&0\\0&1\end{pmatrix}$	$\begin{pmatrix}1&0\\0&1\end{pmatrix}$	$\begin{pmatrix}1&0\\0&1\end{pmatrix}$	(1)	(1)	(1)	(1)	$\begin{pmatrix}1&0\\0&1\end{pmatrix}$
C_4^+	(1)	(1)	(1)	(1)	$\begin{pmatrix}-i&0\\0&i\end{pmatrix}$	$\begin{pmatrix}-1&0\\0&-1\end{pmatrix}$	$\begin{pmatrix}-i&0\\0&i\end{pmatrix}$	(1)	(1)	(−1)	(−1)	$\begin{pmatrix}-i&0\\0&i\end{pmatrix}$
C_4^-	(1)	(1)	(1)	(1)	$\begin{pmatrix}i&0\\0&-i\end{pmatrix}$	$\begin{pmatrix}-1&0\\0&-1\end{pmatrix}$	$\begin{pmatrix}i&0\\0&-i\end{pmatrix}$	(1)	(1)	(−1)	(−1)	$\begin{pmatrix}i&0\\0&-i\end{pmatrix}$
C_2	(1)	(1)	(1)	(1)	$\begin{pmatrix}-1&0\\0&-1\end{pmatrix}$	$\begin{pmatrix}1&0\\0&1\end{pmatrix}$	$\begin{pmatrix}-1&0\\0&-1\end{pmatrix}$	(1)	(1)	(1)	(1)	$\begin{pmatrix}-1&0\\0&-1\end{pmatrix}$
C'_{21}	(1)	(−1)	(1)	(−1)	$\begin{pmatrix}0&-1\\-1&0\end{pmatrix}$	$\begin{pmatrix}0&1\\1&0\end{pmatrix}$	$\begin{pmatrix}0&-1\\-1&0\end{pmatrix}$	(1)	(−1)	(1)	(−1)	$\begin{pmatrix}0&-1\\-1&0\end{pmatrix}$
C'_{22}	(1)	(−1)	(1)	(−1)	$\begin{pmatrix}0&1\\1&0\end{pmatrix}$	$\begin{pmatrix}0&1\\1&0\end{pmatrix}$	$\begin{pmatrix}0&1\\1&0\end{pmatrix}$	(1)	(−1)	(1)	(−1)	$\begin{pmatrix}0&1\\1&0\end{pmatrix}$
C'_{23}	(1)	(−1)	(1)	(−1)	$\begin{pmatrix}0&i\\-i&0\end{pmatrix}$	$\begin{pmatrix}0&-1\\-1&0\end{pmatrix}$	$\begin{pmatrix}0&i\\-i&0\end{pmatrix}$	(1)	(−1)	(−1)	(1)	$\begin{pmatrix}0&i\\-i&0\end{pmatrix}$
C'_{24}	(1)	(−1)	(1)	(−1)	$\begin{pmatrix}0&-i\\i&0\end{pmatrix}$	$\begin{pmatrix}0&-1\\-1&0\end{pmatrix}$	$\begin{pmatrix}0&-i\\i&0\end{pmatrix}$	(1)	(−1)	(−1)	(1)	$\begin{pmatrix}0&-i\\i&0\end{pmatrix}$
S_8^{3-}	(1)	(1)	(−1)	(−1)	$\begin{pmatrix}-\epsilon^*&0\\0&-\epsilon\end{pmatrix}$	$\begin{pmatrix}-i&0\\0&i\end{pmatrix}$	$\begin{pmatrix}\epsilon^*&0\\0&\epsilon\end{pmatrix}$					
S_8^{3+}	(1)	(1)	(−1)	(−1)	$\begin{pmatrix}-\epsilon&0\\0&-\epsilon^*\end{pmatrix}$	$\begin{pmatrix}i&0\\0&-i\end{pmatrix}$	$\begin{pmatrix}\epsilon&0\\0&\epsilon^*\end{pmatrix}$					
S_8^-	(1)	(1)	(−1)	(−1)	$\begin{pmatrix}\epsilon&0\\0&\epsilon^*\end{pmatrix}$	$\begin{pmatrix}i&0\\0&-i\end{pmatrix}$	$\begin{pmatrix}-\epsilon&0\\0&-\epsilon^*\end{pmatrix}$					
S_8^+	(1)	(1)	(−1)	(−1)	$\begin{pmatrix}\epsilon^*&0\\0&\epsilon\end{pmatrix}$	$\begin{pmatrix}-i&0\\0&i\end{pmatrix}$	$\begin{pmatrix}-\epsilon^*&0\\0&-\epsilon\end{pmatrix}$					
σ_{d1}	(1)	(−1)	(−1)	(1)	$\begin{pmatrix}0&\epsilon^*\\\epsilon&0\end{pmatrix}$	$\begin{pmatrix}0&-i\\i&0\end{pmatrix}$	$\begin{pmatrix}0&-\epsilon^*\\-\epsilon&0\end{pmatrix}$					
σ_{d2}	(1)	(−1)	(−1)	(1)	$\begin{pmatrix}0&-\epsilon^*\\-\epsilon&0\end{pmatrix}$	$\begin{pmatrix}0&-i\\i&0\end{pmatrix}$	$\begin{pmatrix}0&\epsilon^*\\\epsilon&0\end{pmatrix}$					
σ_{d3}	(1)	(−1)	(−1)	(1)	$\begin{pmatrix}0&-\epsilon\\-\epsilon^*&0\end{pmatrix}$	$\begin{pmatrix}0&i\\-i&0\end{pmatrix}$	$\begin{pmatrix}0&\epsilon\\\epsilon^*&0\end{pmatrix}$					
σ_{d4}	(1)	(−1)	(−1)	(1)	$\begin{pmatrix}0&\epsilon\\\epsilon^*&0\end{pmatrix}$	$\begin{pmatrix}0&i\\-i&0\end{pmatrix}$	$\begin{pmatrix}0&-\epsilon\\-\epsilon^*&0\end{pmatrix}$					

$\epsilon = \exp(2\pi i/8) = (1+i)/\sqrt{2}$. See Altmann for a description of the orientations of the C'_2 axes and σ_d planes.

where we can see three blocks (1×1, 2×2, 2×2). Within each block, the trace reveals the character of the representation, so repetition for every symmetry operation of D_{4d} allows assignments of irreps to each of these blocks (A_1 for 0, E_3 for ±1, E_2 for ±2). The following Eq. (S23) defines a similarity transform that allows all blocks to match the standardized matrices listed in Table S2 and defines the symmetry-adapted wavefunctions of the $^1D_2$ levels:

$$\begin{aligned} &|^1D_2, A_1\rangle = |^1D_2, 0\rangle \\ &|^1D_2, E_3, 1\rangle = |^1D_2, 1\rangle \qquad |^1D_2, E_3, 2\rangle = -|^1D_2, -1\rangle \\ &|^1D_2, E_2, 1\rangle = |^1D_2, 2\rangle \qquad |^1D_2, E_2, 2\rangle = |^1D_2, -2\rangle \end{aligned} \tag{S23}$$

where the $|^{2S+1}L_J, \Gamma, \gamma\rangle$ nomenclature indicates the LS state ($^{2S+1}L_J$), the irrep of the level (Γ), and (if Γ happens to be degenerate) γ indicates which component of Γ (in the Altmann convention) is being discussed. In this basis, the previous two submatrices are transformed into

$$\underbrace{\begin{array}{c|ccccc} & |A_1\rangle & |E_3,1\rangle & |E_3,2\rangle & |E_2,1\rangle & |E_2,2\rangle \\ \hline \langle A_1| & 1 & 0 & 0 & 0 & 0 \\ \langle E_3,1| & 0 & -i & 0 & 0 & 0 \\ \langle E_3,2| & 0 & 0 & i & 0 & 0 \\ \langle E_2,1| & 0 & 0 & 0 & -1 & 0 \\ \langle E_2,2| & 0 & 0 & 0 & 0 & -1 \end{array}}_{C_4^+} \qquad \underbrace{\begin{array}{c|ccccc} & |A_1\rangle & |E_3,1\rangle & |E_3,2\rangle & |E_2,1\rangle & |E_2,2\rangle \\ \hline \langle A_1| & 1 & 0 & 0 & 0 & 0 \\ \langle E_3,1| & 0 & 0 & -1 & 0 & 0 \\ \langle E_3,2| & 0 & -1 & 0 & 0 & 0 \\ \langle E_2,1| & 0 & 0 & 0 & 0 & 1 \\ \langle E_2,2| & 0 & 0 & 0 & 1 & 0 \end{array}}_{C'_2(x)} \tag{S24}$$

and this matches the group representations listed in Table S2.

Application of this technique to all $4f^2$ states showed a simple pattern relating the M_J levels within each state to symmetry-adapted wavefunctions. The singly-degenerate irreps are easy to correlate with M levels: for the $M = 0$ levels we get a relation that varies by the value of J,

$$|0\rangle = \begin{cases} |A_1\rangle & \text{if } J \text{ even} \\ |A_2\rangle & \text{if } J \text{ odd} \end{cases}, \tag{S25}$$

and for the $M = \pm 4$ levels we get two linear combinations,

$$|B_1\rangle = \frac{(-1)^J|{+4}\rangle + |{-4}\rangle}{\sqrt{2}}, \qquad |B_2\rangle = \frac{(-1)^J|{+4}\rangle - |{-4}\rangle}{\sqrt{2}}. \tag{S26}$$

For the doubly-degenerate irreps, we get

$$\begin{aligned} |{+1}\rangle &= (-1)^J|E_3, 1\rangle & |{-1}\rangle &= -|E_3, 2\rangle \\ |{+2}\rangle &= (-1)^J|E_2, 1\rangle & |{-2}\rangle &= |E_2, 2\rangle \\ |{+3}\rangle &= -|E_1, 2\rangle & |{-3}\rangle &= (-1)^J|E_1, 1\rangle \\ |{+5}\rangle &= (-1)^J|E_1, 1\rangle & |{-5}\rangle &= -|E_1, 2\rangle \\ |{+6}\rangle &= -|E_2, 2\rangle & |{-6}\rangle &= (-1)^J|E_2, 1\rangle \end{aligned} \tag{S27}$$

where there is some variation in relative phase depending on whether J is even or odd. In states with $J > 4$, repeated E_1 and E_2 levels appear. As a consequence, there will be intrastate mixing of the levels sharing irreps and the observed properties (such as g value and/or MCD/MLD signal) will change as a consequence of this mixing. The relations shown in Eqs. (S25)–(S27) inform the leftmost column of Table 1 in the main manuscript.

S3 Theoretical Treatment of MCD/MLD Signs and Saturation Curves

S3.1 Transition Dipole Moments and the Wigner–Eckart Theorem

Both MCD and MLD intensity formulas require use of the transition dipole moment operator. Because the system of interest has axial symmetry, we want to use the transformation properties of the symmetry-adapted wavefunctions to assist in the calculation of transition dipole matrix elements between states. We turn to the Wigner–Eckart theorem (WET) to do so. The WET is particularly useful for our purposes because it can easily indicate when a particular matrix element must be zero due to symmetry. For atomic systems (which use the $SO(3)/SU(2)$ Lie group), it usually takes the form in Eq. (S3) in lanthanide spectroscopy studies. Unfortunately, there are many other conventions, and the problem of alternate conventions becomes even more challenging when adapting the atomic WET into molecular point (double) groups.

In order to continue to use the Altmann tables,[11] we will adopt the WET convention defined in their introduction,[‡]

$$\langle \psi^A_\alpha | \hat{O}^C_\gamma | \phi^B_\beta \rangle = \langle \psi^A \| \hat{O}^C \| \phi^B \rangle (C\gamma, B\beta | A\alpha)^* \tag{S28}$$

To motivate this equation, let's assume that we want to calculate a matrix element $\langle \psi | \hat{O} | \phi \rangle$ for some states ψ and ϕ and some operator $\hat{O}$. If these states and operators have well-defined transformation properties that correspond to specific irreps (or irrep components) within the point/double group, then we can make use of this symmetry. We assume ψ transforms like irrep A component α, ϕ transforms like irrep B component β, and $\hat{O}$ transforms like irrep C component γ, and we indicate this by switching $\langle \psi | \hat{O} | \phi \rangle \to \langle \psi^A_\alpha | \hat{O}^C_\gamma | \phi^B_\beta \rangle$. Once we know these transformation properties, Eq. (S28) tells us that the value of this matrix element is proportional to a Clebsch–Gordan coefficient $(C\gamma, B\beta | A\alpha)$. The proportionality constant itself is called a 'reduced matrix element' and it is usually written with the double-vertical-bars $\langle \psi^A \| \hat{O}^C \| \phi^B \rangle$. Note that the reduced matrix element *does not depend on* α*,* β*, or* γ*!* This means that $\langle \psi^A_\alpha | \hat{O}^C_\gamma | \phi^B_\beta \rangle$ must equal zero if $(C\gamma, B\beta | A\alpha)$ does. The Clebsch–Gordan coefficients are always zero if $A \notin B \otimes C$. For cases when $A \in B \otimes C$, Altmann tabulates these Clebsch–Gordan coefficients only when there are degenerate states involved. Among singly degenerate irreps, the Clebsch–Gordan coefficient is taken to be 1 if $A = B \otimes C$ and 0 otherwise.

The transition dipole moment operator transforms like $\Gamma(\hat{\vec{m}}) = \Gamma(x, y, z)$, and in D_{4d} this becomes $\Gamma(z) = B_2$ and $\Gamma(x, y) = E_1$. In order to use Eq. (S28) we also need to use symmetry-adapted operators, and the symmetrized bases in Altmann indicate that the following operators will work:

$$\hat{m}^{B_2} = \hat{m}_z, \qquad \hat{m}^{E_1}_1 = -\frac{1}{\sqrt{2}} (\hat{m}_x + i\hat{m}_y) = \hat{m}_{\mathrm{LCP}}, \qquad \hat{m}^{E_1}_2 = +\frac{1}{\sqrt{2}} (\hat{m}_x - i\hat{m}_y) = \hat{m}_{\mathrm{RCP}} \tag{S29}$$

or in reverse,

$$\hat{m}_z = \hat{m}^{B_2}, \qquad \hat{m}_x = -\frac{1}{\sqrt{2}} \left(\hat{m}^{E_1}_1 - \hat{m}^{E_1}_2 \right), \qquad \hat{m}_y = \frac{i}{\sqrt{2}} \left(\hat{m}^{E_1}_1 + \hat{m}^{E_1}_2 \right) \tag{S30}$$

As an example use case, we can look at the submatrices for m^{B_2}, $m^{E_1}_1$, and $m^{E_1}_2$ connecting the $^3H_4$ state

‡Here we have switched away from the notation used in Altmann, which states the WET as $\langle IP | ijmn \rangle = \sum_U \langle I \| IU \rangle^{ij} \langle mn | IUP \rangle^\dagger$. We have also removed the sum over repeated irreps because neither D_{4d} nor D_4 contain repeated representations in their direct products.

with the $^3P_1$ state:

$$
m^{B_2}: \quad \begin{array}{r} \\ \langle ^3H_4, A_1| \\ \langle ^3H_4, E_3, 1| \\ \langle ^3H_4, E_3, 2| \\ \langle ^3H_4, E_2, 1| \\ \langle ^3H_4, E_2, 2| \\ \langle ^3H_4, E_1, 1| \\ \langle ^3H_4, E_1, 2| \\ \langle ^3H_4, B_1| \\ \langle ^3H_4, B_2| \end{array}
\begin{array}{c} |^3P_1, A_2\rangle \quad\quad |^3P_1, E_3, 1\rangle \quad\quad |^3P_1, E_3, 2\rangle \\
\begin{pmatrix}
0 & 0 & 0 \\
0 & 0 & 0 \\
0 & 0 & 0 \\
0 & 0 & 0 \\
0 & 0 & 0 \\
0 & \langle ^3H_4, E_1 \| m^{B_2} \| ^3P_1, E_3\rangle & 0 \\
0 & 0 & -\langle ^3H_4, E_1 \| m^{B_2} \| ^3P_1, E_3\rangle \\
\langle ^3H_4, B_2 \| m^{B_2} \| ^3P_1, A_2\rangle & 0 & 0 \\
0 & 0 & 0
\end{pmatrix} \end{array}
$$

$$
m_1^{E_1}: \quad \begin{array}{r} \\ \langle ^3H_4, A_1| \\ \langle ^3H_4, E_3, 1| \\ \langle ^3H_4, E_3, 2| \\ \langle ^3H_4, E_2, 1| \\ \langle ^3H_4, E_2, 2| \\ \langle ^3H_4, E_1, 1| \\ \langle ^3H_4, E_1, 2| \\ \langle ^3H_4, B_1| \\ \langle ^3H_4, B_2| \end{array}
\begin{array}{c} |^3P_1, A_2\rangle \quad\quad |^3P_1, E_3, 1\rangle \quad\quad |^3P_1, E_3, 2\rangle \\
\begin{pmatrix}
0 & 0 & 0 \\
0 & 0 & 0 \\
0 & 0 & 0 \\
0 & 0 & 0 \\
0 & \langle ^3H_4, E_2 \| m^{E_1} \| ^3P_1, E_3\rangle & 0 \\
\langle ^3H_4, E_1 \| m^{E_1} \| ^3P_1, A_2\rangle & 0 & 0 \\
0 & 0 & 0 \\
0 & 0 & \frac{1}{\sqrt{2}}\langle ^3H_4, B_1 \| m^{E_1} \| ^3P_1, E_3\rangle \\
0 & 0 & \frac{1}{\sqrt{2}}\langle ^3H_4, B_2 \| m^{E_1} \| ^3P_1, E_3\rangle
\end{pmatrix} \end{array} \tag{S31}
$$

$$
m_2^{E_1}: \quad \begin{array}{r} \\ \langle ^3H_4, A_1| \\ \langle ^3H_4, E_3, 1| \\ \langle ^3H_4, E_3, 2| \\ \langle ^3H_4, E_2, 1| \\ \langle ^3H_4, E_2, 2| \\ \langle ^3H_4, E_1, 1| \\ \langle ^3H_4, E_1, 2| \\ \langle ^3H_4, B_1| \\ \langle ^3H_4, B_2| \end{array}
\begin{array}{c} |^3P_1, A_2\rangle \quad\quad |^3P_1, E_3, 1\rangle \quad\quad |^3P_1, E_3, 2\rangle \\
\begin{pmatrix}
0 & 0 & 0 \\
0 & 0 & 0 \\
0 & 0 & 0 \\
0 & 0 & \langle ^3H_4, E_2 \| m^{E_1} \| ^3P_1, E_3\rangle \\
0 & 0 & 0 \\
0 & 0 & 0 \\
-\langle ^3H_4, E_1 \| m^{E_1} \| ^3P_1, A_2\rangle & 0 & 0 \\
0 & \frac{1}{\sqrt{2}}\langle ^3H_4, B_1 \| m^{E_1} \| ^3P_1, E_3\rangle & 0 \\
0 & -\frac{1}{\sqrt{2}}\langle ^3H_4, B_2 \| m^{E_1} \| ^3P_1, E_3\rangle & 0
\end{pmatrix} \end{array}
$$

Thus, despite the fact that we have three 9×3 submatrices ($3 \times 27 = 81$ entries total), there are only six unknown scalars (reduced matrix elements) needed to define all of the matrix elements: $\langle ^3H_4, E_1 \| m^{B_2} \| ^3P_1, E_3\rangle$, $\langle ^3H_4, B_2 \| m^{B_2} \| ^3P_1, A_2\rangle$, $\langle ^3H_4, E_2 \| m^{E_1} \| ^3P_1, E_3\rangle$, $\langle ^3H_4, E_1 \| m^{E_1} \| ^3P_1, A_2\rangle$, $\langle ^3H_4, B_1 \| m^{E_1} \| ^3P_1, E_3\rangle$, and $\langle ^3H_4, B_2 \| m^{E_1} \| ^3P_1, E_3\rangle$. Depending on the nature of the system and the experiment, these can be calculated (if needed) in a number of ways: estimation from experiment, computational methods, Judd–Ofelt theory, etc.

S3.2 MCD/MLD Intensity Expressions and Orientational Averaging

Intensity expressions for these spectroscopies can be expressed as

$$
\frac{\Delta\epsilon_{\text{MCD}}}{E} = \gamma \sum_{\substack{a\in A \\ j\in J}} (N_a - N_j) \left(|\langle a|\hat{m}_-|j\rangle|^2 - |\langle a|\hat{m}_+|j\rangle|^2 \right) f_{aj}(E) \tag{S32}
$$

$$
\frac{\Delta\epsilon_{\text{MLD}}}{E} = \gamma \sum_{\substack{a\in A \\ j\in J}} (N_a - N_j) \left(|\langle a|\hat{m}_\parallel|j\rangle|^2 - |\langle a|\hat{m}_\perp|j\rangle|^2 \right) f_{aj}(E) \tag{S33}
$$

These expressions are both written in the laboratory frame, where the direction of the applied magnetic field is defined as the $\vec{z}$ direction. MCD experiments have the direction of propagation of light oriented longitudinally

along this same $\vec{z}$ direction, whereas MLD experiments orient the light in a transverse orientation. For dissolved molecules in a frozen glass (or even in liquid solution), the orientation of molecules with respect to the field and light will be isotropically distributed; thus, the net MCD or MLD intensity should be calculated by integrating over all molecular orientations.

It will prove easier to perform the integration in the molecular frame. In preparation, we start by noting

$$\begin{aligned}
|\langle a|\hat{m}_-|j\rangle|^2 - |\langle a|\hat{m}_+|j\rangle|^2 &= \left|\frac{1}{\sqrt{2}}\langle a|(\hat{m}_x - i\hat{m}_y)|j\rangle\right|^2 - \left|-\frac{1}{\sqrt{2}}\langle a|(\hat{m}_x + i\hat{m}_y)|j\rangle\right|^2 \\
&= \frac{1}{2}\langle a|(\hat{m}_x - i\hat{m}_y)|j\rangle\langle j|(\hat{m}_x + i\hat{m}_y)|a\rangle - \frac{1}{2}\langle a|(\hat{m}_x + i\hat{m}_y)|j\rangle\langle j|(\hat{m}_x - i\hat{m}_y)|a\rangle \\
&= i\langle a|\hat{m}_x|j\rangle\langle j|\hat{m}_y|a\rangle - i\langle a|\hat{m}_y|j\rangle\langle j|\hat{m}_x|a\rangle \\
&= i\vec{z}_{\text{lab}} \cdot \langle a|\hat{\vec{m}}|j\rangle \times \langle j|\hat{\vec{m}}|a\rangle
\end{aligned} \tag{S34}$$

Thus, the MCD expression can be rewritten[§]

$$\frac{\Delta\epsilon_{\text{MCD}}}{E} = i\gamma \sum_{\substack{a\in A\\ j\in J}} (N_a - N_j)\, \vec{z}_{\text{lab}} \cdot \Big(\langle a|\hat{\vec{m}}|j\rangle \times \langle j|\hat{\vec{m}}|a\rangle\Big)\, f_{aj}(E). \tag{S35}$$

This equation works equally well in the molecular frame because we can simply allow $\vec{z}_{\text{lab}}$ to deviate from the molecular z axis. The observed MCD intensity will then result from the integration over all orientations of $\hat{z}_{\text{lab}} = (\cos\phi\sin\theta, \sin\phi\sin\theta, \cos\theta)$ (the direction of both the field and the light),

$$\left\langle \frac{\Delta\epsilon_{\text{MCD}}}{E} \right\rangle = \iint i\gamma \sum_{\substack{a\in A\\ j\in J}} (N_a - N_j)\, \vec{z}_{\text{lab}} \cdot \Big(\langle a|\hat{\vec{m}}|j\rangle \times \langle j|\hat{\vec{m}}|a\rangle\Big)\, f_{aj}(E)\, \sin\theta d\theta d\phi. \tag{S36}$$

Here, we have used angular brackets to indicate an orientationally averaged quantity. In this expression, note that $|a\rangle$, $|j\rangle$, N_a, N_j, $\vec{z}_{\text{lab}}$, and $f_{aj}(E)$ all vary with θ and ϕ, and this integration cannot generally be performed analytically.

For MLD intensity, we can approach orientational averaging in a similar way but we also must integrate over the azimuthal angle of the light with respect to the field. If we take the direction of the magnetic field as the laboratory $\vec{z}_{\text{lab}}$ axis, our orientational averaging will require that we integrate over all orientations of light in the $\vec{x}_{\text{lab}}/\vec{y}_{\text{lab}}$ plane. This means we need to integrate over θ and ϕ to define the magnetic field direction, and also a third angle ψ to define the direction of light perpendicularly. We start by integrating over ψ by making the substitution

$$\begin{aligned}
\left|\langle a|\hat{m}_\parallel|j\rangle\right|^2 - |\langle a|\hat{m}_\perp|j\rangle|^2 &\to \frac{1}{2\pi}\int_0^{2\pi} \Big(|\langle a|\hat{m}_z|j\rangle|^2 - |\langle a|\left(\cos\psi\,\hat{m}_x + \sin\psi\,\hat{m}_y\right)|j\rangle|^2\Big)\, d\psi \\
&= \langle a|\hat{m}_z|j\rangle\langle j|\hat{m}_z|a\rangle - \frac{1}{2}\langle a|\hat{m}_x|j\rangle\langle j|\hat{m}_x|a\rangle - \frac{1}{2}\langle a|\hat{m}_y|j\rangle\langle j|\hat{m}_y|a\rangle \\
&= \frac{3}{2}(\langle a|\hat{\vec{m}}|j\rangle \cdot \vec{z}_{\text{lab}})(\vec{z}_{\text{lab}} \cdot \langle j|\hat{\vec{m}}|a\rangle))|^2 - \frac{1}{2}\langle a|\hat{\vec{m}}|j\rangle \cdot \langle j|\hat{\vec{m}}|a\rangle
\end{aligned} \tag{S37}$$

and we can see that this expression relies only on the orientation of the laboratory $\vec{z}_{\text{lab}}$ axis. Thus, the MLD expression after orientational averaging *only in* ψ can now be written

$$\left\langle \frac{\Delta\epsilon_{\text{MLD}}}{E} \right\rangle_{\psi\text{ only}} = \gamma \sum_{\substack{a\in A\\ j\in J}} (N_a - N_j)\, \langle a|\hat{\vec{m}}|j\rangle \cdot \left(\frac{3}{2}\vec{z}_{\text{lab}}\vec{z}_{\text{lab}}^{\mathsf{T}} - \frac{1}{2}\right) \cdot \langle j|\hat{\vec{m}}|a\rangle f_{aj}(E) \tag{S38}$$

Here, $\vec{z}_{\text{lab}}\vec{z}_{\text{lab}}^{\mathsf{T}}$ is an outer product (a dyadic, a 3×3 matrix). This same expression can be used in the molecular frame if we allow $\vec{z}_{\text{lab}}$ to deviate from the molecular axis, giving MLD intensity after orientational

[§]NB: Because the cross product is of a vector with its complex conjugate, the cross product will be purely imaginary; thus, the i at the front of the Eq. (S35) causes the overall value to be real.

averaging over all angles:

$$\left\langle \frac{\Delta\epsilon_{\text{MLD}}}{E} \right\rangle = \iint \gamma \sum_{\substack{a\in A\\ j\in J}} (N_a - N_j) \langle a|\hat{\vec{m}}|j\rangle \cdot \left(\frac{3}{2}\vec{z}_{\text{lab}}\vec{z}_{\text{lab}}^{\mathsf{T}} - \frac{1}{2}\right) \cdot \langle j|\hat{\vec{m}}|a\rangle f_{aj}(E)\, \sin\theta d\theta d\phi. \tag{S39}$$

Lastly, absorption can be subjected to this same treatment and we obtain the expression

$$\left\langle \frac{\epsilon_{\text{Abs}}}{E} \right\rangle = \iint \frac{\gamma}{2} \sum_{\substack{a\in A\\ j\in J}} (N_a - N_j) \langle a|\hat{\vec{m}}|j\rangle \cdot \left(1 - \vec{z}_{\text{lab}}\vec{z}_{\text{lab}}^{\mathsf{T}}\right) \cdot \langle j|\hat{\vec{m}}|a\rangle f_{aj}(E)\, \sin\theta d\theta d\phi. \tag{S40}$$

In Eqs. (S35), (S38), and (S40), the excited state J and all of its sublevels j are assumed to be too high in energy to be meaningfully Boltzmann populated; thus, we will always use $N_j \approx 0$.

We want to predict the signs of the MCD and MLD signals when a non-saturating magnetic field is applied, and a Taylor expansion about $B = 0$ is a useful way to gain this information. Using the BO–FC–RS approximations (see Section S6.1) and Taylor series expansion (Section S6.2), we are able to analytically evaluate Eqs. (S40) for absorption, (S35) for MCD, and (S38) for MLD if we make some simplifying assumptions. In truth, all levels sharing an irrep will mix; however, we will assume that there is no interstate mixing, and that there is no intrastate mixing between $\pm M$ levels sharing an irrep. For example, the $^3H_6$ state has an E_1 level for the $M = \mp 3$ levels and another E_1 level for the $M = \pm 5$ levels; we will assume there is no mixing between these two E_1 levels. This approximation leaves M as a 'good' quantum number. For states with strong intrastate mixing, our predictions for MCD/MLD signs and intensities may not hold up. Deviations from our predictions may also be found in the presence of interstate mixing (especially when two states get close in energy) and when the structural deviations from a perfect D_{4d} geometry cause D_{4d} irreps to become poor descriptors of symmetry.

The signs and relative intensities of absorption, MCD, and MLD Faraday terms after orientational averaging are shown in Table S3. Note that all $\bar{\mathcal{G}}_0$ terms were predicted to be 0 in agreement with Bominaar et al.[15] This table lists values as a function of the effective g values of the initial and final levels involved in the absorption event. The effective g values for all levels of a $4f^2$ ion in D_{4d} geometry are listed in Table S4. Plugging these effective g values into the equations shown in Table S3 produced the signs of Faraday parameters shown in the main manuscript.

Table S3: Absorption, MCD, and MLD Intensities of Transitions

Symmetries	Absorption	MCD		MLD		
initial $\to$ *final*	$\bar{\mathcal{D}}_0$	$\bar{\mathcal{A}}_1$	$\bar{\mathcal{C}}_0$	$\bar{\mathcal{A}}_2$	$\bar{\mathcal{C}}_1$	$\bar{\mathcal{G}}_0$
$A_1 \to A_1$	0	0	0	0	0	0
$\to A_2$	0	0	0	0	0	0
$\to B_1$	0	0	0	0	0	0
$\to B_2$	1/3	0	0	0	0	0
$\to E_1$	1/3	$g^{f}_{\mathrm{eff}}/3$	0	$-{g^{f}_{\mathrm{eff}}}^{2}/15$	0	0
$\to E_2$	0	0	0	0	0	0
$\to E_3$	0	0	0	0	0	0
$A_2 \to A_1$	0	0	0	0	0	0
$\to A_2$	0	0	0	0	0	0
$\to B_1$	1/3	0	0	0	0	0
$\to B_2$	0	0	0	0	0	0
$\to E_1$	1/3	$g^{f}_{\mathrm{eff}}/3$	0	$-{g^{f}_{\mathrm{eff}}}^{2}/15$	0	0
$\to E_2$	0	0	0	0	0	0
$\to E_3$	0	0	0	0	0	0
$B_1 \to A_1$	0	0	0	0	0	0
$\to A_2$	1/3	0	0	0	0	0
$\to B_1$	0	0	0	0	0	0
$\to B_2$	0	0	0	0	0	0
$\to E_1$	0	0	0	0	0	0
$\to E_2$	0	0	0	0	0	0
$\to E_3$	1/3	$g^{f}_{\mathrm{eff}}/3$	0	$-{g^{f}_{\mathrm{eff}}}^{2}/15$	0	0
$B_2 \to A_1$	1/3	0	0	0	0	0
$\to A_2$	0	0	0	0	0	0
$\to B_1$	0	0	0	0	0	0
$\to B_2$	0	0	0	0	0	0
$\to E_1$	0	0	0	0	0	0
$\to E_2$	0	0	0	0	0	0
$\to E_3$	1/3	$g^{f}_{\mathrm{eff}}/3$	0	$-{g^{f}_{\mathrm{eff}}}^{2}/15$	0	0
$E_1 \to A_1$	1/3	$g^{i}_{\mathrm{eff}}/3$	$g^{i}_{\mathrm{eff}}/3$	$-{g^{i}_{\mathrm{eff}}}^{2}/15$	$-{g^{i}_{\mathrm{eff}}}^{2}/15$	0
$\to A_2$	1/3	$g^{i}_{\mathrm{eff}}/3$	$g^{i}_{\mathrm{eff}}/3$	$-{g^{i}_{\mathrm{eff}}}^{2}/15$	$-{g^{i}_{\mathrm{eff}}}^{2}/15$	0
$\to B_1$	0	0	0	0	0	0
$\to B_2$	0	0	0	0	0	0
$\to E_1$	0	0	0	0	0	0
$\to E_2$	1/3	$(g^{f}_{\mathrm{eff}} - g^{i}_{\mathrm{eff}})/3$	$-g^{i}_{\mathrm{eff}}/3$	$-(g^{f}_{\mathrm{eff}} - g^{i}_{\mathrm{eff}})^2/15$	$(g^{f}_{\mathrm{eff}} - g^{i}_{\mathrm{eff}})g^{i}_{\mathrm{eff}}/15$	0
$\to E_3$	1/3	0	0	0	$-2(g^{f}_{\mathrm{eff}} - g^{i}_{\mathrm{eff}})g^{i}_{\mathrm{eff}}/15$	0
$E_2 \to A_1$	0	0	0	0	0	0
$\to A_2$	0	0	0	0	0	0
$\to B_1$	0	0	0	0	0	0
$\to B_2$	0	0	0	0	0	0
$\to E_1$	1/3	$-(g^{f}_{\mathrm{eff}} - g^{i}_{\mathrm{eff}})/3$	$g^{i}_{\mathrm{eff}}/3$	$-(g^{f}_{\mathrm{eff}} - g^{i}_{\mathrm{eff}})^2/15$	$(g^{f}_{\mathrm{eff}} - g^{i}_{\mathrm{eff}})g^{i}_{\mathrm{eff}}/15$	0
$\to E_2$	1/3	0	0	0	$2(g^{f}_{\mathrm{eff}} + g^{i}_{\mathrm{eff}})g^{i}_{\mathrm{eff}}/15$	0
$\to E_3$	1/3	$-(g^{f}_{\mathrm{eff}} + g^{i}_{\mathrm{eff}})/3$	$-g^{i}_{\mathrm{eff}}/3$	$-(g^{f}_{\mathrm{eff}} + g^{i}_{\mathrm{eff}})^2/15$	$-(g^{f}_{\mathrm{eff}} + g^{i}_{\mathrm{eff}})g^{i}_{\mathrm{eff}}/15$	0
$E_3 \to A_1$	0	0	0	0	0	0
$\to A_2$	0	0	0	0	0	0
$\to B_1$	1/3	$g^{i}_{\mathrm{eff}}/3$	$g^{i}_{\mathrm{eff}}/3$	$-{g^{i}_{\mathrm{eff}}}^{2}/15$	$-{g^{i}_{\mathrm{eff}}}^{2}/15$	0
$\to B_2$	1/3	$g^{i}_{\mathrm{eff}}/3$	$g^{i}_{\mathrm{eff}}/3$	$-{g^{i}_{\mathrm{eff}}}^{2}/15$	$-{g^{i}_{\mathrm{eff}}}^{2}/15$	0
$\to E_1$	1/3	0	0	0	$-2(g^{f}_{\mathrm{eff}} - g^{i}_{\mathrm{eff}})g^{i}_{\mathrm{eff}}/15$	0
$\to E_2$	1/3	$-(g^{f}_{\mathrm{eff}} + g^{i}_{\mathrm{eff}})/3$	$-g^{i}_{\mathrm{eff}}/3$	$-(g^{f}_{\mathrm{eff}} + g^{i}_{\mathrm{eff}})^2/15$	$-(g^{f}_{\mathrm{eff}} + g^{i}_{\mathrm{eff}})g^{i}_{\mathrm{eff}}/15$	0
$\to E_3$	0	0	0	0	0	0

The g^{i}_{eff} and g^{f}_{eff} values are the effective g values for the initial and final levels. Each of the entries in this table should be multiplied by the magnitude squared of the reduced matrix element for the $i \to f$ transition, $|\langle i, \Gamma \| m^{\Gamma''} \| f, \Gamma' \rangle|^2$.

Table S4: Ideal g_{eff} Values When M Remains a Good Quantum Number

	g_{eff}		g_{eff}		g_{eff}
$^3H_4, 0, A_1$	0	$^3F_2, 0, A_1$	0	$^1D_2, 0, A_1$	0
$^3H_4, \pm1, E_3$	4/5	$^3F_2, \pm1, E_3$	2/3	$^1D_2, \pm1, E_3$	1
$^3H_4, \pm2, E_2$	8/5	$^3F_2, \pm2, E_2$	4/3	$^1D_2, \pm2, E_2$	2
$^3H_4, \pm3, E_1$	–12/5	$^3F_3, 0, A_2$	0	$^3P_0, 0, A_1$	0
$^3H_4, \pm4, B_1 + B_2$	16/5	$^3F_3, \pm1, E_3$	13/12	$^3P_1, 0, A_2$	0
$^3H_5, 0, A_2$	0	$^3F_3, \pm2, E_2$	13/6	$^3P_1, \pm1, E_3$	3/2
$^3H_5, \pm1, E_3$	31/30	$^3F_3, \pm3, E_1$	–13/4	$^3P_2, 0, A_1$	0
$^3H_5, \pm2, E_2$	31/15	$^3F_4, 0, A_1$	0	$^3P_2, \pm1, E_3$	3/2
$^3H_5, \pm3, E_1$	–31/10	$^3F_4, \pm1, E_3$	5/4	$^3P_2, \pm2, E_2$	3
$^3H_5, \pm4, B_1 + B_2$	62/15	$^3F_4, \pm2, E_2$	5/2	$^1I_6, 0, A_1$	0
$^3H_5, \pm5, E_1$	31/6	$^3F_4, \pm3, E_1$	–15/4	$^1I_6, \pm1, E_3$	1
$^3H_6, 0, A_1$	0	$^3F_4, \pm4, B_1 + B_2$	5	$^1I_6, \pm2, E_2$	2
$^3H_6, \pm1, E_3$	7/6	$^1G_4, 0, A_1$	0	$^1I_6, \pm3, E_1$	–3
$^3H_6, \pm2, E_2$	7/3	$^1G_4, \pm1, E_3$	1	$^1I_6, \pm4, B_1 + B_2$	4
$^3H_6, \pm3, E_1$	–7/2	$^1G_4, \pm2, E_2$	2	$^1I_6, \pm5, E_1$	5
$^3H_6, \pm4, B_1 + B_2$	14/3	$^1G_4, \pm3, E_1$	–3	$^1I_6, \pm6, E_2$	–6
$^3H_6, \pm5, E_1$	35/6	$^1G_4, \pm4, B_1 + B_2$	4	$^1S_0, 0, A_1$	0
$^3H_6, \pm6, E_2$	–7				

The $\pm$ signs follow from the correspondance of the $+M$ and $-M$ levels with components 1 and 2 within degenerate irreps; see Eqs. (S25)–(S27).

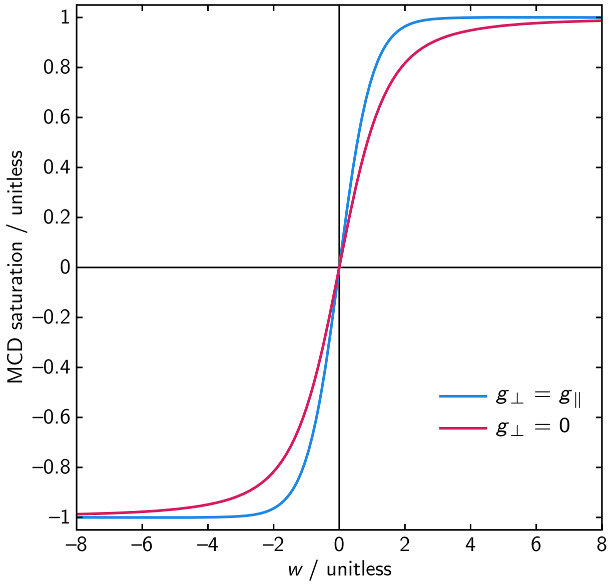

Figure S4: MCD saturation curves for $g_\perp = g_\parallel$ and $g_\perp = 0$ show different behavior. Here, $w = \mu_B B g_\parallel / k_B T$.

S3.3 MCD Saturation Curve of an Effective Doublet with $g_\perp = 0$

The MCD intensity expression in Eq. (S36) can be analytically evaluated under the limit that the ground state is doubly degenerate and the lowest-lying excited state is sufficiently high in energy that there is no appreciable mixing with or thermal population of this lowest excited state. This situation can be modeled as an effective Kramers doublet. Although $S = 1/2$ systems are typically modeled using an hyperbolic tangent function as $\Delta\epsilon_{\mathrm{MCD}}/E \propto \tanh\left(g\mu_B B/2k_B T\right)$, this saturation curve is not appropriate for the D_{4d} systems here. For a $4f^2$ D_{4d} system like **1**·Pr, it will always be the case that the effective $g_\perp = 0$ within the lowest-lying degenerate levels because $g_\perp$ can only deviate from 0 when $\Delta M = \pm 1$ within the effective Kramers levels. When $g_\perp = 0$, the Hamiltonian and density matrix take on particularly simple forms:

$$\hat{H} = \mu_B B g_\parallel S_z \cos\theta = \begin{pmatrix} -\frac{1}{2}\mu_B B g_\parallel \cos\theta & 0 \\ 0 & \frac{1}{2}\mu_B B g_\parallel \cos\theta \end{pmatrix} \tag{S41}$$

$$\hat{\rho} = \frac{1}{2}\begin{pmatrix} 1 + \tanh\left(\mu_B B g_\parallel \cos\theta / 2k_B T\right) & 0 \\ 0 & 1 - \tanh\left(\mu_B B g_\parallel \cos\theta / 2k_B T\right) \end{pmatrix} \tag{S42}$$

Plugging this density matrix into the MCD intensity equation and then integrating over θ and ϕ using a symbolic mathematical program like Wolfram (Mathematica) gives

$$\frac{\Delta\epsilon_{\mathrm{MCD}}}{E} \propto 1 - \frac{\pi^2}{12w^2} + \frac{2}{w}\ln\left(1 + e^{-2w}\right) - \frac{1}{w^2}\mathrm{Li}_2\left(-e^{-2w}\right), \quad \text{where } w := \frac{\mu_B B g_\parallel}{k_B T} \tag{S43}$$

for only the $\bar{\mathcal{C}}_0$ term intensity, where Li_2 is the dilogarithm. This expression saturates more slowly than the tanh expression (Fig. S4), which can be seen by taking a Taylor series expansion about $B = 0$:

$$\tanh(w) \approx w + O(w^2) \tag{S44}$$

$$1 - \frac{\pi^2}{12w^2} + \frac{2}{w}\ln\left(1 + e^{-2w}\right) - \frac{1}{w^2}\mathrm{Li}_2\left(-e^{-2w}\right) \approx \frac{2}{3}w + O(w^2) \tag{S45}$$

Thus, if one uses the hyperbolic tangent expression for saturation, an incorrect g value may be estimated.

Table S5: Lineshape fitting using Gaussian functions

State	Irrep	μ / cm^{-1}	σ / cm^{-1}	State	Irrep	μ / cm^{-1}	σ / cm^{-1}
	E_3	22328.0(1.1)	20.8(4)		A_1	7136.3(1.5)	18.2(1.6)
$^3P_2$	E_2	22285.0(1.1)	47.9(7)	$^3F_4$	E_3	7073.2(7)	20.1(4)
	A_1	22222.9(9)	20.8(4)		E_2	6894.6(2)	18.9(2)
$^1I_6$	*Roughly at 21450 cm^{-1}*				A_2	6581.3(2)	15.2(1)
$^3P_1$	E_3	21140.7(3)	23.8(4)	$^3F_3$	E_3	6520.2(3)	17.0(2)
$^3P_0$	A_1	20527.6(3)	22.0(3)		E_2	6480.7(3)	18.2(2)
	E_3	17084.9(2)	24.3(2)		A_1	5171.2(2)	13.2(1)
$^1D_2$	E_2	16794.8(3)	42.3(3)	$^3F_2$	E_2	5092.4(4)	15.8(2)
	A_1	16575.6(8)	17.3(6)		E_3	5090.5(2.0)	26.7(5)
$^1G_4$	*Roughly at 9700 cm^{-1}*			$^3H_6$	E_2	4894.1(7.0)	35(12)
					E_2	4587.0(1.8)	37.0(1.2)

Assignments of irreps come from analysis of the MCD and MLD signal patterns as discussed in the text. The $^1I_6$ and $^1G_4$ features were poorly resolved so only approximate energies are listed.

S4 MCD and MLD Spectra and Analysis

S4.1 Full Data Set and Assignments

The positions of transitions were fit with Gaussian lineshapes to determine the energies of the transitions observed. The formula used was

$$f(\mu, \sigma; x) = \frac{1}{\sqrt{2\pi}\sigma} \exp\left[-\frac{1}{2}\left(\frac{x-\mu}{\sigma}\right)^2\right], \qquad \text{(S46)}$$

where μ is a location parameter and σ is a scale parameter.

Lineshapes were simultaneously fit to all 58 MCD data sets and the MLD spectrum through the use of singular value decomposition (SVD). This was done by first performing an SVD on the 58 MCD data sets, retaining only the components with strong singular values (3 in Region 1, 2 in Region 2, 3 in Region 3, and 2 in Region 4). The MLD spectrum was then orthogonalized and normalized against these 2–3 orthonormalized MCD spectra with the strongest singular values. Together, these 3–4 orthonormalized 'basis' spectra were simultaneously fit to obtain the positions and widths of each feature. For the first 2–3 basis spectra corresponding to MCD data, mixtures of zeroth- and first-derivative Gaussians ($\bar{\mathcal{C}}_0$ and $\bar{\mathcal{A}}_1$ terms) were fit to each transition. For the basis spectrum arising from the MLD spectrum, mixtures of zeroth- and first-derivative Gaussians ($\bar{\mathcal{G}}_0$ and $\bar{\mathcal{C}}_1$ terms) were fit to each transition, except the $^3P_0$ transitions which had an additional second-derivative ($\bar{\mathcal{A}}_2$) component plainly visible. By forcing these 3–4 basis spectra to share position μ and shape σ parameters, we were able to estimate the energies of each transition with confidence, even in the presence of overlaps. Table S5 and Figure S5 summarize all of the fitted positions and lineshapes of the transitions. Further details on peaks within each region and on how irrep assignments were made are discussed below.

Before discussion of each individual region, it is also important to highlight that the MCD and MLD Faraday parameter sign predictions in Table 1 of the main manuscript assume non-saturating conditions. Those sign predictions were made using a Taylor series expansion of the MCD and MLD intensity equations to first and second order. In the presence of saturation effects in either MCD or MLD data, it is always possible to decrease the field to enter into a linear region (although practically signal-to-noise can become a challenge). When analyzing the signs of various transitions, it is important to ensure that there are no changes in sign in the MCD or MLD features as the field ramps from 0 T to 7 T. For our MCD spectra, we have done so by estimating the derivative of the MCD signal with respect to the strength of the applied field B at $B = 0$ using a saturation curve (Section S3.3), and this was used to judge the signs of $\bar{\mathcal{C}}_0$ and $\bar{\mathcal{A}}_1$ terms. For our MLD spectra, we did not find estimation of a second derivative at $B = 0$ to be sufficiently

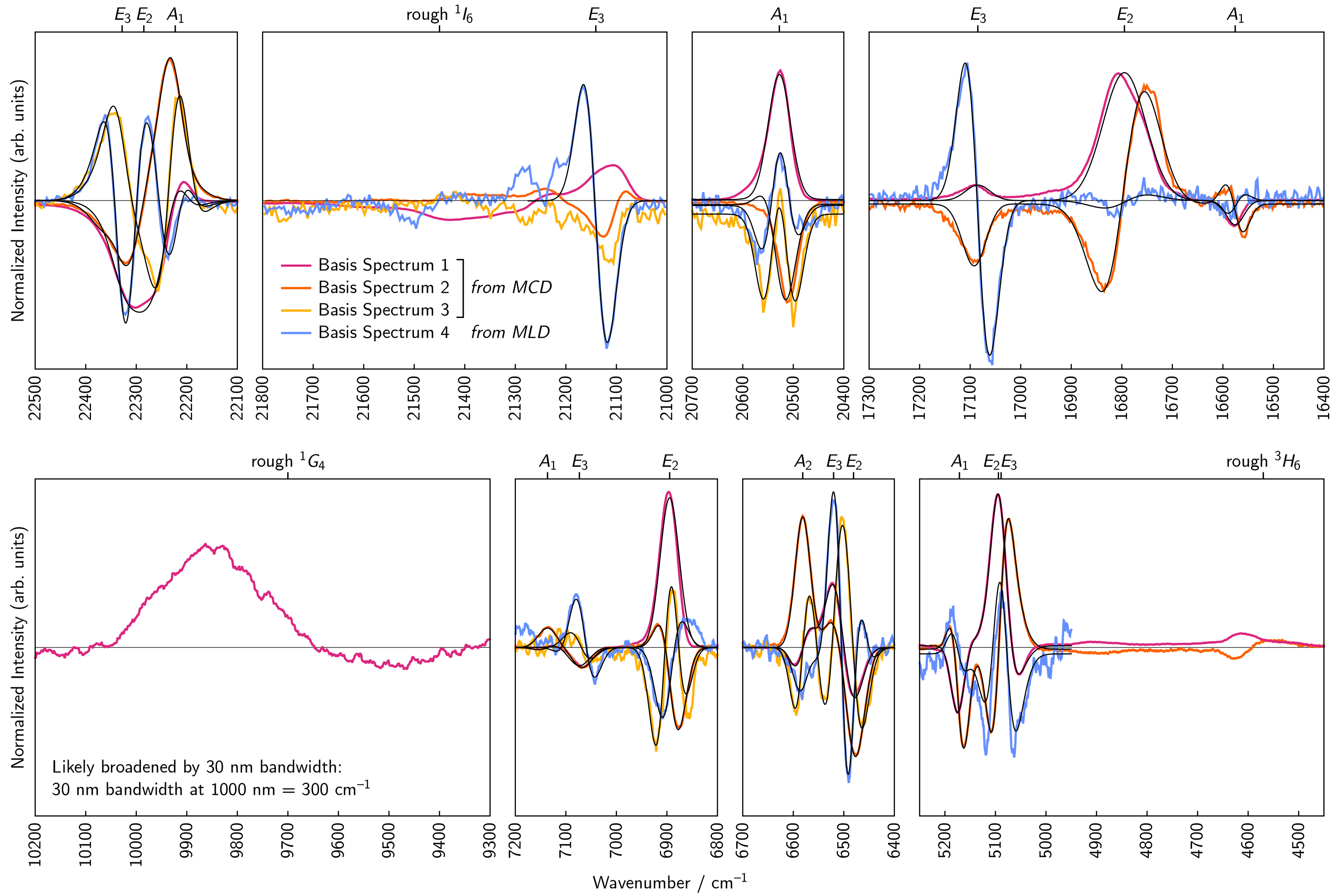

Figure S5: Lineshape fitting was performed to determine the positions of the transitions seen using a SVD process as described in the text. The 'basis spectra' from SVD are shown here along with the fits in black. Along the top of each subplot is an indication of the fitted energies (also seen in Table S5).

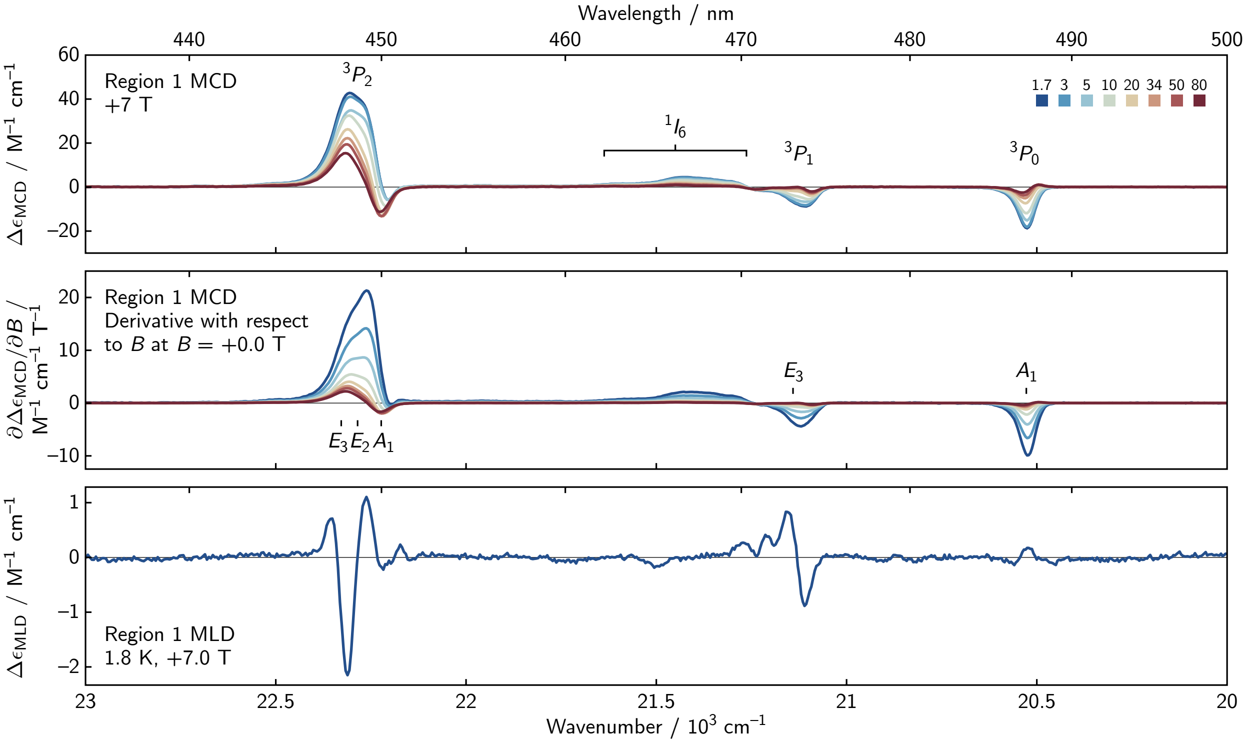

Figure S6: Cryogenic MCD and MLD spectra in a frozen 9:1 methanol-d_4/ethanol-d_6 in Region 1.

accurate; instead, we have collected the MLD spectra at a large number of field strengths (Fig. S3) to ensure that there are no observable changes in sign.

S4.1.1 Region 1

The spectra of Region 1 showed three groups of features (Fig. S6). Comparison with the transitions of atomic Pr^{III} suggests these arise from the $^3P_0$ state at the lowest energy, the $^3P_2$ state at the highest energy, and overlapping transitions of the $^3P_1$ and $^1I_6$ states in between.

The MCD associated with the $^3P_0$ transition at 20527.6(3) cm^{-1} had strongly negative $\bar{\mathcal{C}}_0$ intensity at low temperatures and revealed a negative $\bar{\mathcal{A}}_1$ intensity as the temperature rose to 80 K. The MLD spectrum showed intensity that could be modeled well with both negative $\bar{\mathcal{C}}_1$ and $\bar{\mathcal{A}}_2$ Faraday parameters. The strong intensity of the MCD feature and the uniformly negative MCD and MLD Faraday parameters (Table 1) are consistent with this feature arising from a $^3H_4(E_1) \to {}^3P_0(A_1)$ transition. This interpretation agrees with the AOM prediction of an E_1 ($M_J = \pm 3$) level lowest within the $^3H_4$ GS of **1**·Pr (Fig. 2).

The $^3P_2$ state provided a cluster of overlapping transitions in the 22200–22400 cm^{-1} window and the sharpness of MLD $\bar{\mathcal{C}}_1$ features were essential in their deconvolution. Within the D_{4d} double group, the $^3P_2$ state should split into $A_1 + E_3 + E_2$ levels. The low-energy side of the cluster showed both negative $\bar{\mathcal{C}}_0$ MCD and negative $\bar{\mathcal{C}}_1$ MLD intensities, suggesting the A_1 level lies lowest. Simultaneous lineshape fitting of the VTVH MCD and MLD spectra revealed the data could only be modeled with the A_1 level lowest at 22222.9(9) cm^{-1}, the E_2 level next at 22285.0(1.1) cm^{-1}, and the E_3 level highest at 22328.0(1.1) cm^{-1}. Observation of all three transition is supported by the change in the shape of the overlapping features with both field and temperature: a change in MCD lineshape suggested different saturation rates for the overlapping transitions and indicated a mixture of z- and xy-polarized overlapping features. Such behavior is expected of $E_1 \to (A_1 + E_2 + E_3)$ levels: $E_1 \to E_3$ transitions should be z polarized and $E_1 \to A_1/A_2/E_2$ transitions should be xy polarized.

Between the transitions to the $^3P_0$ and $^3P_2$ states are a complicated mixture of several overlapping transitions arising from the $^3P_1$ and $^1I_6$ states. The symmetry from the double group demands the $^3P_1 + {}^1I_6$

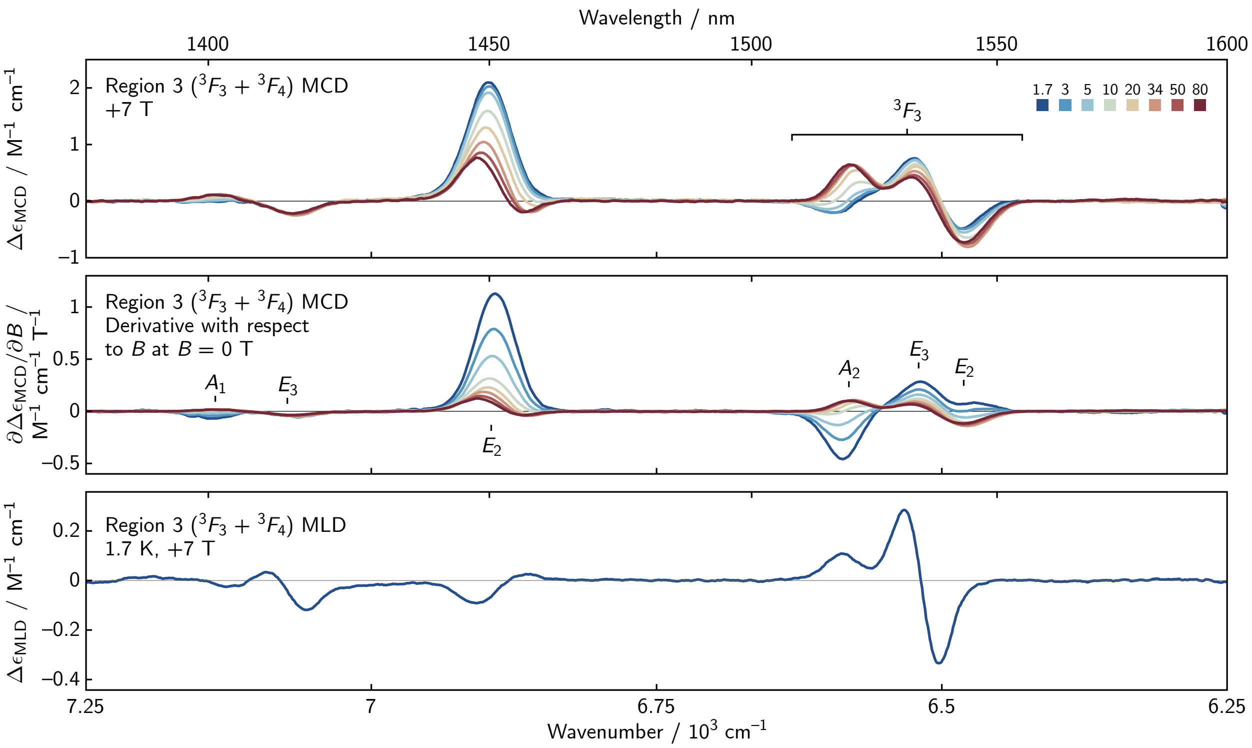

Figure S7: Cryogenic MCD and MLD spectra in a frozen 9:1 methanol-d_4/ethanol-d_6 in Region 3.

combination should split into 10 Stark levels, six of which are expected to be MCD- and/or MLD-active. With such a large number of expected transitions, we have only fit the largest MLD feature, a negative $\bar{\mathcal{C}}_1$ feature at 21140.7(3) cm^{-1}, and we have assigned it to be from the $^3P_1(E_3)$ level, as transitions to $^1I_6$ levels should generally be higher in energy and weaker in intensity due to their spin-forbidden nature.

S4.1.2 Region 2

Please see the main manuscript for an explanation of the assignments of these features.

S4.1.3 Region 3

Region 3 featured two groups of peaks (Fig. S7) corresponding to the $^3F_3$ state at lower energy and the $^3F_4$ state at higher energy. These features had an interested variation in appearance with both temperature and field, and it was important to analyze the MCD data at the lowest temperature to ensure that we were inspecting the MCD spectra characteristic of the lowest lying E_1 level of the $^3H_4$ GS. The variation in sign with field and temperature will be further discussed in Section S4.2.

The $^3F_4$ state yielded three overlapping transitions in the 6750–7250 cm^{-1} window. In the D_{4d} double group, the $^3F_4$ state should split into $A_1+E_3+E_2$ levels. The lowest energy transition shows a positive $\bar{\mathcal{C}}_0$ and $\bar{\mathcal{A}}_1$ in the MCD spectrum and a negative $\bar{\mathcal{C}}_1$ in the MLD spectrum, indicating the E_2 level. The next lowest energy transition features negative $\bar{\mathcal{C}}_0$ in the MCD spectrum and positive $\bar{\mathcal{C}}_1$ in the MLD spectrum. The sign of the $\bar{\mathcal{C}}_1$ in the MLD spectrum indicates the E_3 level. The highest energy transition displays a negative $\bar{\mathcal{C}}_0$ in the derivative at zero field of the MCD spectrum and no discernible MLD signal. Simultaneous lineshape fitting of the VTVH MCD and MLD spectra revealed the data could only be modeled with the E_2 level lowest at 6894.6(2) cm^{-1}, the E_3 level next at 7073.2(7) cm^{-1}, and the A_1 level highest at 7136.3(1.5) cm^{-1}.

The $^3F_3$ state showed three overlapping transitions in the 6250–6750 cm^{-1} window. In the D_{4d} double group, the $^3F_3$ state should split into $A_2+E_3+E_2$ levels. Simultaneous lineshape fitting of the VTVH MCD and MLD spectra revealed the data could only be modeled with the E_2 level lowest at 6480.7(3) cm^{-1}, the

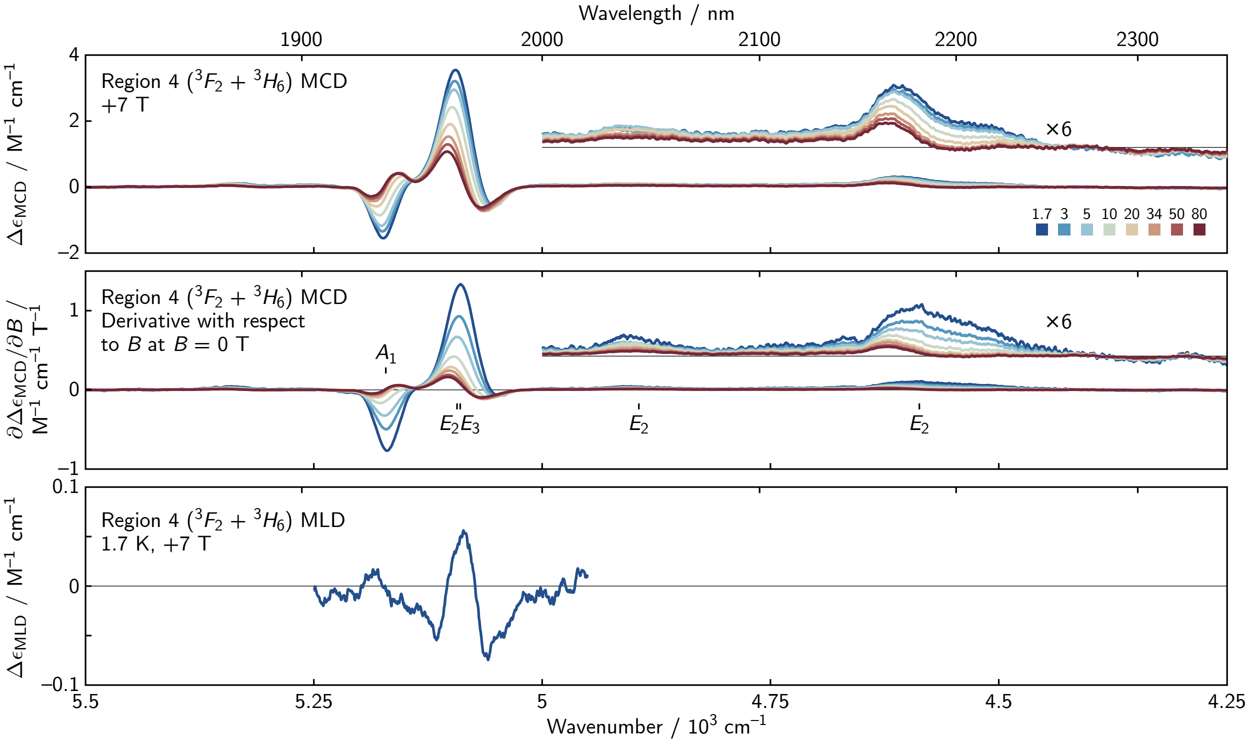

Figure S8: Cryogenic MCD and MLD spectra in a frozen 9:1 methanol-d_4/ethanol-d_6 in Region 4.

E_3 level next at 6520.2(3) cm^{-1}, and the A_2 level highest at 6581.3(2) cm^{-1}. The A_2 level was modeled well with a $\bar{\mathcal{C}}_0$ that goes from negative to positive with increasing temperature and negative $\bar{\mathcal{A}}_1$ in the MCD spectrum and positive $\bar{\mathcal{C}}_1$ in the MLD spectrum. The E_3 level was modeled well with positive $\bar{\mathcal{C}}_0$ and $\bar{\mathcal{A}}_1$ in the MCD spectrum and positive $\bar{\mathcal{C}}_1$ in the MLD spectrum. The E_2 level was modeled well with a $\bar{\mathcal{C}}_0$ that goes from positive to negative with increasing temperature and negative $\bar{\mathcal{A}}_1$ in the MCD spectrum and had no discernible features in the MLD spectrum.

S4.1.4 Region 4

In Region 4, we saw two groups of peaks (Fig. S8) assigned as the $^3H_6$ state at lower energy and the $^3F_2$ state at higher energy.

The $^3F_2$ state yielded three overlapping transitions in the 5000–5250 cm^{-1} window. In the D_{4d} double group, the $^3F_2$ state should split into $A_1 + E_3 + E_2$ levels. Simultaneous lineshape fitting of the VTVH MCD and MLD spectra revealed the data could only be modeled with the E_3 level lowest at 5090.5(2.0) cm^{-1}, the E_2 level next at 5092.4(4) cm^{-1}, and the A_1 level highest at 5171.2(2) cm^{-1}. The A_1 level was modeled well with negative $\bar{\mathcal{C}}_0$ and $\bar{\mathcal{A}}_1$ in the MCD spectrum and negative $\bar{\mathcal{C}}_1$ in the MLD spectrum. The E_2 level was modeled well with positive $\bar{\mathcal{C}}_0$ and $\bar{\mathcal{A}}_1$ in the MCD spectrum and negative $\bar{\mathcal{C}}_1$ in the MLD spectrum. The E_3 level was modeled well with negative $\bar{\mathcal{C}}_0$ in the MCD spectrum and positive $\bar{\mathcal{C}}_1$ in the MLD spectrum.

The $^3H_6$ shows very weak features occurring below 5000 cm^{-1}. We were not able to obtain usable MLD data in this region. The presence of two positive MCD features at 4894.0(7.0) and 4587.0(1.8) were tentatively assigned as E_2 transitions. The multireference calculations (Section S5) predict a splitting of 468 cm^{-1} between the two E_2 levels of the $^3H_6$ state, supporting the relatively large energetic separation between these two levels. Conducting CF parameter fitting (as in Section S4.3) without inclusion of these $^3H_6$ levels predicts these two E_2 transitions to appear at roughly these two energies, further supporting our assignment.

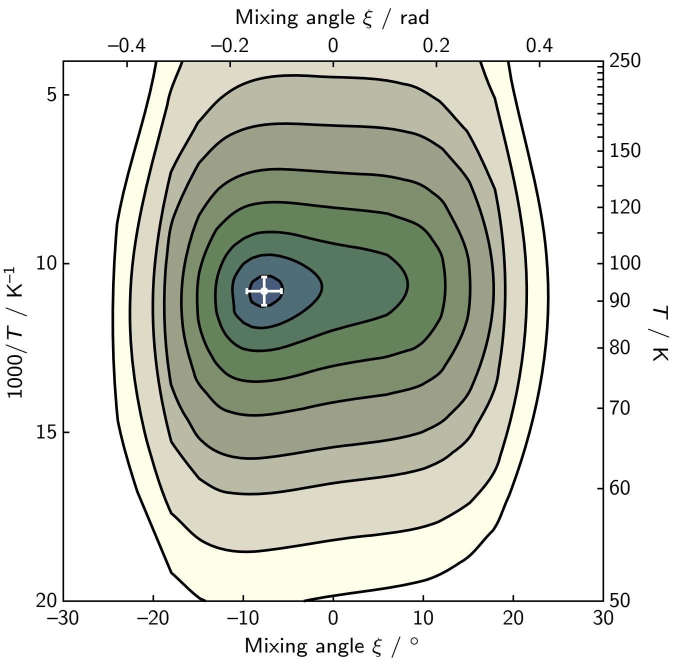

Figure S9: The best fit for the MCD saturation curves was located by minimization of a weighted least squares objective function. A contour plot of the objective function is shown here along with the minimum located at $\xi = -0.134(34)$ radians $(-7.7(1.9)^\circ)$ and $T = 92(4)$ K. The crosshair indicates the standard uncertainty of the fit.

S4.2 Calculation of Saturation Curves

Saturation curves were fit using an effective spin Hamiltonian to describe the magnetization of the GS.[16–19] As shown in Fig. 2 of the main manuscript, distortion into D_4 splits the low-lying $E_2(D_{4d})$ state into $B_1 + B_2$ levels. Magnetization will be sensitive to this splitting so we model our system using a 3×3 spin Hamiltonian that includes the lowest E and the low-lying B_1 levels within D_4 symmetry. The spin Hamiltonian takes the form

$$\begin{pmatrix} \mu_B B_z \frac{4}{5}(-3 + 4\sin^2\xi) & 0 & \mu_B(B_+)\frac{4}{5}(\frac{\sqrt{7}}{2}\cos\xi + \frac{3}{2}\sin\xi) \\ 0 & \mu_B B_z \frac{4}{5}(3 - 4\sin^2\xi) & -\mu_B(B_-)\frac{4}{5}(\frac{\sqrt{7}}{2}\cos\xi + \frac{3}{2}\sin\xi) \\ \mu_B(B_-)\frac{4}{5}(\frac{\sqrt{7}}{2}\cos\xi + \frac{3}{2}\sin\xi) & -\mu_B(B_+)\frac{4}{5}(\frac{\sqrt{7}}{2}\cos\xi + \frac{3}{2}\sin\xi) & E_{B_1} \end{pmatrix} \tag{S47}$$

where the value of 4/5 is the Landé g value for the $^3H_4$ ground state, $B_\pm = B_x \pm iB_y$, E_{B_1} is the energy of the low-lying B_1 excited state, and ξ is a mixing angle between the lowest-lying E levels ($M = \pm 3$) and the next-lowest-lying E levels ($M = \mp 1$). The mixing angle can be correlated to the geometric twisting angle through the AOM by transforming $\hat{H}_{\text{Zeeman}}$ into the eigenbasis of $\hat{H} = \hat{H}_{\text{atom}} + \hat{H}_{\text{CF}(D_4)}$ to find what twisting angle ϕ reproduces the effective g values in Eq. (S47).

This spin Hamiltonian has only two degrees of freedom: ξ and E_{B_1}. These variables were varied and Eq. (S36) was used to calculate MCD intensities. Integration was performed using a sixth order Gaussian quadrature. Weighted least-squares minimization of the residuals was performed using an objective function, and the values of this objective function are shown as a contour plot in Fig. S9. The best fit was located at $\xi = -0.134(34)$ radians and $T = 92(4)$ K. At this mixing angle, the ground state wavefunction should be 98.2(9)% $|M = \pm 3\rangle$ and 0.018(9)% $|M = \mp 1\rangle$ with an effective g_{eff} value of 2.343(28) within the lowest E level. The fits for all monitored transitions are plotted in Fig. S10. Use of the AOM to interpret the relationship between mixing angle ξ and twisting angle ϕ predicts a twist of $\phi = 37(2)^\circ$.

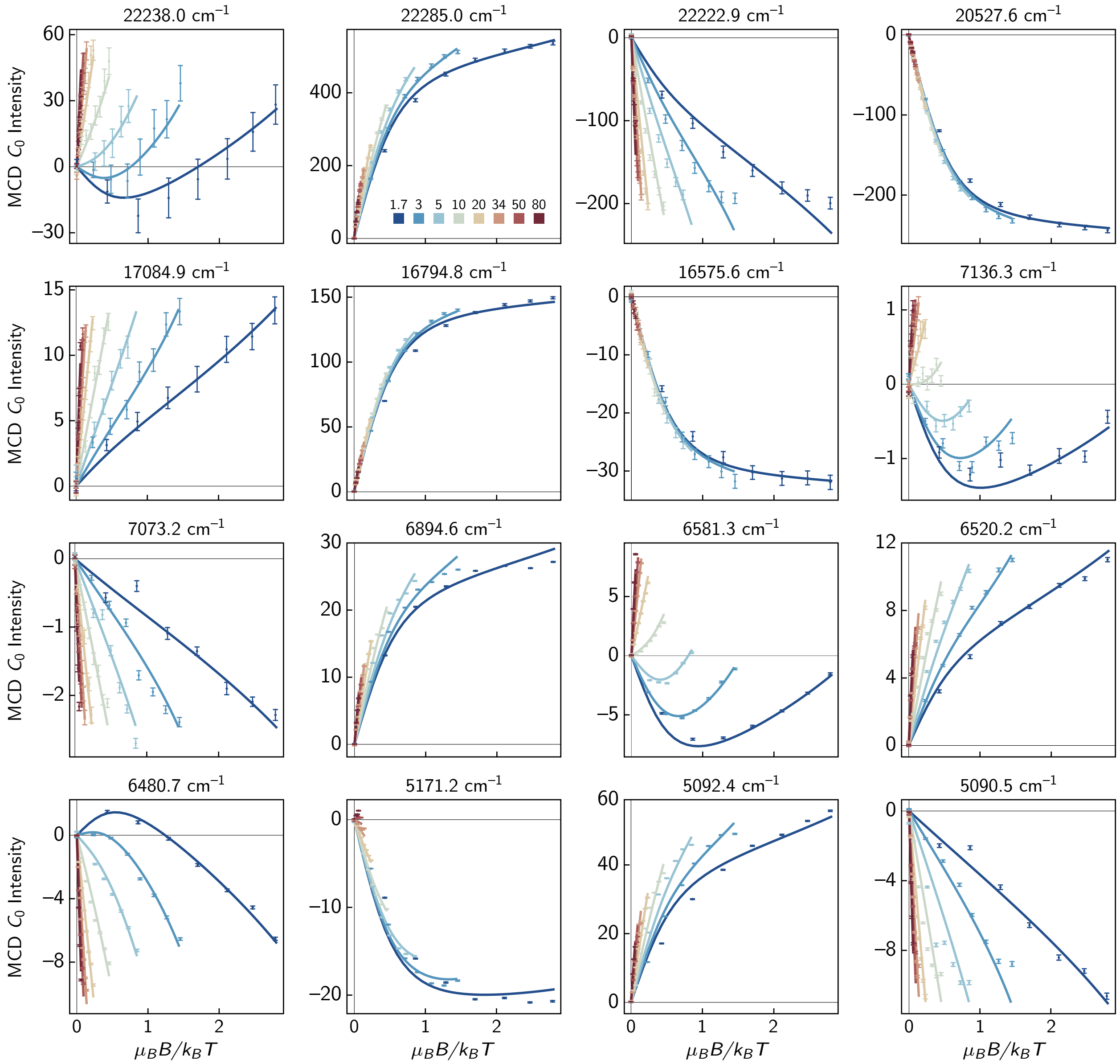

Figure S10: Saturation curves from weighted nonlinear least-squares fitting for each of the monitored transitions.

S4.3 Crystal Field Fitting

Fitting the crystal field parameters to the Hamiltonian $\hat{H} = \hat{H}_{\text{atom}} + \hat{H}_{\text{CF}}$ (see Eqs. (S1) and (S14)) presents a complicated nonlinear regression problem. Our fitting routine was approached in a few steps, starting by using only diagonal entries in the matrix Hamiltonian (in the $|SLJM\rangle$ basis) and then gradually adding in off-diagonal elements. At each step, parameter values were optimized using weighted least-squares regression. The inverse of the variance of the energy values listed in Table S5 were used as weights.

Our first step accounted for only intrastate mixing. Submatrices for each ${}^{2S+1}L_J$ state of Pr^{III} were extracted from $\hat{H}$ and the observed transition energies were fitted to the eigenvalues of these submatrices to obtain coarse energetic barycenters of each ${}^{2S+1}L_J$ state, along with initial estimates of $B_0^{(2)}$, $B_0^{(4)}$, and $B_0^{(6)}$ parameters. As an example, the ${}^1D_2$ submatrix appeared as

$$\begin{array}{c} \\ \langle +2| \\ \langle +1| \\ \langle 0| \\ \langle -1| \\ \langle -2| \end{array}
\begin{array}{c}
\begin{array}{ccccc} \quad|+2\rangle\quad & \quad|+1\rangle\quad & \quad|0\rangle\quad & \quad|-1\rangle\quad & \quad|-2\rangle\quad \end{array} \\
\begin{pmatrix}
E_{^1D_2} + \frac{22}{105}B_0^{(2)} + \frac{2}{63}B_0^{(4)} & 0 & 0 & 0 & 0 \\
0 & E_{^1D_2} - \frac{11}{105}B_0^{(2)} - \frac{8}{63}B_0^{(4)} & 0 & 0 & 0 \\
0 & 0 & E_{^1D_2} - \frac{22}{105}B_0^{(2)} + \frac{4}{21}B_0^{(4)} & 0 & 0 \\
0 & 0 & 0 & E_{^1D_2} - \frac{22}{105}B_0^{(2)} - \frac{8}{63}B_0^{(4)} & 0 \\
0 & 0 & 0 & 0 & E_{^1D_2} + \frac{22}{105}B_0^{(2)} + \frac{2}{63}B_0^{(4)}
\end{pmatrix}
\end{array}$$

where $E_{^1D_2}$ is the energetic barycenter of the ${}^1D_2$ state. Table S5 shows that we observed the $|A_1\rangle$ ($|0\rangle$) level at 16575.6(8) cm^{-1}, the $|E_3\rangle$ ($|\pm1\rangle$) levels at 17084.9(2) cm^{-1}, and the $|E_2\rangle$ ($|\pm2\rangle$) levels at 16794.8(3) cm^{-1}; thus, there are three linearly independent equations from the ${}^1D_2$ level to be used.

After this initial estimation of energetic barycenters and CF parameters, the Hamiltonian was block diagonalized into blocks corresponding to each irrep. We have only observed states of A_1, A_2, E_2, and E_3 symmetry (plus the lowest level of the GS which is E_1 symmetry); thus, all other blocks were discarded. Our second step added interstate SOC matrix elements through the $\zeta\hat{A}_{\text{SOC}}$ operator, and this mixed levels within each of these irrep blocks. Addition of this operator refined the energetic barycenters of each state, and these energies were used to calculate approximate values of Slater–Condon integrals ($F^{(2)}$, $F^{(4)}$, $F^{(6)}$) and Casimir parameters (α, β, γ). It is known that the γ configuration interaction parameter is often strongly correlated with the $F^{(2)}$ Slater–Condon integral[20] and that it generally has the strongest impact on states of differing seniority number (only the ${}^1S_0$ in a f^2 ion),[21] so we chose to fix γ to the value reported for aqueous Pr^{III}, $\gamma = 1343$ cm^{-1}.[21]

In our final step, we used the $\hat{H} = \hat{H}_{\text{atom}} = \hat{H}_{\text{CF}(D_{4d})}$ Hamiltonian to float all atomic parameters and CF parameters simultaneously. The three-electron operators were necessarily set to be zero because praseodymium(III) is a two-electron ion. Additionally, the number of $M^{(i)}$ and $P^{(j)}$ parameters was reduced by using the common technique of constraining the values to adhere to a ratio pattern: we used 1:0.56:0.31 ratios for $M^{(0)}$:$M^{(2)}$:$M^{(4)}$ and 1:0.0.5:0.1 ratios for $P^{(2)}$:$P^{(4)}$:$P^{(6)}$ as done by Carnall.[22] The final parameters and their standard uncertainties of the fit are included in Table 3, and the final calculated energy levels can be compared with the experimental one by looking at Table 2.

S4.4 Comparison with Vibrating Sample Magnetometry

The magnetic properties of microcrystalline powder of **1**·Pr (10.2 mg) was measured in a Quantum Design Dynacool Physical Properties Measurement System (PPMS) (1.8–300 K, 0–9 T) equipped with ACMS II. Susceptibility data were collected over a temperature range of 2–300 K under a 1.0 T applied field after zero-field cooling of the sample. Susceptibilities were corrected for the sample rod, the plastic sample capsule, and the intrinsic diamagnetic response of **1**·Pr as calculated from Pascal's constants.[23] Magnetic moment (B vs M) measurements were corrected for the inherent diamagnetic response of the instrument, which was independently measured through a 0–9 T B vs M experiment at 300 K without a sample loaded. The data were fit as a ${}^3H$ ion experiencing SOC with $\lambda = 744$ cm^{-1} using the `curry` function of EasySpin 6.0.10 and care was taken to convert crystal field parameters between the Wybourne formalism used in this manuscript and the Extended Stevens formalism used by EasySpin. The best-fit parameters from our MCD CF fitting were used as initial guesses for parameters in magnetometry.

S5 CASSCF/NEVPT2 Multireference Calculations

All calculations were performed using ORCA 6.0.1.[24–33]

The XYZ coordinates for the anion were taken from the refined crystallographic CIF file, and the coordinates were translated and rotated so that the tungsten atom lied at the origin and the long axis of the complex was directed along the z axis. We also used these XYZ coordinates as a starting point to build a geometry that lies strictly within the D_{4d} point group and within the D_4 point group. To do so, all 1–2 and 1–3 distances (along with some manually chosen distances with larger separations in connectivity) from the crystallographic coordinates were manually sorted into symmetry-equivalent groups within the D_{4d} point group. The XYZ coordinates were then optimized through a least-squares minimization routine till all of the monitored distances equaled the average value within their symmetry groupings. To generate the D_4 geometry twisted at 40°, one POM ligand of the D_{4d} geometry was twisted 5°. All XYZ coordinates are listed in Section S5.2.

S5.1 Calculated Energy Levels and MCD Signs

Multireference calculations require careful selection of orbitals for inclusion in the active space, and the results can vary strongly depending on which orbitals are chosen to be included. We ran our calculations with a minimal (2,7) active space using two electrons among the seven f orbitals, and we expect this to be a reasonable model for the electronic structure due to the weak metal–ligand covalency characteristic of lanthanide ions. In preparation for multireference calculations, quasi-restricted orbitals were obtained for each geometry by swapping the praseodymium(III) ion for a gadolinium(III) ion:

```
! DKH BP86 DKH-def2-SVP SARC/J UNO
%maxcore 7000
%pal nprocs 16 end
%basis
  NewGTO Mo "old-DKH-SVP" end
  NewGTO Pr "SARC-DKH-TZVP" end
  NewGTO Gd "SARC-DKH-TZVP" end
  end
*xyzfile -3 8 aligned-swap-Pr-to-Gd.xyz
```

As seen in this input file, all of these calculations used a DKH relativistic correction and relativistically-contracted basis sets: the lanthanide ion(s) used the “SARC-DKH-TZVP” basis set, all molybdenym atoms used the “old-def2-TZVP” basis set, and all other atoms used the “DKH-def2-SVP” basis set.[27,34,35] The large number of metal atoms restricted our ability to use larger basis sets in these multireference calculations.

A state-averaged CASSCF(2,7)/RI-NEVPT2 calculation was performed using the seven praseodymium f orbitals in the active space and requesting all 28 singlet and 21 triplet $4f^2$ states:

```
! DKH2 DKH-def2-SVP AutoAux MORead RI-NEVPT2
%maxcore 9000
%pal nprocs 16 end
%basis
  NewGTO Mo "old-DKH-SVP" end
  NewGTO Pr "SARC-DKH-TZVP" end
  NewGTO Gd "SARC-DKH-TZVP" end
  end
%moinp "gadolinium.qro"
%casscf
  nel 2
  norb 7
  mult 3, 1
  nroots 21, 28
  ActOrbs fOrbs
  rel
```

```
    dosoc true
    doMCD true
    NInitStates 4
    NPointsTheta 10
    NPointsPhi 10
    NPointsPsi 10
    B 1000, 70000
    Temperature 1.7, 1.7
    end
  end

* xyzfile -3 3 aligned.xyz
```

The SOC-corrected vertical excitation energies for the $f-f$ transitions of each geometry are given in Table S6.

Symmetries (irreps) of each of the 91 levels were manually determined by inspection of the direction of the transition dipole moments calculated between each level and the lowest four levels of the $^3H_4$ GS ($E_1 + E_2$ in D_{4d} and $E + B_1 + B_2$ in D_4). The D_{4d} selection rules for electric-dipole transitions require that any x, y-polarized transitions from the E_1 levels must be one of three irreps, $E_1 \otimes E_1 = A_1 + A_2 + E_2$, any z-polarized transition from the E_1 levels must be of $E_1 \otimes B_2 = E_3$ symmetry, any x, y-polarized transitions from the E_2 levels must be one of two irreps, $E_2 \otimes E_1 = E_1 + E_3$, and any z-polarized transition from the E_2 levels must be of $E_2 \otimes B_2 = E_2$ symmetry. The magnetic dipole moment ($\Gamma(R_x, R_y, R_z) = A_2 + E_3$) was sometimes useful in discriminating between irreps. Assignments in D_4 symmetry were performed in an analogous way.

The signs of MCD $\bar{\mathcal{C}}_0$ intensity can be inferred from calculation of MCD intensity at 1.7 K and 0.1 T (assumed to be in the non-saturating region). The predicted signs are shown in the $\Delta f_{\text{osc}}/f_{\text{osc}}$ column, and they agree remarkably well with the predictions in Table 1 of the main manuscript. The $\Delta f_{\text{osc}}/f_{\text{osc}}$ for the geometry from X-ray crystallography are more difficult to interpret. They were calculated at a 7 T field strength because the 12 cm^{-1} splitting between the first two levels means that a strong field must be applied before any appreciable MCD will be calculated. Additionally, this XRD structure does not perfectly lie in any point group — technically, it is in the C_1 point group. In this point group, mixing between levels seems to give nonzero f_{osc} for every level. As a consequence, there can appear to be strong $\Delta f_{\text{osc}}/f_{\text{osc}}$ values but the small f_{osc} value means the transitions are unlikely to be observed in practice.

Table S6: Calculated transition energies (cm^{-1})

Identification		Perfect D_{4d}			Perfect D_4 (40° Twist)			XRD Geometry	
$^{2S+1}L_J$	#	Γ	E	$\Delta f_{osc}/f_{osc}$	Γ	E	$\Delta f_{osc}/f_{osc}$	E	$\Delta f_{osc}/f_{osc}$
$^3H_4$	0	E_1	0		E	0		0	
	1		0	0.00		0	0.00	12	0.00
	2	E_2	97	0.68	B_1	50	0.93	64	0.39
	3		97	0.71	B_2	165	0.56	167	0.57
	4	E_3	220	0.00	E	209	0.00	197	−0.11
	5		220	0.00		209	0.00	213	−0.63
	6	A_1	246	−0.85	A_1	227	−0.72	226	−0.15
	7	B_2	483	0.00	A_2	480	−0.69	489	−0.49
	8	B_1	486	0.00	A_1	486	−0.71	494	0.69
$^3H_5$	9	E_1	1832	0.00	E	1840	0.31	1838	0.25
	10		1832	0.00		1840	0.00	1849	0.55
	11	B_1	1910	0.00	B_2	1857	0.56	1863	0.39
	12	B_2	1912	0.00	A_1	1911	−0.72	1916	0.62
	13	E_2	1914	0.58	A_2	1914	−0.72	1924	−0.43
	14		1914	0.78	B_1	1962	0.92	1941	−0.29
	15	E_3	1966	0.01	E	1964	0.01	1968	0.68
	16		1966	0.01		1964	0.18	1977	0.49
	17	A_2	2011	−0.65	A_2	1987	−0.84	1989	0.52
	18	E_1	2263	0.00	E	2261	0.01	2270	0.23
	19		2263	0.00		2261	0.00	2270	0.31
$^3H_6$	20	B_2	3661	0.00	A_2	3677	0.00	3675	0.02
	21	B_1	3664	0.00	A_1	3681	0.56	3686	0.72
	22	E_1	3750	0.00	E	3733	−0.72	3728	0.11
	23		3750	0.58		3733	−0.72	3747	−0.41
	24	E_1	3777	0.78	B_1	3758	0.92	3768	0.59
	25		3777	0.01	E	3774	0.01	3777	0.69
	26	E_2	3832	0.01		3774	0.18	3795	0.06
	27		3832	−0.65	A_1	3858	−0.84	3852	−0.60
	28	E_3	3863	0.00	E	3873	0.01	3866	−0.42
	29		3863	0.00		3873	0.00	3871	0.48
	30	A_1	3866	0.00	B_2	3881	0.00	3882	0.83
	31	E_2	4300	0.00	B_2	4298	0.56	4307	0.44
	32		4300	0.00	B_1	4298	−0.72	4307	0.36
$^3F_2$	33	E_3	5316	0.00	E	5315	0.00	5314	−0.15
	34		5316	0.00		5315	0.00	5324	−0.17
	35	E_2	5357	0.58	B_1	5344	0.93	5346	0.36
	36		5357	0.75	B_2	5369	0.56	5370	0.59
	37	A_1	5489	−0.85	A_1	5489	−0.72	5494	−0.49
$^3F_3$	38	E_1	6455	0.07	B_1	6434	0.93	6435	0.20
	39		6455	0.71	E	6449	−0.05	6450	−0.41
	40	E_2	6456	0.57		6449	0.00	6454	0.58
	41		6456	0.75	B_2	6477	0.56	6480	0.37
	42	E_3	6513	0.00	E	6517	0.00	6520	0.06
	43		6513	0.00		6517	0.00	6522	0.10
	44	A_2	6582	−0.59	A_2	6582	−0.68	6586	−0.23
$^3F_4$	45	E_1	6715	0.00	E	6716	0.15	6719	0.49
	46		6715	0.00		6716	0.00	6722	0.54
	47	E_2	6733	0.19	B_2	6728	0.56	6731	0.20
	48		6733	0.82	B_1	6738	0.93	6741	0.85
	49	B_1	6899	0.00	A_1	6883	−0.66	6885	−0.28
	50	E_3	6906	−0.01	E	6904	−0.01	6903	−0.38
	51		6906	0.00		6904	0.00	6907	−0.26
	52	B_2	6912	0.00	A_2	6911	−0.68	6915	0.13
	53	A_1	6954	−0.85	A_1	6963	−0.72	6966	−0.11
$^1G_4$	54	E_2	9103	0.36	B_2	9092	0.56	9092	0.36
	55		9103	0.81	B_1	9111	0.93	9114	0.69
	56	E_1	9135	0.00	E	9133	0.00	9138	0.30
	57		9135	0.00		9133	0.00	9141	0.62
	58	E_3	9317	−0.01	E	9315	−0.01	9314	−0.38
	59		9317	−0.01		9315	−0.01	9317	−0.48
	60	B_1	9385	0.00	A_1	9335	−0.71	9337	−0.03
	61	B_2	9402	0.00	A_2	9401	−0.68	9406	−0.03
	62	A_1	9431	−0.85	A_1	9475	−0.72	9489	−0.21
$^1D_2$	63	A_1	18445	−0.85	A_1	18442	−0.72	18480	0.36
	64	E_2	18593	0.61	B_2	18551	0.56	18520	0.39
	65		18593	0.77	B_1	18632	0.93	18628	0.59
	66	E_3	18981	0.00	E	18979	0.00	18982	0.10
	67		18981	0.00		18979	0.00	18984	0.11
$^3P_0$	68	A_1	23268	−0.85	A_1	23268	−0.72	23268	−0.49
$^3P_1$	69	A_2	23680	−0.60	A_2	23680	−0.68	23678	−0.41
	70	E_3	23806	0.00	E	23806	0.00	23791	0.10
	71		23806	0.00		23806	0.00	23823	0.30
$^3P_2$ $^1I_6$	72	A_1	24108	−0.85	A_1	24104	−0.73	24096	−0.25
	73	E_3	24184	0.00	E	24181	−0.01	24145	0.13
	74		24184	0.00		24181	0.00	24194	−0.35
	75	E_2	24315	0.59	B_2	24278	0.56	24277	0.46
	76		24315	0.78	B_1	24347	0.93	24349	0.52
	77	E_2	24593	0.78	B_2	24587	0.55	24599	0.20
	78		24593	0.00	B_1	24588	0.94	24601	0.46
	79	E_1	24618	0.00	E	24621	−0.01	24615	0.58
	80		24618	0.00		24621	0.00	24624	0.22
	81	E_2	24667	0.43	B_2	24649	0.56	24647	0.39
	82		24667	0.80	B_1	24692	0.93	24690	0.60
	83	A_1	24812	−0.85	A_1	24811	−0.73	24818	−0.52
	84	E_3	24829	0.00	E	24825	0.00	24824	0.28
	85		24829	0.01		24825	0.01	24835	0.47
	86	B_1	24881	0.00	A_1	24886	0.00	24890	0.27
	87	B_2	24911	0.00	A_2	24912	−0.69	24914	0.12
	88	E_1	24976	0.00	E	24985	0.00	24995	0.10
	89		24976	0.00		24985	0.00	24996	0.15
$^1S_0$	90	A_1	50555	−0.85	A_1	50555	−0.72	50554	−0.49

S5.2 XYZ Coordinates

Coordinates from XRD Study

Pr	0.00000	0.00000	0.00000
O	0.21328	-0.06427	3.56971
Mo	0.34640	-0.10654	5.68971
N	0.42497	-0.15578	7.45751
O	0.45803	-0.21620	8.65417
O	-1.39692	1.38242	1.56512
Mo	-1.49148	1.50269	3.30268
O	-2.41544	-0.13502	3.60165
O	-2.69665	2.65171	3.63462
O	-1.09724	1.25905	5.50280
C	-1.98162	1.57665	6.53582
H	-2.74151	0.95828	6.51814
H	-2.30533	2.49480	6.41835
H	-1.51942	1.50184	7.39683
O	-1.31492	-1.46292	1.50012
Mo	-1.38921	-1.72557	3.23010
O	0.25722	-2.67456	3.37531
O	-2.54328	-2.94588	3.47215
O	-1.05160	-1.52563	5.43537
C	-2.18861	-1.67064	6.31147
H	-2.59368	-2.55352	6.17763
H	-2.84809	-0.97498	6.10985
H	-1.89656	-1.58418	7.24323
O	1.53970	-1.46518	1.31581
Mo	1.84721	-1.64895	3.02764
O	2.79730	-0.01135	3.24215
O	3.04900	-2.83716	3.16386
O	1.74905	-1.46983	5.26675
C	2.24054	-2.50380	6.09774
H	1.68859	-3.30592	5.98135
H	2.20530	-2.21611	7.03321
H	3.16706	-2.70717	5.85212
O	1.44877	1.42920	1.37768
Mo	1.74343	1.59328	3.08936
O	0.12993	2.53940	3.49293
O	2.91700	2.79948	3.23894
O	1.71467	1.32141	5.31768
C	2.98354	1.37310	6.04826
H	2.80778	1.32052	7.01051
H	3.44166	2.21566	5.84519
H	3.54728	0.62033	5.77604
O	-0.03951	-0.01465	-3.52549
Mo	-0.05199	-0.00158	-5.65795
N	0.03518	-0.03810	-7.41906
O	0.16059	-0.07891	-8.61809
O	-2.03799	0.14062	-1.37963
Mo	-2.30401	0.14727	-3.11021
O	-1.99460	-1.72627	-3.37342
O	-3.97423	0.24774	-3.35129
O	-2.02583	0.08687	-5.36316
C	-2.98798	-0.53684	-6.20947

H	-3.02625	-1.49534	-6.00833
H	-2.73062	-0.40907	-7.14574
H	-3.86808	-0.13508	-6.05386
O	-0.15391	-2.01538	-1.38874
Mo	-0.19725	-2.30293	-3.11227
O	1.66615	-1.98439	-3.38640
O	-0.31150	-3.99455	-3.30144
O	-0.17227	-1.99236	-5.33806
C	0.41763	-2.96414	-6.18765
H	-0.02714	-3.82751	-6.05605
H	1.37000	-3.05151	-5.97306
H	0.31826	-2.68517	-7.12229
O	2.01882	-0.15186	-1.40046
Mo	2.25493	-0.18663	-3.13652
O	1.93224	1.69282	-3.39278
O	3.92321	-0.32072	-3.38116
O	1.92084	-0.15890	-5.34094
C	2.92286	-0.64538	-6.25970
H	3.77042	-0.18034	-6.09028
H	2.63362	-0.46421	-7.18029
H	3.04333	-1.60523	-6.13566
O	0.10578	2.03814	-1.38800
Mo	0.14082	2.27914	-3.12602
O	-1.72558	1.94234	-3.42373
O	0.25395	3.95274	-3.36609
O	0.12780	1.97527	-5.36416
C	0.94315	2.83878	-6.16543
H	1.87807	2.76036	-5.88265
H	0.64503	3.76628	-6.05227
H	0.86322	2.58287	-7.10726

Perfect D_{4d} Geometry

Pr	0.00000	0.00000	0.00000
O	0.00000	0.00000	3.55156
Mo	0.00000	0.00000	5.67987
N	0.00000	0.00000	7.44633
O	0.00000	0.00000	8.64826
O	2.02869	0.00000	1.41573
Mo	2.29145	0.00156	3.14527
O	1.84083	1.84312	3.41475
O	3.97212	0.00246	3.36721
O	1.98195	-0.00295	5.37248
H	2.42311	-0.84756	5.80522
O	0.00000	2.02869	1.41573
Mo	-0.00156	2.29145	3.14527
O	-1.84312	1.84083	3.41475
O	-0.00246	3.97212	3.36721
O	0.00295	1.98195	5.37248
H	0.84756	2.42311	5.80522
O	-2.02869	0.00000	1.41573
Mo	-2.29145	-0.00156	3.14527
O	-1.84083	-1.84312	3.41475
O	-3.97212	-0.00246	3.36721
O	-1.98195	0.00295	5.37248

H	-2.42311	0.84756	5.80522
O	0.00000	-2.02869	1.41573
Mo	0.00156	-2.29145	3.14527
O	1.84312	-1.84083	3.41475
O	0.00246	-3.97212	3.36721
O	-0.00295	-1.98195	5.37248
H	-0.84756	-2.42311	5.80522
O	0.00000	0.00000	-3.55156
Mo	0.00000	0.00000	-5.67987
N	0.00000	0.00000	-7.44633
O	0.00000	0.00000	-8.64826
O	1.43450	1.43450	-1.41573
Mo	1.62140	1.61920	-3.14527
O	2.60495	-0.00162	-3.41475
O	2.81046	2.80697	-3.36721
O	1.39937	1.40354	-5.37248
H	1.11408	2.31271	-5.80522
O	1.43450	-1.43450	-1.41573
Mo	1.61920	-1.62140	-3.14527
O	-0.00162	-2.60495	-3.41475
O	2.80697	-2.81046	-3.36721
O	1.40354	-1.39937	-5.37248
H	2.31271	-1.11408	-5.80522
O	-1.43450	-1.43450	-1.41573
Mo	-1.62140	-1.61920	-3.14527
O	-2.60495	0.00162	-3.41475
O	-2.81046	-2.80697	-3.36721
O	-1.39937	-1.40354	-5.37248
H	-1.11408	-2.31271	-5.80522
O	-1.43450	1.43450	-1.41573
Mo	-1.61920	1.62140	-3.14527
O	0.00162	2.60495	-3.41475
O	-2.80697	2.81046	-3.36721
O	-1.40354	1.39937	-5.37248
H	-2.31271	1.11408	-5.80522

Perfect D_4 Geometry (40° Twist Angle)

Pr	0.00000	0.00000	0.00000
O	0.00000	0.00000	3.55156
Mo	0.00000	0.00000	5.67987
N	0.00000	0.00000	7.44633
O	0.00000	0.00000	8.64826
O	1.90635	0.69385	1.41573
Mo	2.15273	0.78519	3.14527
O	1.09943	2.36157	3.41475
O	3.73173	1.36086	3.36721
O	1.86344	0.67509	5.37248
H	2.56686	0.03231	5.80522
O	-0.69385	1.90635	1.41573
Mo	-0.78519	2.15273	3.14527
O	-2.36157	1.09943	3.41475
O	-1.36086	3.73173	3.36721
O	-0.67509	1.86344	5.37248
H	-0.03231	2.56686	5.80522

O	-1.90635	-0.69385	1.41573
Mo	-2.15273	-0.78519	3.14527
O	-1.09943	-2.36157	3.41475
O	-3.73173	-1.36086	3.36721
O	-1.86344	-0.67509	5.37248
H	-2.56686	-0.03231	5.80522
O	0.69385	-1.90635	1.41573
Mo	0.78519	-2.15273	3.14527
O	2.36157	-1.09943	3.41475
O	1.36086	-3.73173	3.36721
O	0.67509	-1.86344	5.37248
H	0.03231	-2.56686	5.80522
O	0.00000	0.00000	-3.55156
Mo	0.00000	0.00000	-5.67987
N	0.00000	0.00000	-7.44633
O	0.00000	0.00000	-8.64826
O	0.69385	1.90635	-1.41573
Mo	0.78519	2.15273	-3.14527
O	2.36157	1.09943	-3.41475
O	1.36086	3.73173	-3.36721
O	0.67509	1.86344	-5.37248
H	0.03231	2.56686	-5.80522
O	1.90635	-0.69385	-1.41573
Mo	2.15273	-0.78519	-3.14527
O	1.09943	-2.36157	-3.41475
O	3.73173	-1.36086	-3.36721
O	1.86344	-0.67509	-5.37248
H	2.56686	-0.03231	-5.80522
O	-0.69385	-1.90635	-1.41573
Mo	-0.78519	-2.15273	-3.14527
O	-2.36157	-1.09943	-3.41475
O	-1.36086	-3.73173	-3.36721
O	-0.67509	-1.86344	-5.37248
H	-0.03231	-2.56686	-5.80522
O	-1.90635	0.69385	-1.41573
Mo	-2.15273	0.78519	-3.14527
O	-1.09943	2.36157	-3.41475
O	-3.73173	1.36086	-3.36721
O	-1.86344	0.67509	-5.37248
H	-2.56686	0.03231	-5.80522

S6 Appendices

S6.1 First/Second Derivative Lineshapes from the BO–FC–RS Approximation

The Born–Oppenheimer (BO), Franck–Condon (FC), and Rigid Shift (RS) approximations are commonly used in the modeling/interpretation of magneto-optical data. Using BO states with the FC approximation allows us to separate and discard the vibrational components of the molecular wavefunction. The transitions seen in **1**·Pr do not show evidence for vibronic complications, so this is a reasonable treatment for our data. The RS approximation assumes that for a transition $A \to J$, all transitions between sublevels $a \in A$ and $j \in J$ share the same lineshape. Because the energies of sublevels a and j vary with applied field, their contribution $f_{a\to j}(E)$ will appear slightly shifted to higher/lower energy from the average $A \to J$ transition energy; thus, the RS approximation can be expressed $f_{a\to j}(E) \approx f(E - \Delta E_{a\to j})$ for a shared lineshape function f where $\Delta E_{a\to j}$ is the energy shift from the average energy of the $A \to J$ transition. Taylor expansion about $\Delta E_{a\to j} = 0$ yields

$$f(E - \Delta E_{a\to j}) \approx f(E) - \Delta E_{a\to j} f'(E) + \frac{1}{2}\Delta E_{a\to j}^2 f''(E) + \cdots \tag{S48}$$

This Taylor series expansion of the lineshape is the source of the first- and second-derivative terms in the traditional Faraday expressions for MCD and MLD intensity.

Traditional discussions of MCD (and MLD) further subdivide these contributions according to how they vary with temperature under non-saturating conditions. The monosignate (zeroth-order derivative) signal in MCD is parsed into a temperature-independent B_0 component that arises from *inter*-state mixing and a temperature-dependent C_0/k_BT component that arises from *intra*-state mixing. The bisignate (first-order derivative) signal in MCD comes from the field-driven splitting of degenerate ground and/or excited state levels and is called A_1. We direct the interested reader to Mason[36] for further explanation of the origins of the MCD and MLD Faraday terms.

S6.2 Taylor Series Expansion of Intensity Equations

The MCD and MLD intensity expressions before orientational averaging in θ and ϕ, Eqs. (S35) and (S38), will be expanded as series in applied magnetic field B. The MCD expression requires expansion to first order and the MLD expression requires expansion to second order to obtain terms that persist upon orientational averaging.

Beginning with the MCD expression, we set $N_j = 0$, use Eq. (S48) (to first order) to treat lineshape, and rearrange to obtain

$$\begin{aligned}\frac{\Delta\epsilon_{\text{MCD}}}{E} &= i\gamma\vec{z}_{\text{lab}} \cdot \sum_{aj} N_a \langle a|\hat{\vec{m}}|j\rangle \times \langle j|\hat{\vec{m}}|a\rangle \left(f(E) - \Delta E_{a\to j} f'(E)\right) \\ &= i\gamma\vec{z}_{\text{lab}} \cdot \sum_{aj} N_a \langle a|\hat{\vec{m}}|j\rangle \times \langle j|\hat{\vec{m}}|a\rangle f(E) - i\gamma\vec{z}_{\text{lab}} \cdot \sum_{aj} N_a \Delta E_{a\to j} \langle a|\hat{\vec{m}}|j\rangle \times \langle j|\hat{\vec{m}}|a\rangle f'(E)\end{aligned} \tag{S49}$$

These expressions implicitly use eigenstates a and j of the Hamiltonian, and we can use resolution of identity¶

¶This resolution of identity operation assumes that it is possible to express the a, j levels accurately in this M_A, M_J basis. Furthermore, in D_{4d}, it is more appropriate to use a $\Gamma_A\gamma_A, \Gamma_J\gamma_J$ basis but we have used a simpler M_A, M_J notation here.

to recast them in more convenient M levels:

$$\begin{aligned}\frac{\Delta\epsilon_{\mathrm{MCD}}}{E} &= i\gamma\vec{z}_{\mathrm{lab}}\cdot\sum_{aj}\sum_{\substack{M_A M'_A\\ M_J M'_J}} N_a\langle a|M_A\rangle\langle M_A|\hat{\vec{m}}|M_J\rangle\langle M_J|j\rangle\times\langle j|M'_J\rangle\langle M'_J|\hat{\vec{m}}|M'_A\rangle\langle M'_A|a\rangle f(E)\\
&\quad - i\gamma\vec{z}_{\mathrm{lab}}\cdot\sum_{aj}\sum_{\substack{M_A M'_A\\ M_J M'_J}} N_a\Delta E_{a\to j}\langle a|M_A\rangle\langle M_A|\hat{\vec{m}}|M_J\rangle\langle M_J|j\rangle\times\langle j|M'_J\rangle\langle M'_J|\hat{\vec{m}}|M'_A\rangle\langle M'_A|a\rangle f'(E)\\
&= i\gamma\vec{z}_{\mathrm{lab}}\cdot\sum_{\substack{M_A M'_A\\ M_J M'_J}}\left(\sum_{aj}\langle M_J|j\rangle\langle M'_A|a\rangle N_a\langle a|M_A\rangle\langle j|M'_J\rangle\right)\langle M_A|\hat{\vec{m}}|M_J\rangle\times\langle M'_J|\hat{\vec{m}}|M'_A\rangle f(E)\\
&\quad - i\gamma\vec{z}_{\mathrm{lab}}\cdot\sum_{\substack{M_A M'_A\\ M_J M'_J}}\left(\sum_{aj}\langle M_J|j\rangle\langle M'_A|a\rangle N_a\Delta E_{a\to j}\langle a|M_A\rangle\langle j|M'_J\rangle\right)\langle M_A|\hat{\vec{m}}|M_J\rangle\times\langle M'_J|\hat{\vec{m}}|M'_A\rangle f'(E)\end{aligned} \tag{S50}$$

Both N_a and $\Delta E_{a\to j}$ can be thought of as matrices that are diagonal in the eigenstates a, j of the Hamiltonian. Note that it is only the parenthetical portions of Eq. (S50) that can vary with field strength. The former parenthetical expression can be rewritten

$$\sum_{aj}\langle M_J|j\rangle\langle M'_A|a\rangle N_a\langle a|M_A\rangle\langle j|M'_J\rangle = \delta_{M_J M'_J}\langle M'_A|\hat{\rho}_A|M_A\rangle \tag{S51}$$

using the GS density matrix $\hat{\rho}_A$. The latter parenthetical expression can be rewritten

$$\sum_{aj}\langle M_J|j\rangle\langle M'_A|a\rangle N_a\Delta E_{a\to j}\langle a|M_A\rangle\langle j|M'_J\rangle = \langle M_J|\langle M'_A|(\hat{H}_J-\hat{H}_A)\hat{\rho}_A|M_A\rangle|M'_J\rangle \tag{S52}$$

where $\hat{H}_A$ and $\hat{H}_J$ are *traceless* Hamiltonian submatrices corresponding to the A and J states, respectively. The MCD expression in this basis is

$$\begin{aligned}\frac{\Delta\epsilon_{\mathrm{MCD}}}{E} &= i\gamma\vec{z}_{\mathrm{lab}}\cdot\sum_{\substack{M_A M'_A\\ M_J}}\langle M'_A|\hat{\rho}_A|M_A\rangle\langle M_A|\hat{\vec{m}}|M_J\rangle\times\langle M_J|\hat{\vec{m}}|M'_A\rangle f(E)\\
&\quad - i\gamma\vec{z}_{\mathrm{lab}}\cdot\sum_{\substack{M_A M'_A\\ M_J M'_J}}\langle M_J|\langle M'_A|(\hat{H}_J-\hat{H}_A)\hat{\rho}_A|M_A\rangle|M'_J\rangle\langle M_A|\hat{\vec{m}}|M_J\rangle\times\langle M'_J|\hat{\vec{m}}|M'_A\rangle f'(E)\end{aligned} \tag{S53}$$

Repeating this treatment for the MLD expression (using Eq. (S48) to second order) we obtain

$$\begin{aligned}\left\langle\frac{\Delta\epsilon_{\mathrm{MLD}}}{E}\right\rangle_{\psi\text{ only}} &= \gamma\sum_{\substack{M_A M'_A\\ M_J}}\langle M'_A|\hat{\rho}_A|M_A\rangle\langle M_A|\hat{\vec{m}}|M_J\rangle\cdot\left(\frac{3}{2}\vec{z}_{\mathrm{lab}}\vec{z}_{\mathrm{lab}}^{\mathsf{T}}-\frac{1}{2}\right)\cdot\langle M_J|\hat{\vec{m}}|M'_A\rangle f(E)\\
&- \gamma\sum_{\substack{M_A M'_A\\ M_J M'_J}}\langle M_J|\langle M'_A|(\hat{H}_J-\hat{H}_A)\hat{\rho}_A|M_A\rangle|M'_J\rangle\langle M_A|\hat{\vec{m}}|M_J\rangle\cdot\left(\frac{3}{2}\vec{z}_{\mathrm{lab}}\vec{z}_{\mathrm{lab}}^{\mathsf{T}}-\frac{1}{2}\right)\cdot\langle M'_J|\hat{\vec{m}}|M'_A\rangle f'(E)\\
&+ \frac{1}{2}\gamma\sum_{\substack{M_A M'_A\\ M_J M'_J}}\langle M_J|\langle M'_A|(\hat{H}_J-\hat{H}_A)^2\hat{\rho}_A|M_A\rangle|M'_J\rangle\langle M_A|\hat{\vec{m}}|M_J\rangle\cdot\left(\frac{3}{2}\vec{z}_{\mathrm{lab}}\vec{z}_{\mathrm{lab}}^{\mathsf{T}}-\frac{1}{2}\right)\cdot\langle M'_J|\hat{\vec{m}}|M'_A\rangle f''(E)\end{aligned} \tag{S54}$$

In both the MCD and MLD equations here, only the density matrix $\hat{\rho}_A$ and the traceless $\hat{H}_{A/J}$ submatrices will vary with field strength. At thermal equilibrium (a Boltzmann distribution), the GS density matrix is

$$\hat{\rho}_A = \frac{\exp(-\hat{H}_A/k_BT)}{\mathrm{Tr}\,\exp(-\hat{H}_A/k_BT)}, \tag{S55}$$

and this will evaluate differently depending on the form of $\hat{H}_A$.

So far, we have not placed many restrictions on the identities or degeneracies of A or J states but we can establish predicted signs for MCD or MLD signals if A and J each correspond to a single irrep within a state. For a $4f^2$ D_{4d} ion, its states will correspond to an irrep of the double group, leaving only three possibilities for the form of $\hat{H}_A$ and $\hat{H}_J$:

$$\hat{H}_A, \hat{H}_J = \begin{cases} \begin{pmatrix} \mu_B B_z g_{\text{eff}} & 0 \\ 0 & -\mu_B B_z g_{\text{eff}} \end{pmatrix} & \text{for doubly degenerate } E_1,\ E_2, \text{ or } E_3 \text{ levels} \\ \begin{pmatrix} -\Delta/2 & \mu_B B_z g_{\text{eff}} \\ \mu_B B_z g_{\text{eff}} & \Delta/2 \end{pmatrix} & \text{for quasi-degenerate } B_1 + B_2 \text{ levels} \\ (0) & \text{for singly degenerate } A_1 \text{ or } A_2 \text{ levels} \end{cases} \tag{S56}$$

where we write the components of the magnetic field as $\vec{B} = (B_x, B_y, B_z)$. The effective g value will take the value $g_{\text{eff}} = \pm g_{\text{Landé}}|M|$ if M remains a good quantum number, where $g_{\text{Landé}}$ is the Landé g value for the ${}^{2S+1}L_J$ state. The value of g_{eff} is positive if the positive M transforms like component 1 of the irrep and negative otherwise (using Eqs. (S25)–(S27)). The quasi-degenerate $B_1 + B_2$ levels use a positive g_{eff} and the Δ is the splitting between these levels. Use Table S4 for convenience. Note that none of these matrices are sensitive to B_x or B_y and this is a consequence of the fact that $\hat{J}_x$ and $\hat{J}_y$ operators can only have matrix elements between levels differing by $\Delta M = \pm 1$, something that is not possible for (quasi-)degenerate $\pm M$ states of D_{4d}-symmetric even-electron systems.‖ For each of these $\hat{H}_A$ possibilities, the Taylor expansion of $\hat{\rho}$ to second order is

$$\hat{\rho}_A = \begin{cases} \begin{pmatrix} \frac{1}{2} - \frac{1}{2}\frac{\mu_B B_z g_{\text{eff}}}{k_B T} & 0 \\ 0 & \frac{1}{2} + \frac{1}{2}\frac{\mu_B B_z g_{\text{eff}}}{k_B T} \end{pmatrix} \quad \text{for } E_1,\ E_2, \text{ or } E_3 \text{ levels} \\ \begin{pmatrix} \frac{e^{\Delta/k_B T}}{1+e^{\Delta/k_B T}} + \frac{\mu_B^2 B_z^2 g_{\text{eff}}^2}{\Delta^2}\frac{1+2\frac{\Delta}{k_B T}e^{\Delta/k_B T} - e^{2\Delta/k_B T}}{1+e^{\Delta/k_B T}} & -\frac{\mu_B B_z g_{\text{eff}}}{\Delta}\tanh(\Delta/2k_B T) \\ -\frac{\mu_B B_z g_{\text{eff}}}{\Delta}\tanh(\Delta/2k_B T) & \frac{1}{1+e^{\Delta/k_B T}} - \frac{\mu_B^2 B_z^2 g_{\text{eff}}^2}{\Delta^2}\frac{1+2\frac{\Delta}{k_B T}e^{\Delta/k_B T} - e^{2\Delta/k_B T}}{1+e^{\Delta/k_B T}} \end{pmatrix} \\ \qquad \text{for } B_1 + B_2 \text{ levels} \\ (1) \quad \text{for } A_1 \text{ or } A_2 \text{ levels} \end{cases} \tag{S57}$$

‖In order for $\Delta M = \pm 1$ between degenerate $\pm M$ levels, we must have $M - (-M) = 1$ and this is only satisfied by $M = 1/2$, something impossible for even-electron systems. Thus, even-electron systems with degenerate $\pm M$ levels *cannot* have an x/y response in an axial environment when M remains a good quantum number. If axiality breaks or if M ceases to be a good quantum number then this rule no longer applies.

References

[1] Klemperer, W. G. *Inorganic Syntheses*; John Wiley & Sons, Ltd, 1990; pp 74–85.

[2] She, S.; Gao, C.; Chen, K.; Bayaguud, A.; Huang, Y.; Wang, B.-W.; Gao, S.; Wei, Y. *Inorg. Chem.* **2018**, *57*, 963–969.

[3] Zhang, Y.; Jia, H.; Zhang, J.; Zhu, S.; Chen, K.; Wei, Y. *Inorg. Chem. Commun.* **2016**, *70*, 177–180.

[4] Peterson, J.; Pearce, L. L.; Bominaar, E. L. *J. Am. Chem. Soc.* **1999**, *121*, 5972–5980.

[5] Liu, G. In *Spectroscopic Properties of Rare Earths in Optical Materials*; Hull, R., Parisi, J., Osgood, R. M., Warlimont, H., Liu, G., Jacquier, B., Eds.; Springer Series in Materials Science; Springer: Berlin, Heidelberg, 2005; pp 1–94.

[6] Liu, G.; Jacquier, B. *Spectroscopic Properties of the Rare Earths in Optical Materials*; Materials Science 83; Springer-Verlag, 2005.

[7] Racah, G. *Phys. Rev.* **1949**, *76*, 1352–1365.

[8] Judd, B. R. *Rep. Prog. Phys.* **1985**, *48*, 907.

[9] Butler, P. H. *Point Group Symmetry Applications*; Springer US: Boston, MA, 1981.

[10] Judd, B. R.; Crosswhite, H. M.; Crosswhite, H. *Phys. Rev.* **1968**, *169*, 130–138.

[11] Altmann, S. L.; Herzig, P. *Point-Group Theory Tables*, 2nd ed.; University of Vienna PHAIDRA: Wien, 2011.

[12] Suta, M.; Cimpoesu, F.; Urland, W. *Coord. Chem. Rev.* **2021**, *441*, 213981.

[13] Nielson, C. W.; Koster, G. F. *Spectroscopic Coefficients for the p^n, d^n, and f^n Configurations*; MIT Press: Cambridge, MA, 1963.

[14] Piepho, S. B.; Schatz, P. N. *Group Theory in Spectroscopy with Applications to Magnetic Circular Dichroism*; John Wiley & Sons: New York, 1983.

[15] Bominaar, E. L.; Achim, C.; Peterson, J. *J. Chem. Phys.* **1998**, *109*, 942–950.

[16] Oganesyan, V. S.; George, S. J.; Cheesman, M. R.; Thomson, A. J. *J. Chem. Phys.* **1999**, *110*, 762–777.

[17] Oganesyan, V. S.; Thomson, A. J. *J. Chem. Phys.* **2000**, *113*, 5003–5017.

[18] Neese, F.; Solomon, E. I. *Inorg. Chem.* **1999**, *38*, 1847–1865.

[19] Transue, W. J.; Snyder, R. A.; Caranto, J. D.; Kurtz, D. M. J.; Solomon, E. I. *Inorg. Chem.* **2022**, *61*, 16520–16527.

[20] Tanner, P. A.; Yeung, Y.-Y.; Ning, L. *J. Phys. Chem. A* **2014**, *118*, 8745–8752.

[21] Carnall, W. T.; Fields, P. R.; Rajnak, K. *J. Chem. Phys.* **1968**, *49*, 4424–4442.

[22] Carnall, W. T.; Goodman, G. L.; Rajnak, K.; Rana, R. S. *J. Chem. Phys.* **1989**, *90*, 3443–3457.

[23] Bain, G. A.; Berry, J. F. *J. Chem. Educ.* **2008**, *85*, 532.

[24] Neese, F. *WIREs Comput. Mol. Sci.* **2018**, *8*, e1327.

[25] Neese, F. *WIREs Comput. Mol. Sci.* **2012**, *2*, 73–78.

[26] Ganyushin, D.; Neese, F. *J. Chem. Phys.* **2006**, *125*, 024103.

[27] Stoychev, G. L.; Auer, A. A.; Neese, F. *J. Chem. Theory Comput.* **2017**, *13*, 554–562.

[28] Kollmar, C.; Sivalingam, K.; Helmich-Paris, B.; Angeli, C.; Neese, F. *J. Comput. Chem.* **2019**, *40*, 1463–1470.

[29] Lang, L.; Neese, F. *J. Chem. Phys.* **2019**, *150*, 104104.

[30] Kollmar, C.; Sivalingam, K.; Guo, Y.; Neese, F. *J. Chem. Phys.* **2021**, *155*, 234104.

[31] Guo, Y.; Sivalingam, K.; Neese, F. *J. Chem. Phys.* **2021**, *154*, 214111.

[32] Neese, F. *J. Comput. Chem.* **2023**, *44*, 381–396.

[33] Ugandi, M.; Roemelt, M. *Int. J. Quantum Chem.* **2023**, *123*, e27045.

[34] Pantazis, D. A.; Chen, X.-Y.; Landis, C. R.; Neese, F. *J. Chem. Theory Comput.* **2008**, *4*, 908–919.

[35] Weigend, F.; Ahlrichs, R. *Phys. Chem. Chem. Phys.* **2005**, *7*, 3297–3305.

[36] Mason, W. R. *A Practical Guide to Magnetic Circular Dichroism Spectroscopy*, 1st ed.; John Wiley & Sons, Inc.: Hoboken, NJ, 2007.
